# Intra-Individual Paired Mass Spectrometry Dataset for Decoding Solar-Induced Proteomic Changes in Facial Skin

**DOI:** 10.1038/s41597-024-03231-1

**Published:** 2024-05-03

**Authors:** Amanda C. Camillo-Andrade, Marlon D. M. Santos, Patrícia S. Nuevo, Ana B. L. Lajas, Lucas A. Sales, Alejandro Leyva, Juliana S. G. Fischer, Rosario Duran, Paulo C. Carvalho

**Affiliations:** 1 Laboratory for Structural and Computational Proteomics, Carlos Chagas Institute, Curitiba, Paraná Brazil; 2 Analytical Biochemistry and Proteomics Unit, Instituto de Investigaciones Biológicas Clemente Estable, Institut Pasteur de Montevideo, Montevideo, Uruguay; 3 Asthetics and Cosmetics, Positivo University, Curitiba, Paraná Brazil

**Keywords:** Proteome, Quality of life

## Abstract

Photoaging is the premature aging of the skin caused by prolonged exposure to solar radiation. The visual alterations manifest as wrinkles, reduced skin elasticity, uneven skin tone, as well as other signs that surpass the expected outcomes of natural aging. Beyond these surface changes, there is a complex interplay of molecular alterations, encompassing shifts in cellular function, DNA damage, and protein composition disruptions. This data descriptor introduces a unique dataset derived from ten individuals, each with a minimum of 18 years of professional experience as a driver, who are asymmetrically and chronically exposed to solar radiation due to their driving orientation. Skin samples were independently collected from each side of the face using a microdermabrasion-like procedure and analyzed on an Exploris 240 mass spectrometer. Our adapted proteomic statistical framework leverages the sample pairing to provide robust insights. This dataset delves into the molecular differences in exposed skin and serves as a foundational resource for interdisciplinary research in photodermatology, targeted skincare treatments, and computational modelling of skin health.

## Background & Summary

The skin is the body’s primary line of defense, forming a protective barrier against environmental factors. It also serves as a dynamic indicator of our body’s overall health. This complex organ plays a pivotal role in fluid conservation, temperature moderation, and sensory communication. Moreover, it houses a unique immunological zone crucial for tissue stability, protection, and repair^1^.

As the skin ages, a series of modifications become evident. The structural integrity begins to decline, leading to decreased elasticity and suppleness. The skin’s capacity to retain moisture diminishes, making it more susceptible to dehydration^2^. Furthermore, cellular regeneration decelerates, hindering the skin’s innate repair mechanisms. These shifts go beyond mere surface changes; they indicate foundational alterations in the skin’s functions. Its defense capabilities, thermal regulation, and sensory communication all begin to falter^3^.

The mechanisms of skin aging are primarily divided into chronological and premature pathways. Chronological or intrinsic aging is an inevitable process driven by genetics and time, resulting in reduced collagen production, diminished cellular turnover, and elastin degradation. On the other hand, premature or extrinsic aging is largely influenced by environmental stressors^4^. Key contributors include exposure to pollutants, lifestyle habits like smoking, and notably, solar radiation. Among these, solar radiation stands out as a paramount factor, inducing oxidative stress, and the breakdown of collagen and elastin structures, thereby accelerating the visible signs of skin aging^5^.

Solar radiation encompasses ultraviolet (UV), visible light (VL), and infrared radiation (IRA). UV is subdivided into UVC (100–280 nm), UVB (280–320 nm), and UVA (320–400 nm), both UVA and UVB have known effects on skin health^6^. UV exposure leads to photoaging, characterized by skin roughness and age spots, while VL, especially blue-violet light (around 415 nm), can induce prolonged pigmentation. At the cellular level, both UV and VL produce reactive oxygen species, damaging DNA, proteins, and lipids^7^. IRA also affects dermal structure and skin lipid composition^5^. Given these varied effects, understanding the skin’s molecular changes due to different solar components is crucial for maintaining skin health^8^.

In response of the intricate nature of these molecular dynamics, proteomic advancements over the past decade have revolutionized our understanding of skin conditions and the underlying molecular mechanisms^9^. Utilizing mass spectrometry, proteomics not only pinpoints and measures individual proteins but also offers insights on post-translational modifications, protein interactions, and pathways. This technique has been particularly effective in deciphering the complex molecular mechanisms behind photoaging and associated skin disorders; however, despite its groundbreaking potential, the incorporation of proteomics into dermatological research hasn’t kept pace with its technological evolution^10^.

Proteomic studies in humans represent significant challenges, primarily due to the inherent complexity and diversity of the human proteome^10^. Obtaining consistent and representative human samples is a considerable hurdle, compounded by inter-individual variability and the dynamic nature of protein expression in response to various internal and external factors. The importance of employing paired studies to achieve robust scientific outcomes has been demonstrated in diverse areas of proteomic research, including a notable study on breast cancer^11^. In the latter, nipple aspirate fluid was collected from breasts both with and without cancer across different participants. The application of paired statistical approaches was instrumental in identifying differentially abundant proteins, thereby underscoring the nature of paired datasets^12^.

With the motivation of pairing samples to minimize inter-individual variability and maximize result reliability, this dataset focuses on photoaging among professional drivers. All drivers belonging to this dataset have a minimum of 18 years of professional experience, are non-smokers, and where particularly susceptible to asymmetric solar exposure due to the orientation of their driver-side windows. This chronic, side-specific exposure to solar radiation makes them an ideal cohort for photoaging studies, allowing for a nuanced understanding of the effects of the exposure. In our research, we used a microdermabrasion-like, non-invasive, and painless skin collection technique, that, through mass spectrometry, can offer a proteomic overview of changes associated with photoaging.

The quality control of our dataset was performed using RawVegetable, a tool designed for the nuanced evaluation of mass spectrometry data. Among its features are the charge state chromatogram and TopN density estimation modules, which aided in honing our chromatography processes^13^. Furthering our analysis, we utilize PatternLab for Proteomics V (PLV) to gauge the quantity of peptides and estimate the number of proteins in the sample. PLV’s suite of integrated tools offers an intricate view of the sample’s proteomic landscape. Leveraging its peptide spectrum matching and data filtering capabilities, we’re poised to both identify and quantify peptides, resulting in precise protein identification^14^. Finally, we also employed DiagnoMass^15^; this tool relies on spectral clustering to perform sample comparison without the bias of the search engine (in this case, PLV). As such, DiagnoMass allows us to probe how much of the proteome is discriminative between the two conditions and what proportion of it was missed by PLV, thus revealing how much is yet to be explored from our contribution^16^.

We believe this dataset will not only shed light on molecular differences in exposed skin, especially between the right (lower exposure) and left (higher exposure) sides of the face, but also provide a robust foundation for future interdisciplinary investigations in photodermatology and beyond.

## Methods

### Sample collection

This study was approved by the Fiocruz Research Ethics Committee (CAAE 38352020.8.0000.5248. Male Caucasian participants, aged between 35 and 70 years and with a phototype ranging from II to IV, met our anamnesis criteria, which included specific inclusion and exclusion standards. Exclusion criteria encompassed conditions such as skin diseases, smokers, or having diabetes. All participants provided written informed consent for data collection and sharing. Prior to sample collection, the skin was thoroughly cleaned with a cotton pad soaked in micellar water to remove surface contaminants and excess oils. We then employed a microdermabrasion technique using the Dermotonus Slim Vacuum Therapy equipment produced by Ibramed to gently exfoliate the skin without causing harm or discomfort; the exfoliated skin is trapped in a 3D printed device adapted by us (under patent) for attachment to the equipment and to minimize sample manipulation. Each participant provided one sample from each side of their face, resulting in a total of 20 samples across 10 participants.

### Sample preparation

The skin samples were subjected to lysis for 10 vortex cycles using the equipment (FlexVortex 2 – Loccus) at maximum intensity. The first five cycles incorporated 0.1 mm zirconium beads (Loccus), and the remaining cycles also incorporated RapiGest detergent at a concentration of 0.1%, following the manufacturer’s recommendations. Each cycle involved one minute of vortexing followed by one minute of cooling on ice.

The skin proteins extraction was performed with RapiGest detergent at a concentration of 0.1% according to the manufacturer’s recommendations. One hundred micrograms of proteins from each sample were reduced with dithiothreitol (DTT) (final concentration of 10 mM) for 30 min, at 60 °C. After being cooled to room temperature, the samples were alkylated with iodoacetamide (final concentration of 30 mM) for 25 min at room temperature, in the dark, and finally digested with high sequence grade modified trypsin in the proportion of 1/50 (E/S) for 20 h, at 37 °C.

### Desalting and sample quantification

In due course, the enzymatic reaction was stopped by adding trifluoroacetic (0.4% v/v final) and the peptides were incubated for additional 40 min to degrade the RapiGest. Afterward, the samples were centrifuged at 18,000 g for 10 min to remove any insoluble materials. Subsequently, the peptides were quantified using the fluorometric assay—Qubit 2.0 (Invitrogen) according to the manufacturer’s recommendations. Each sample was desalted and concentrated using Stage-Tips (STop and Go-Extraction TIPs)^17^.

### Mass spectrometry analysis

Each peptide mixture was twice subjected to reversed-phase liquid chromatography followed by tandem mass spectrometry (LC–MS/MS) analysis with an UltiMate 3000 nanoHPLC (Thermo Scientific®) coupled online with an Exploris 240 Orbitrap mass spectrometer (Thermo Scientific®). The peptide mixture was chromatographically separated on a column (15 cm in length with a 75 μm I.D., C18-AQ 3.0 μm resin, SNC20442712 - Thermo Scientific) with a flow of 250 nL/min from 1% to 40% ACN (acetonitrile) in 0.1% formic acid, in a 120 min gradient. The Exploris 240 Orbitrap was set to the data-dependent acquisition (DDA) mode to automatically switch between full scan MS and MS/MS acquisition with 30 s dynamic exclusion. Survey scans (200–2000 *m/z*) were acquired in the Orbitrap system with a resolution of 60,000 at *m/z* 200. The most intense ions captured in a 2 s cycle time were selected, excluding those unassigned and in a 1 + charge state, sequentially isolated and HCD (Higher-energy collisional dissociation) fragmented using a stepped normalized collision energy of 25, 30, and 35. The fragment ions were analyzed with a resolution of 15,000 at 200 *m/z*. The general mass spectrometric conditions were as follows: 2.5 kV spray voltage, no sheath or auxiliary gas flow, heated capillary temperature of 40 °C, predictive automatic gain control (AGC) enabled, and an S-lens RF level of 40%. Mass spectrometer scan functions and nLC solvent gradients were controlled by the Xcalibur 4.1 data system (Thermo Scientific®).

### Peptide spectrum matching (PSM)

The data analysis was performed with the PatternLab for proteomics V (PLV) software that is freely available at https://www.patternlabforproteomics.org 14. Homo sapiens’ sequences were downloaded on July 7th, 2023, from the Swiss-Prot and then a target-decoy database was generated to include a reversed version of each sequence plus those from 104 common mass spectrometry contaminants. The data was preprocessed with the Y.A.D.A. 3.0 deconvolution algorithm to enable multiplexed spectra identification^18^. Comet 2021 search engine^19^, which is embedded into PLV, was used for identifying the mass spectra. The search parameters considered: fully and semi-tryptic peptide candidates with masses between 500 and 6000 Da, up to two missed cleavages, 35 ppm for precursor mass, and bins of 0.02 m/z for MS/MS. The modifications were carbamidomethylation of cysteine and oxidation of methionine as fixed and variable, respectively.

### Validation PSM

The validity of the PSMs was assessed using Search Engine Processor (SEPro)^20^. The identifications were grouped by charge state (2 + and ≥ 3 + ), and then by tryptic status, resulting in four distinct subgroups. For each group, the XCorr, DeltaCN, DeltaPPM, and Peaks Matches values were used to generate a Bayesian discriminator. The identifications were sorted in nondecreasing order according to the discriminator score. A cutoff score accepted a false-discovery rate (FDR) of 2% at the peptide level based on the number of decoys^21^. This procedure was independently performed on each data subset, resulting in an FDR independent of charge state or tryptic status. Additionally, a minimum sequence length of five amino-acid residues and a protein score greater than 3 were imposed. Finally, identifications deviating by more than 10 ppm from the theoretical mass were discarded. These last filters led to FDRs, now at the protein level, to be lower than 1% for all search results.

## Data Records

The mass spectrometry proteomics data have been deposited to the ProteomeXchange Consortium via the PRIDE^22^ partner repository with the dataset identifier PXD045887^23^.

## Technical Validation

### Dataset quality control with RawVegetable 2.0

**Fig. 1:**
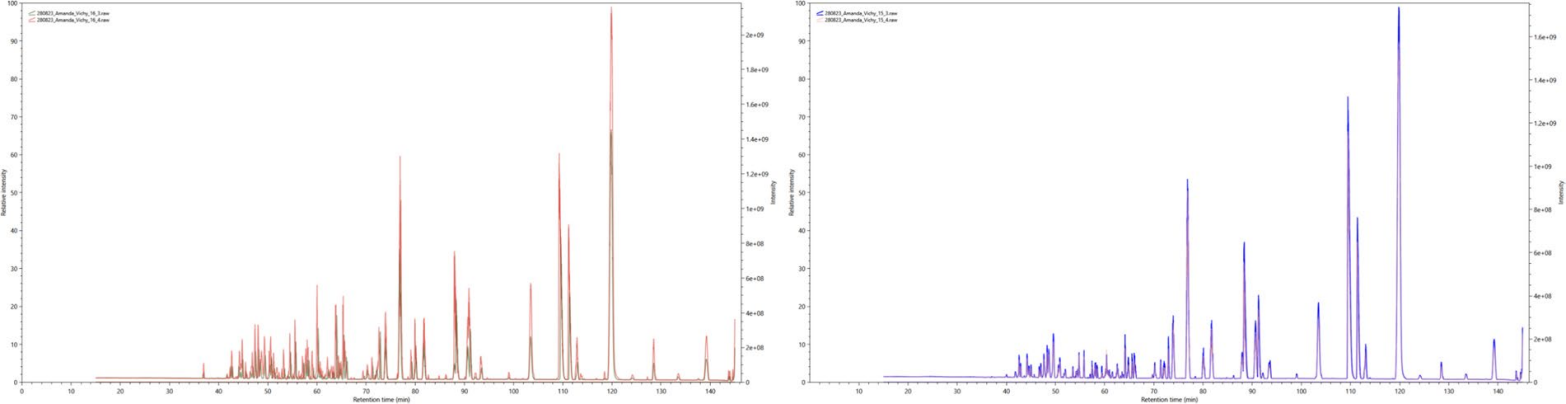
Ion chromatogram comparison for technical replicates: face skin Left and Right sides of the same driver.

To assess the quality of our mass spectrometry data, especially when comparing experimental technical replicates, we employed RawVegetable, a specialized software tool designed for mass spectrometry data assessment^13^. We employed several functionalities of this tool that are now described. The charge state chromatogram module and the TopN density estimation module allowed us to optimize our chromatography before generating the final dataset. TopN is notably significant as it governs the number of MS/MS scans generated per cycle. This feature aided in pinpointing retention time intervals where under-sampling or over-sampling occurred, making gradient adjustments more straightforward. The chromatography reproducibility module enabled direct comparisons across experiments, ensuring data consistency as shown in Fig. 1. A detailed quality control analysis with images for each replicate pair can be found in ‘Supplementary_QC.docx’ file. Finally, we assessed the quality of MS/MS spectra by examining their Xrea scores^24^ throughout the run and using the precursor signal ratio distribution to gauge fragmentation efficiency.

We provide Table 1, that summarizes the number of identifications provided by PLV on our samples. Details regarding the identification can be found in the ‘Supplementary_ID.rar’ file.

**Table 1:** Bilateral facial analysis, encompassing spectra, peptides, and proteins.

Number	Side	ID	Peptides	Proteins	Driving years	Driver’s Age
1	Right	7	4,591	894	30	49
	Left	8	9,909	1,434		
2	Right	9	5,149	878	35	54
	Left	10	5,181	873		
3	Right	15	3,105	706	30	54
	Left	16	4,766	880		
4	Right	17	6,248	1017	24	47
	Left	18	5,066	896		
5	Right	19	4,301	871	18	43
	Left	20	5,946	992		
6	Right	21	5,813	968	30	48
	Left	22	2,282	597		
7	Right	23	3,041	636	25	54
	Left	24	3,747	581		
8	Right	29	2,494	615	18	49
	Left	30	5,999	1018		
9	Right	31	4,631	924	18	53
	Left	32	4,034	818		
10	Right	33	1,245	483	20	37
	Left	34	1,291	430		

**Number:** Sequential data numbering; **Side:** Facial side (Right or Left); **ID:** Sample’s Unique identifier; **Driving years:** Associated with professional driving years; **Driver’s age:** The age of the individual at the time of sample acquisition.

Our mass spectrometry analysis generated a total of 40 raw files, comprising 2,560,226 mass spectra. These spectra were clustered into 240,369 unique spectral clusters using DiagnoMass software. It is important to note that peptides with identical sequences, but different charge states were classified into separate clusters, suggesting that the actual number of unique biological molecules could be approximately half of the total clusters. The hierarchical clustering algorithm employed by DiagnoMass required a minimum spectral angle of 0.75 for spectra to be grouped together. Spectra that did not meet this criterion were excluded from the analysis. We considered only those clusters containing three or more spectra with a spectral angle greater than or equal to 0.75. The results of this analysis are summarized in Table 2, which also outlines the number of these spectral clusters subsequently identified by PLV.

**Table 2:** Comparative Analysis of Spectral Clusters Uniquely found in Solar-Exposed and Non-Exposed Facial Skin Samples.

Number of Replicates	Spectral Clusters (Solar-Exposed)	Spectral Clusters (Non-Exposed)	Identified Clusters (Solar-Exposed)	Identified Clusters (Non-Exposed)
1	10,350	8,058	423	284
2	8,773	6,851	366	255
3	3,709	3,023	185	124
4	631	545	40	30
5	112	85	5	3
6	27	17	2	2
7	3	3	1	1
8	0	1	0	0
9	0	0	0	0
10	0	0	0	0

This table provides a comprehensive breakdown of the spectral clusters identified in the skin samples, focusing on the differences between the solar-exposed (left side) and non-exposed (right side) areas of the face. It enumerates the number of unique spectral clusters detected using DiagnoMass and further delineates the number of these clusters identified by PatternLab. The columns are labeled as follows: ‘Number of Replicates’ indicates the number of biological replicates that exhibited the corresponding count of unique spectral clusters; for instance, 3,709 unique spectral clusters were identified exclusively on the solar-exposed left side in three or more biological replicates. ‘Spectral Clusters (Solar-Exposed)’ and ‘Spectral Clusters (Non-Exposed)’ represent the number of unique spectral clusters identified on the solar-exposed left and non-exposed right sides of the face, respectively. ‘Identified Clusters (Solar-Exposed)’ and ‘Identified Clusters (Non-Exposed)’ specify the number of spectral clusters from each side that were identified by PatternLab. The table serves as a critical resource for understanding the proteomic alterations induced by solar exposure, thereby providing a foundation for future research in photodermatology and targeted skincare treatments.

## Usage Notes

Our updated and comprehensive dataset on proteomic changes resulting from solar exposure in facial skin provides not only a profound insight into direct protein alterations and broader implications of the exposome but also lays the foundation for understanding photoaging, investigating the links between solar exposure and skin diseases, developing personalized treatments, researching the combined effects of environmental factors on skin health, and assisting cosmetic and pharmaceutical industries in refining products to address specific protein changes. We now suggest 3 usage cases for this dataset.

### Advancements in photodermatology research

The dataset is particularly useful for researchers in the field of photodermatology. It can serve as a foundational resource for understanding the molecular mechanisms that underlie solar-induced skin aging. Researchers can employ this dataset to validate existing theories or generate new hypotheses on how prolonged solar exposure leads to specific proteomic alterations.

### Development of targeted skincare treatments

Personalized skincare has become an area of burgeoning interest in both the cosmetic and pharmaceutical industries. The proteomic data can be used to identify key proteins or pathways that are differentially regulated due to solar exposure. This information ultimately aids in the development of targeted treatments that can either upregulate or downregulate specific proteins to mitigate the effects of photoaging. For example, if a certain protein is less abundant in solar-exposed skin, a treatment could be formulated to boost its expression, thereby potentially slowing the aging process at the molecular level. However, we acknowledge the limitations posed by the dataset’s size and suggest that these findings serve as an exploratory step toward more comprehensive studies. Further research with expanded cohorts is essential to develop reliable, personalized skincare treatments based on proteomics.

### Computational modeling and algorithm development

The dataset’s high complexity and dimensionality make it an invaluable resource for scholars in the fields of computational biology and bioinformatics. Its unique structure provides fertile ground for the development of innovative algorithms tailored for the analysis of paired mass spectrometry data, a critical aspect for ensuring robust statistical outcomes. Moreover, the dataset can be amalgamated with other “omics” data types, such as genomics or transcriptomics, to formulate multi-layered computational models that deepen our understanding of skin health and aging processes.

A noteworthy aspect is the role of DiagnoMass in this dataset’s utility. The tool reveals that only a minor fraction of spectral clusters unique to solar exposed versus non-exposed conditions are currently identified. This suggests the existence of potentially undiscovered alterations not yet cataloged in public databases, or post-translational modifications that present detection challenges for existing tools. DiagnoMass thus serves as a critical benchmark, setting an upper limit on what is currently known and highlighting the expansive scope for future discoveries in skin proteomics.

### Supplementary information

Supplementary File Peptides and Proteins

Supplementary Quality Control

Supplementary_ID

## Data Availability

In this study, no custom code was utilized. All software used in this study is open access. A comprehensive list of software used in this study is provided in this section as well. • PatternLab for Proteomics V (http://patternlabforproteomics.org/). • DiagnoMass (https://www.diagnomass.com/). • RawVegetable (http://patternlabforproteomics.org/rawvegetable/).
