# Supplementary material for: Intra-Individual Paired Mass Spectrometry Dataset for Decoding Solar-Induced Proteomic Changes in Facial Skin
Source: Sci Data. 2024 May 3;11:441. doi: 10.1038/s41597-024-03231-1 (PMC11068864; doi:10.1038/s41597-024-03231-1)
Supplement: Supplementary file 1 — Supplementary File Peptides and Proteins [file 41597_2024_3231_MOESM1_ESM.docx]

**Intra-Individual Paired Mass Spectrometry Dataset for Decoding Solar-Induced Proteomic Changes in Facial Skin**

**Authors**

Amanda C. Camillo-Andrade^1,2,3^, Marlon D. M. Santos ^1,2^, Patrícia S. Nuevo^3^, Ana B. L. Lajas^1^, Lucas A. Sales^1^, Alejandro Leyva^2^, Juliana S. G. Fischer^1^, Rosario Duran^2*^ & Paulo C. Carvalho^1*^

**Affiliations**

1. Laboratory for Structural and Computational Proteomics, Carlos Chagas Institute, Fiocruz, Paraná, Brazil
2. Analytical Biochemistry and Proteomics Unit, Instituto de Investigaciones Biológicas Clemente Estable, Institut Pasteur de Montevideo, Montevideo, Uruguay
3. Asthetics and Cosmetics, Positivo University, Paraná, Brazil

* Corresponding author(s): Rosario Durán (duran@pasteur.edu.uy); Paulo C. Carvalho (paulo@pcarvalho.com)

Venn Diagrams illustrate the overlap of technical replicates at both the peptide and protein levels, providing a more detailed view of the quality control process.

- 07

| Peptide Technical Replicates | Protein Technical Replicates |
| --- | --- |
| 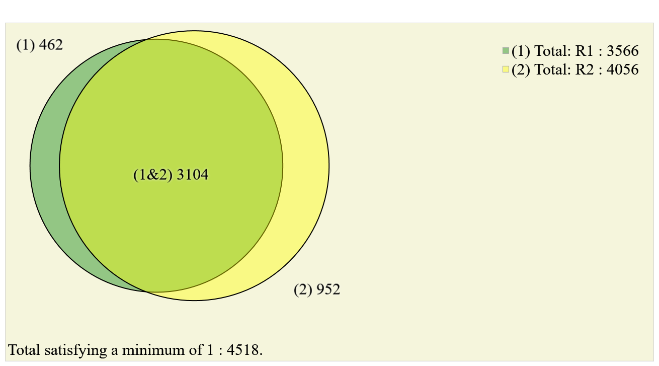 | 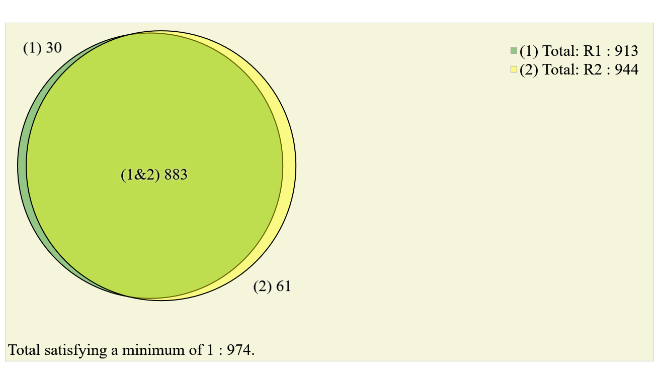 |

- 08

| Peptide Technical Replicates | Protein Technical Replicates |
| --- | --- |
| 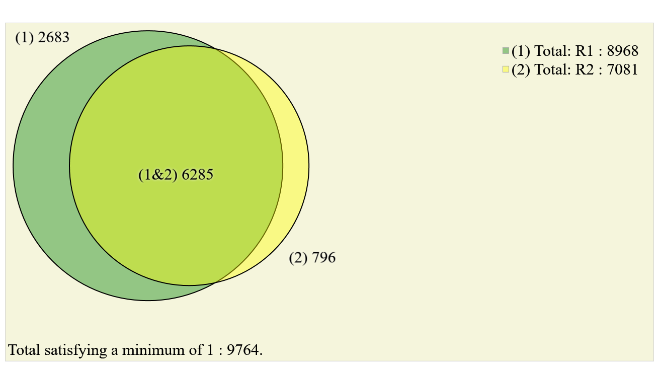 | 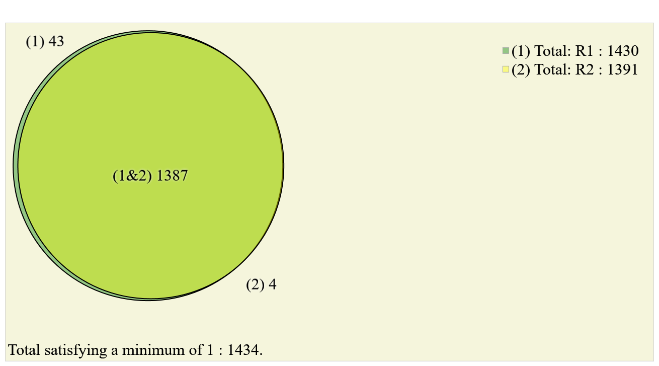 |

- 09

| Peptide Technical Replicates | Protein Technical Replicates |
| --- | --- |
| 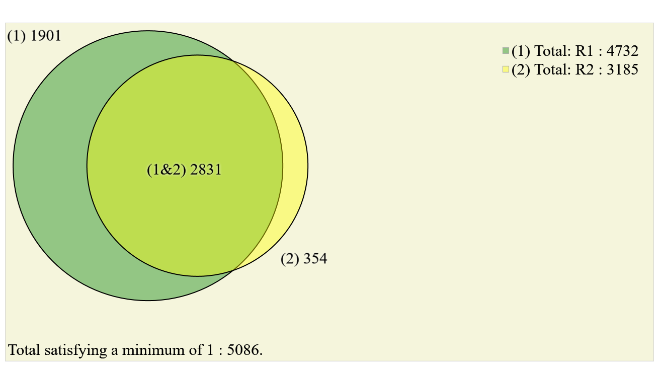 | 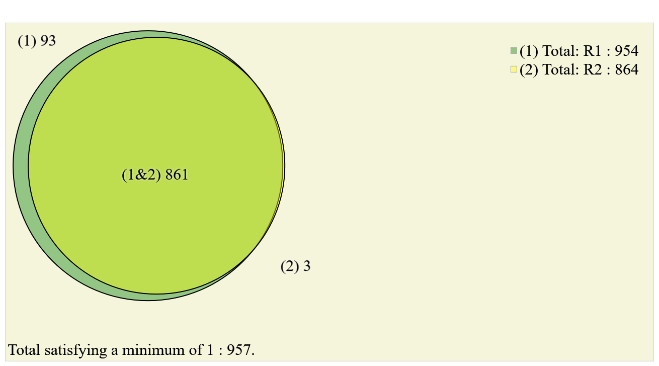 |

- 10

| Peptide Technical Replicates | Protein Technical Replicates |
| --- | --- |
| 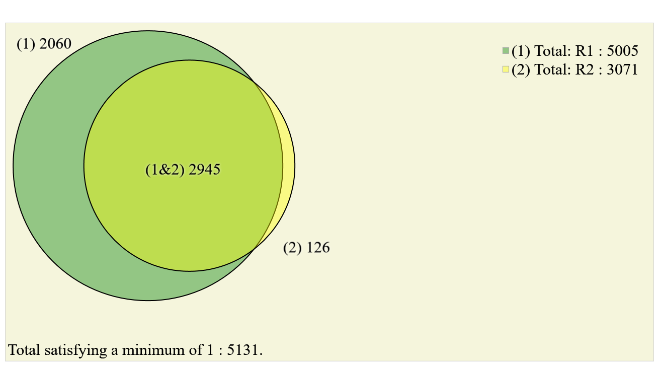 | 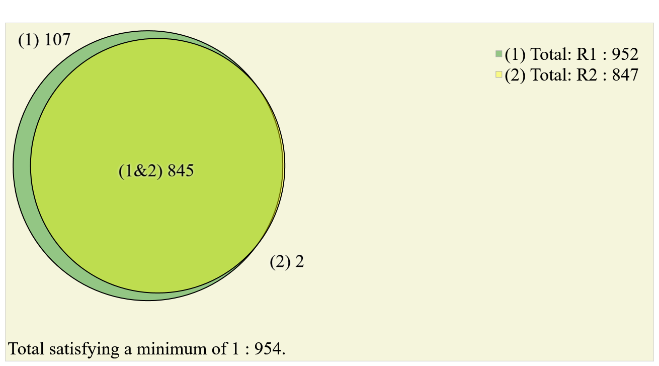 |

- 15

| Peptide Technical Replicates | Protein Technical Replicates |
| --- | --- |
| 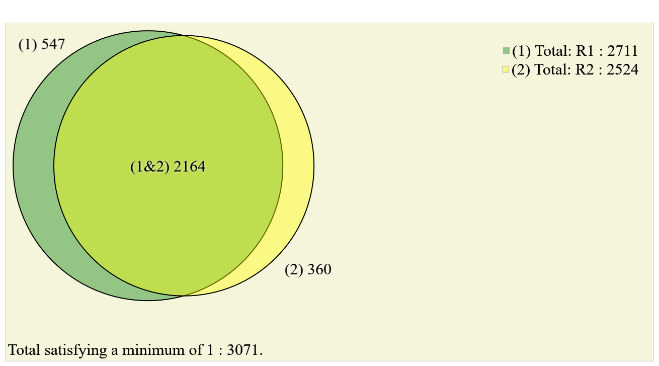 | 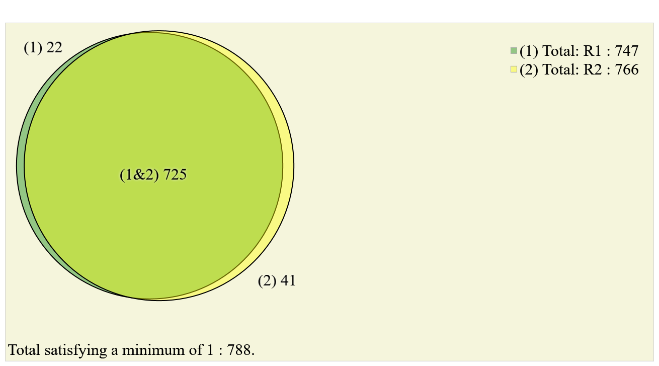 |

- 16

| Peptide Technical Replicates | Protein Technical Replicates |
| --- | --- |
| 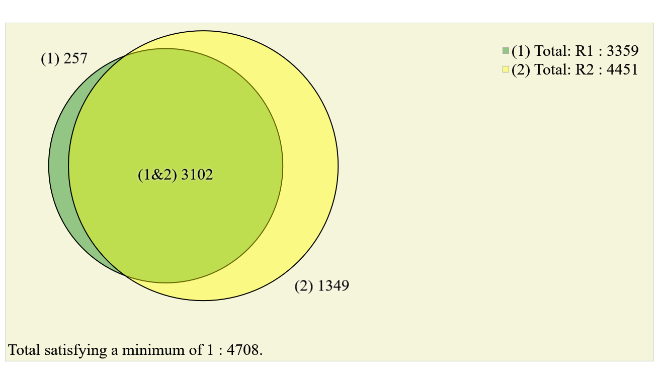 | 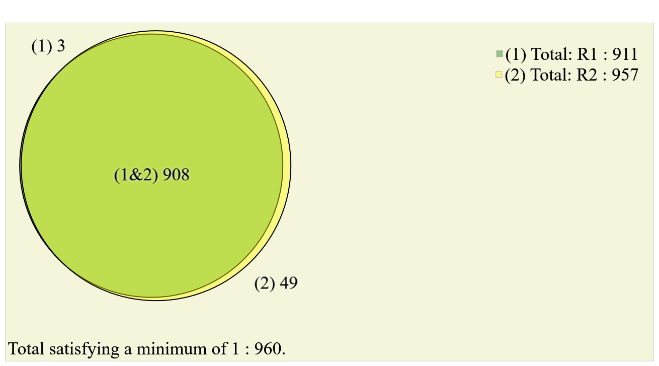 |

- 17

| Peptide Technical Replicates | Protein Technical Replicates |
| --- | --- |
| 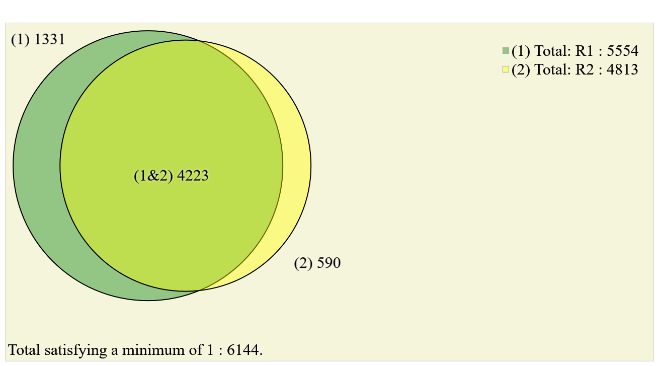 | 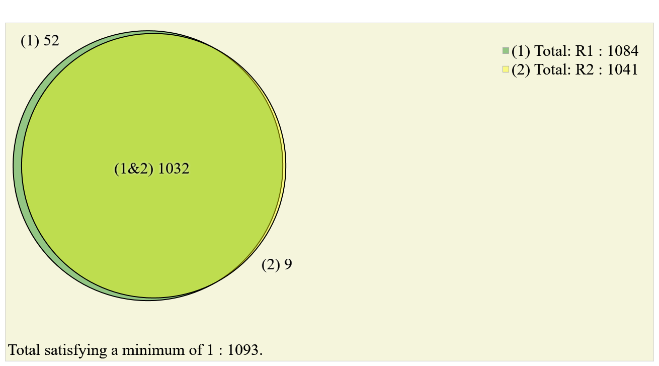 |

- 18

| Peptide Technical Replicates | Protein Technical Replicates |
| --- | --- |
| 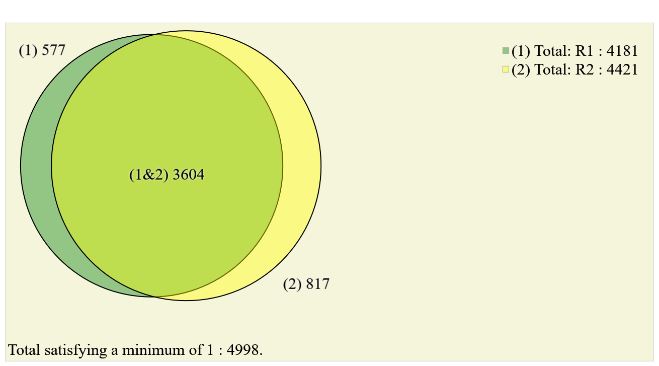 | 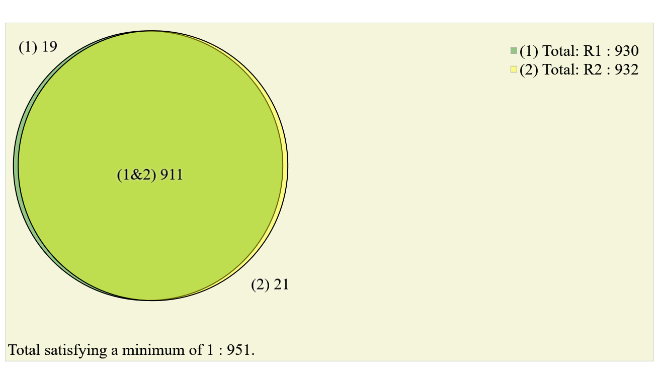 |

- 19

| Peptide Technical Replicates | Protein Technical Replicates |
| --- | --- |
| 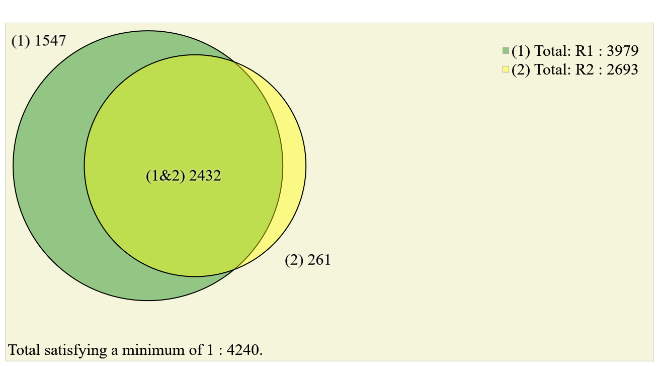 | 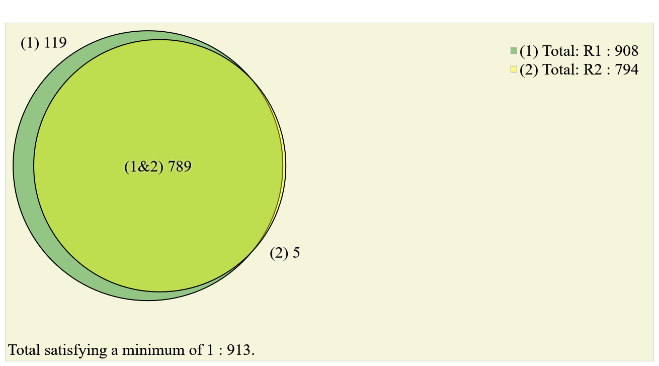 |

- 20

| Peptide Technical Replicates | Protein Technical Replicates |
| --- | --- |
| 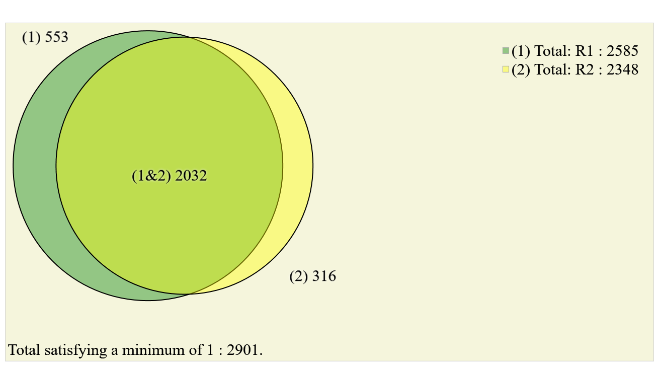 | 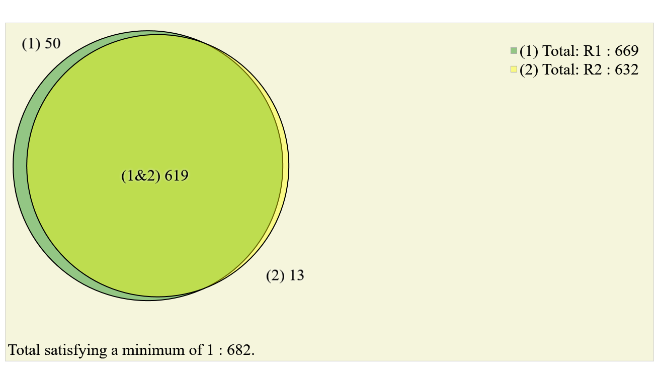 |

- 21

| Peptide Technical Replicates | Protein Technical Replicates |
| --- | --- |
| 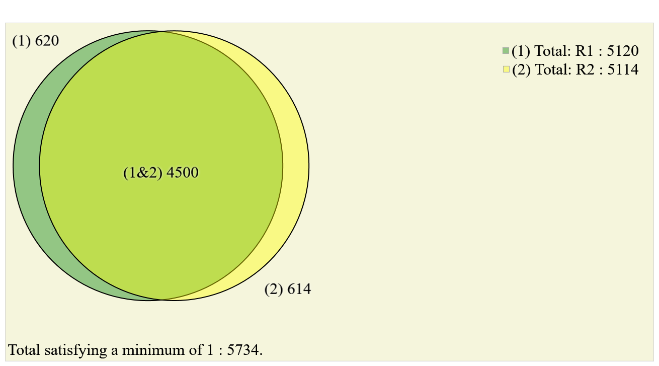 | 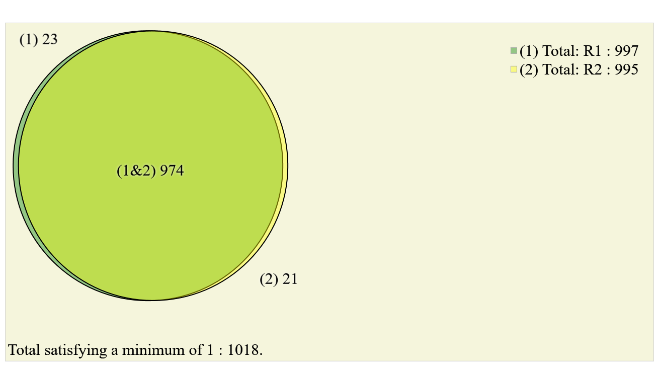 |

- 22

| Peptide Technical Replicates | Protein Technical Replicates |
| --- | --- |
| 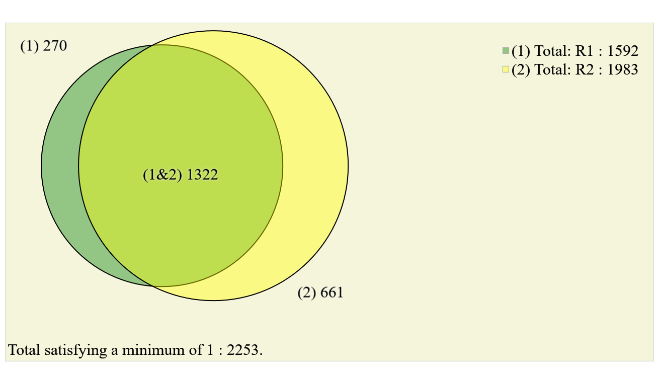 | 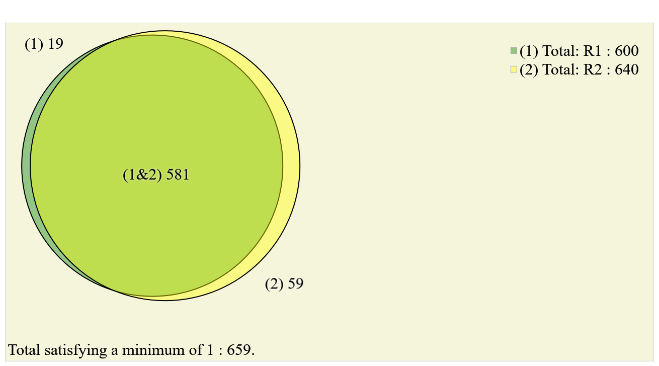 |

- 23

| Peptide Technical Replicates | Protein Technical Replicates |
| --- | --- |
| 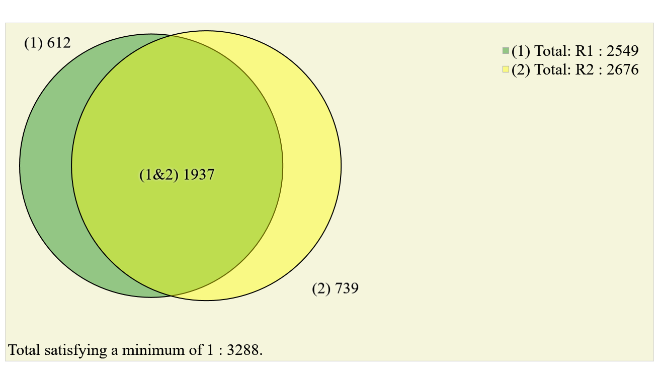 | 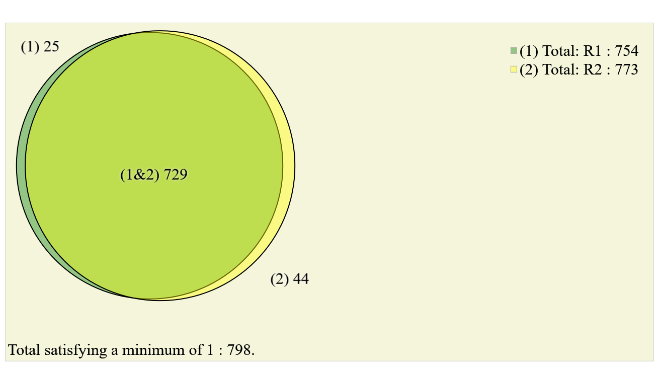 |

- 24

| Peptide Technical Replicates | Protein Technical Replicates |
| --- | --- |
| 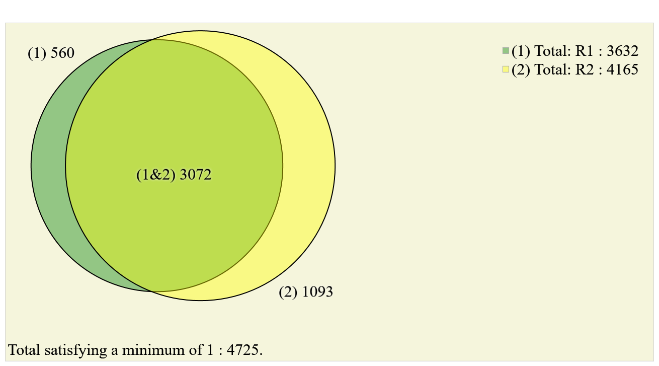 | 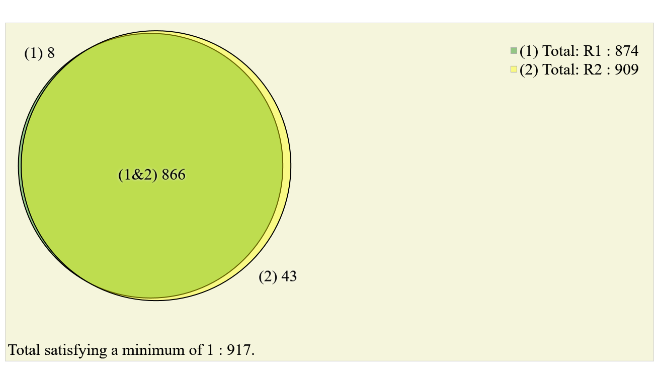 |

- 29

| Peptide Technical Replicates | Protein Technical Replicates |
| --- | --- |
| 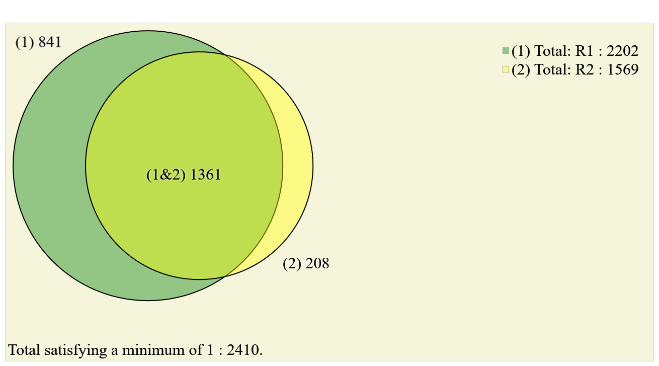 | 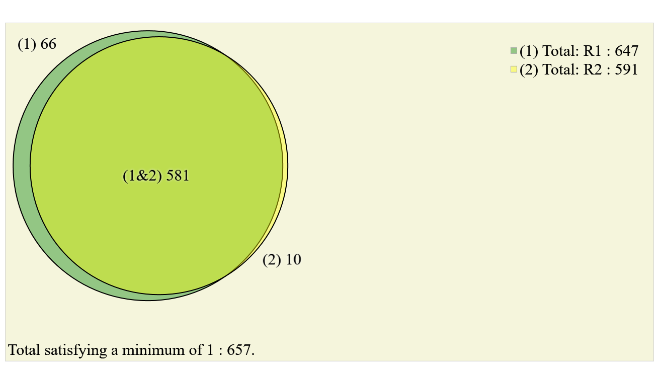 |

- 30

| Peptide Technical Replicates | Protein Technical Replicates |
| --- | --- |
| 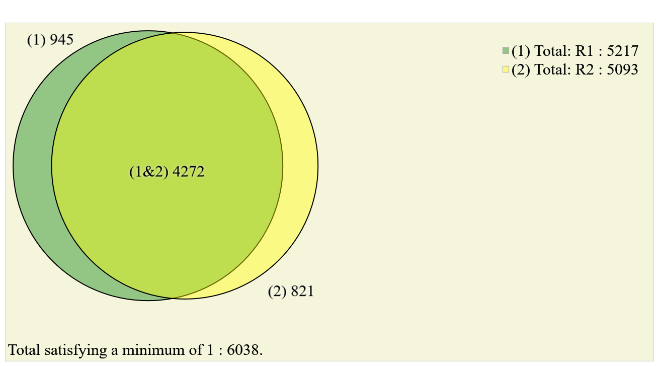 | 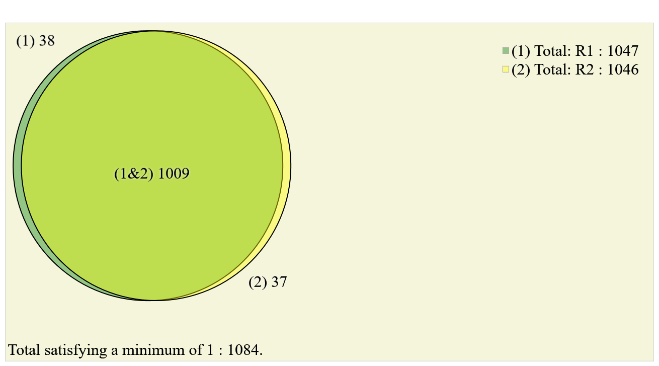 |

- 31

| Peptide Technical Replicates | Protein Technical Replicates |
| --- | --- |
| 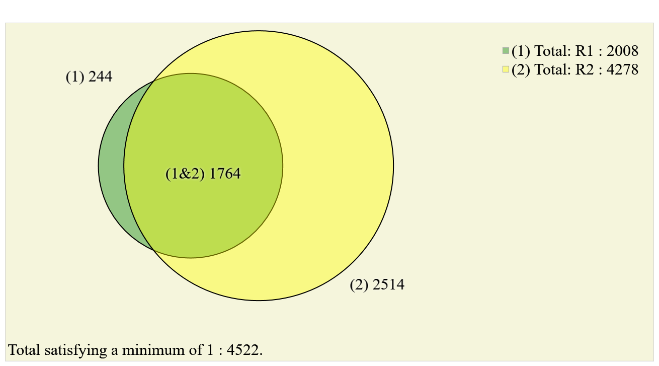 | 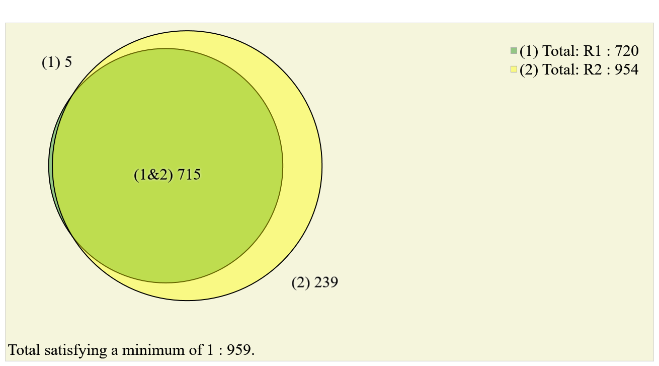 |

- 32

| Peptide Technical Replicates | Protein Technical Replicates |
| --- | --- |
| 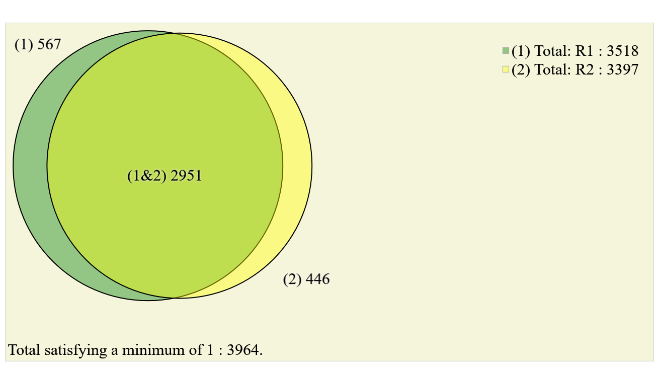 | 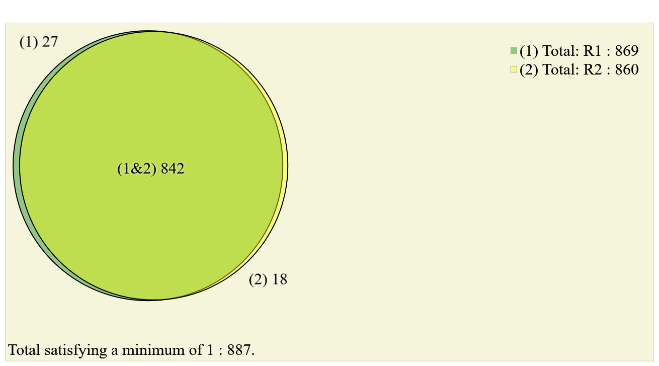 |

- 33

| Peptide Technical Replicates | Protein Technical Replicates |
| --- | --- |
| 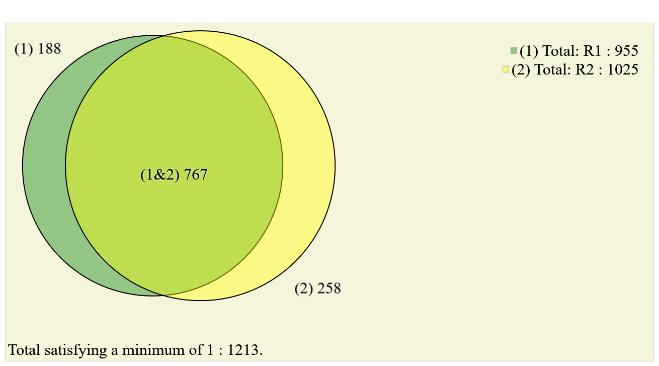 | 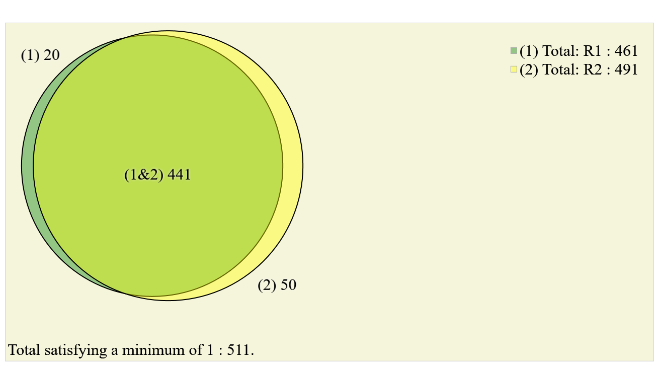 |

- 34

| Peptide Technical Replicates | Protein Technical Replicates |
| --- | --- |
| 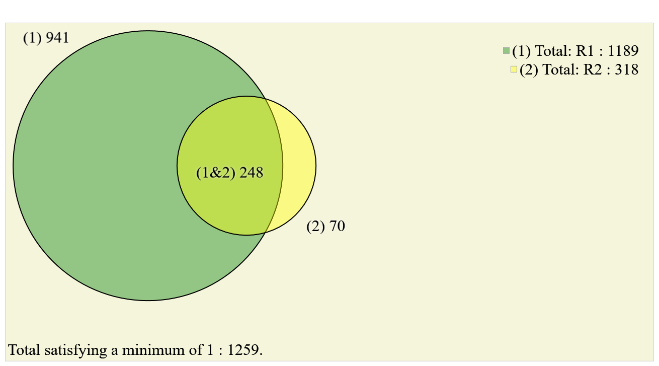 | 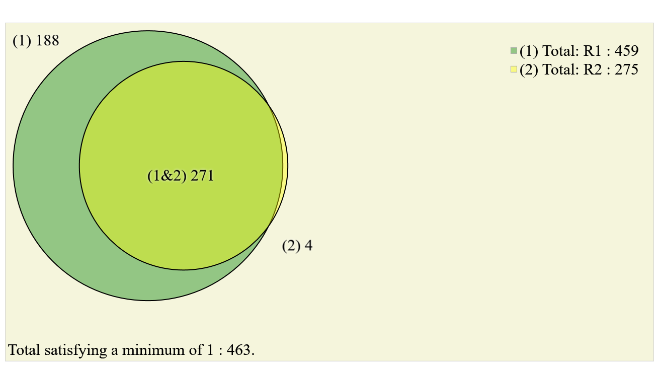 |
