# Supplementary material for: Intra-Individual Paired Mass Spectrometry Dataset for Decoding Solar-Induced Proteomic Changes in Facial Skin
Source: Sci Data. 2024 May 3;11:441. doi: 10.1038/s41597-024-03231-1 (PMC11068864; doi:10.1038/s41597-024-03231-1)
Supplement: Supplementary file 2 — Supplementary Quality Control [file 41597_2024_3231_MOESM2_ESM.docx]

**Intra-Individual Paired Mass Spectrometry Dataset for Decoding Solar-Induced Proteomic Changes in Facial Skin**

### Authors

Amanda C. Camillo-Andrade^1,2,3^, Marlon D. M. Santos ^1,2^, Patrícia S. Nuevo^3^, Ana B. L. Lajas^1^, Lucas A. Sales^1^, Alejandro Leyva^2^, Juliana S. G. Fischer^1^, Rosario Duran^2*^ & Paulo C. Carvalho^1*^

**Affiliations**

1. Laboratory for Structural and Computational Proteomics, Carlos Chagas Institute, Fiocruz, Paraná, Brazil
2. Analytical Biochemistry and Proteomics Unit, Instituto de Investigaciones Biológicas Clemente Estable, Institut Pasteur de Montevideo, Montevideo, Uruguay
3. Asthetics and Cosmetics, Positivo University, Paraná, Brazil

* Corresponding author(s): Rosario Durán (duran@pasteur.edu.uy); Paulo C. Carvalho (paulo@pcarvalho.com)

**Supplementary Material – Quality Control Assessment**

The quality control of our dataset was ensured using the RawVegetable (1) software, developed by our team, which offers a suite of graphical modules for detecting potential issues with the MS runs. This ensures the reliability of the data sent for peptide identification.

Our dataset comprises two distinct conditions: left and right sides of face from professional experience as a driver. Each condition includes 20 skins profiled via mass spectrometry, run in technical duplicate, resulting in a total of 40 raw files for analysis. **Table 1** provides a summary of all runs, including the number of MS and MS/MS scans generated, the average duty cycle time in milliseconds, and the full chromatography time. As depicted in **Figures 1, 2 and 3**, all runs exhibit similar numbers of scans, duty cycle times, and chromatography times, ensuring consistency across the dataset.


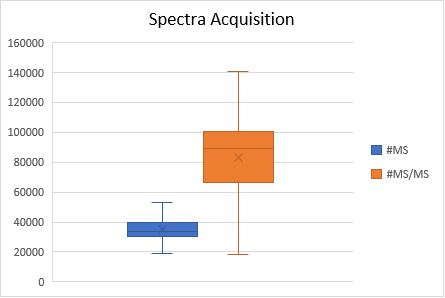


**Figure 1** - Statistical summary, in the form of a boxplot, of the number of MS and MS/MS spectra acquisition in all samples.


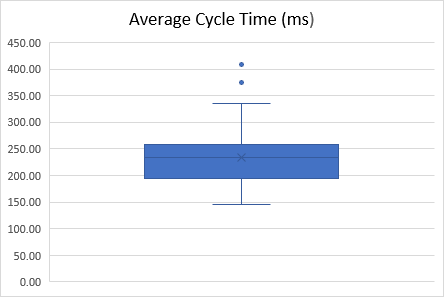


**Figure 2** - Boxplot of the average cycle time, of the MS run of all samples.


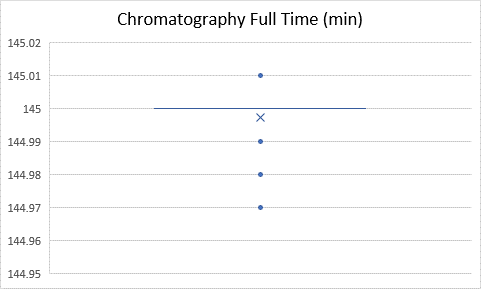


**Figure 3** - Boxplot of the chromatography full time of all samples.

**Table 1** - General information of all files generated in the dataset studied.

| \| File Name \| #MS \| #MS/MS \| Average Cycle Time (ms) \| Chromatography Full Time (min) \| \| --- \| --- \| --- \| --- \| --- \| \| 280823_Amanda_Vichy_08_1.raw \| 23214 \| 125522 \| 336.00 \| 144.99 \| \| 280823_Amanda_Vichy_08_2.raw \| 27674 \| 109697 \| 281.85 \| 145.00 \| \| 280823_Amanda_Vichy_10_1.raw \| 30815 \| 98209 \| 253.14 \| 145.01 \| \| 280823_Amanda_Vichy_10_2.raw \| 38016 \| 73013 \| 205.18 \| 145.00 \| \| 280823_Amanda_Vichy_16_3.raw \| 38593 \| 71080 \| 202.12 \| 145.01 \| \| 280823_Amanda_Vichy_16_4.raw \| 35315 \| 82423 \| 220.88 \| 145.00 \| \| 280823_Amanda_Vichy_18_1.raw \| 28570 \| 106395 \| 273.00 \| 144.99 \| \| 280823_Amanda_Vichy_18_2.raw \| 28038 \| 108296 \| 278.19 \| 145.00 \| \| 280823_Amanda_Vichy_20_3.raw \| 37741 \| 74000 \| 206.68 \| 145.00 \| \| 280823_Amanda_Vichy_20_4.raw \| 40091 \| 65700 \| 194.56 \| 145.00 \| \| 280823_Amanda_Vichy_22_1.raw \| 41759 \| 59887 \| 186.79 \| 145.00 \| \| 280823_Amanda_Vichy_22_2.raw \| 39543 \| 67570 \| 197.26 \| 145.00 \| \| 280823_Amanda_Vichy_24_2.raw \| 33749 \| 88094 \| 231.12 \| 145.00 \| \| 280823_Amanda_Vichy_24_3.raw \| 31060 \| 97420 \| 251.13 \| 145.00 \| \| 280823_Amanda_Vichy_30_1.raw \| 19069 \| 140539 \| 409.05 \| 145.00 \| \| 280823_Amanda_Vichy_30_2.raw \| 20795 \| 134417 \| 375.02 \| 144.97 \| \| 280823_Amanda_Vichy_32_1.raw \| 32816 \| 91204 \| 237.66 \| 144.98 \| \| 280823_Amanda_Vichy_32_2.raw \| 33228 \| 89774 \| 234.74 \| 144.99 \| \| 280823_Amanda_Vichy_34_1.raw \| 49608 \| 31873 \| 157.24 \| 145.00 \| \| 280823_Amanda_Vichy_34_2.raw \| 53305 \| 18613 \| 146.33 \| 145.00 \| \| 280823_Amanda_Vichy_07_1.raw \| 33588 \| 88791 \| 232.23 \| 145.00 \| \| 280823_Amanda_Vichy_07_2.raw \| 31041 \| 97663 \| 251.28 \| 145.00 \| \| 280823_Amanda_Vichy_09_1.raw \| 35641 \| 81260 \| 218.86 \| 145.00 \| \| 280823_Amanda_Vichy_09_2.raw \| 41315 \| 61181 \| 188.80 \| 145.00 \| \| 280823_Amanda_Vichy_15_3.raw \| 41619 \| 60305 \| 187.42 \| 145.00 \| \| 280823_Amanda_Vichy_15_4.raw \| 42883 \| 55943 \| 181.89 \| 145.00 \| \| 280823_Amanda_Vichy_17_1.raw \| 29867 \| 101569 \| 261.16 \| 145.00 \| \| 280823_Amanda_Vichy_17_2.raw \| 32623 \| 91796 \| 239.10 \| 145.00 \| \| 280823_Amanda_Vichy_19_1.raw \| 32187 \| 93754 \| 242.34 \| 145.00 \| \| 280823_Amanda_Vichy_19_2.raw \| 36232 \| 79410 \| 215.28 \| 145.00 \| \| 280823_Amanda_Vichy_21_1.raw \| 29675 \| 102486 \| 262.84 \| 145.00 \| \| 280823_Amanda_Vichy_21_2.raw \| 29973 \| 101458 \| 260.24 \| 145.00 \| \| 280823_Amanda_Vichy_23_2.raw \| 32606 \| 92679 \| 239.23 \| 145.00 \| \| 280823_Amanda_Vichy_23_3.raw \| 32513 \| 92365 \| 239.91 \| 145.00 \| \| 280823_Amanda_Vichy_29_1.raw \| 28085 \| 108582 \| 277.69 \| 144.98 \| \| 280823_Amanda_Vichy_29_2.raw \| 31171 \| 97602 \| 250.18 \| 144.97 \| \| 280823_Amanda_Vichy_31_1.raw \| 41463 \| 60813 \| 188.12 \| 145.00 \| \| 280823_Amanda_Vichy_31_2.raw \| 34251 \| 86354 \| 227.73 \| 145.00 \| \| 280823_Amanda_Vichy_33_1.raw \| 51935 \| 23562 \| 150.19 \| 145.00 \| \| 280823_Amanda_Vichy_33_2.raw \| 51564 \| 24966 \| 151.27 \| 145.00 \| |  |  |  |  |
| --- | --- | --- | --- | --- | --- | --- | --- | --- | --- | --- | --- | --- | --- | --- | --- | --- | --- | --- | --- | --- | --- | --- | --- | --- | --- | --- | --- | --- | --- | --- | --- | --- | --- | --- | --- | --- | --- | --- | --- | --- | --- | --- | --- | --- | --- | --- | --- | --- | --- | --- | --- | --- | --- | --- | --- | --- | --- | --- | --- | --- | --- | --- | --- | --- | --- | --- | --- | --- | --- | --- | --- | --- | --- | --- | --- | --- | --- | --- | --- | --- | --- | --- | --- | --- | --- | --- | --- | --- | --- | --- | --- | --- | --- | --- | --- | --- | --- | --- | --- | --- | --- | --- | --- | --- | --- | --- | --- | --- | --- | --- | --- | --- | --- | --- | --- | --- | --- | --- | --- | --- | --- | --- | --- | --- | --- | --- | --- | --- | --- | --- | --- | --- | --- | --- | --- | --- | --- | --- | --- | --- | --- | --- | --- | --- | --- | --- | --- | --- | --- | --- | --- | --- | --- | --- | --- | --- | --- | --- | --- | --- | --- | --- | --- | --- | --- | --- | --- | --- | --- | --- | --- | --- | --- | --- | --- | --- | --- | --- | --- | --- | --- | --- | --- | --- | --- | --- | --- | --- | --- | --- | --- | --- | --- | --- | --- | --- | --- | --- | --- | --- | --- | --- | --- | --- | --- | --- | --- | --- | --- |

The modules from RawVegetable used to analyse the data were the full and charged chromatograms, TopN density estimation, precursor signal ratio and Xrea score.

- **Chromatography analysis**

This module consists of graphically comparing the chromatograms of different samples mostly by checking whether they overlap for most of the run and if the signal intensity matches. Individually, it is also interesting to look at how well defined the peaks are, which might be indicative of a good separation of the molecules.

RawVegetable also permits deconvoluting MS spectra in order to build a chromatogram specifically of individual charges. While this is most interesting when trying to optimize the chromatography for highly charged species (such as crosslinked peptides), in the case of linear proteomics, it is also appropriate to check it as an indication of successful digestion of the proteins, as it will result in most of the peptides having charges 2+ and 3+.

In our experiments, every sample showed great match between the chromatograms of the technical replicates and charged chromatograms presented an abundance of 2+ and 3+ charged species.

- **TopN density estimation**

This analysis consists of applying a KDE (Kernel Density Estimation) on the number of MS/MS scans per MS scan, which creates a smoothed curve of these values. This allows us to analyse how efficient the MS/MS acquisition is. A satisfactory experiment usually shows a consistently high number of MS/MS events for most of the chromatography. This sort of plot can help the identification of points where the chromatography gradient could be adjusted, either due to under or over-sampling.

For our samples, in every run the TopN density was very consistent for most of the chromatography and also greatly matched between technical replicates.

- **Precursor signal ratio**

This feature provides a histogram with the distribution of ratios of the intensity of the precursor ion by the total signal of the MS/MS spectrum. For a satisfactory run, it is expected that most of the spectra show a low precursor signal, indicating an efficient peptide fragmentation and that an appropriate collisional energy was used.

The MS/MS spectra generated in this dataset had, for the most part, precursor signal ratio below 2.5% and also greatly matched between technical replicates.

- **Xrea Score**

The calculated Xrea is a score used to analyse the quality of a spectrum. Briefly, it is calculated according to the heterogeneity of the peaks' intensities in a spectrum. Low quality spectra tend to have many peaks of similar intensity, while a spectrum from a nicely fragmented peptide usually has a more diverse distribution. This leads to score that ranges between 0 and 1, where higher scores indicate a spectrum of higher quality. In RawVegetable, this is shown as a plot of Xrea over retention time, which is then smoothed to form a trendline, that can be used to assess areas where the better spectra are being generated.

The trendlines of the Xreas of the MS/MS scans of all runs showed a higher quality of spectrum being generated by the 15 to 135 minutes of the run.

The following pages show the images generated from all these assessments paired by their technical replicates. Each section contains the images for skin’s face analysed in the following order:

- **Full chromatogram**: shows the chromatography of both technical replicates in different colours as base peak mode.
- **Charged chromatograms**: for each different technical replicate, there is an image depicting the full chromatogram, and the chromatograms of charges 2+ and 3+, which are indicated in the plot legend.
- **Precursor signal ratio**: one plot with the ratio of both technical replicates in different colours.
- **TopN density estimation**: one plot showing the TopN density of both technical replicates in different colours.
- **Xrea score**: one plot showing the Xrea of the MS/MS scans throughout the whole chromatography for both technical replicates in a lighter colour and the trendline in a darker colour. Each technical replicate is in a different colour.

In all, the samples used for this study passed our quality control and did not raise any major issues, which makes this dataset satisfactory for further analysis.

**References**

1. Kurt LU, Clasen MA, Santos MDM, Souza TACB, Andreassa EC, Lyra EB, et al. RawVegetable - A data assessment tool for proteomics and cross-linking mass spectrometry experiments. J Proteomics. 15 de agosto de 2020;225:103864.

1. **LEFT**
   - **08**

**
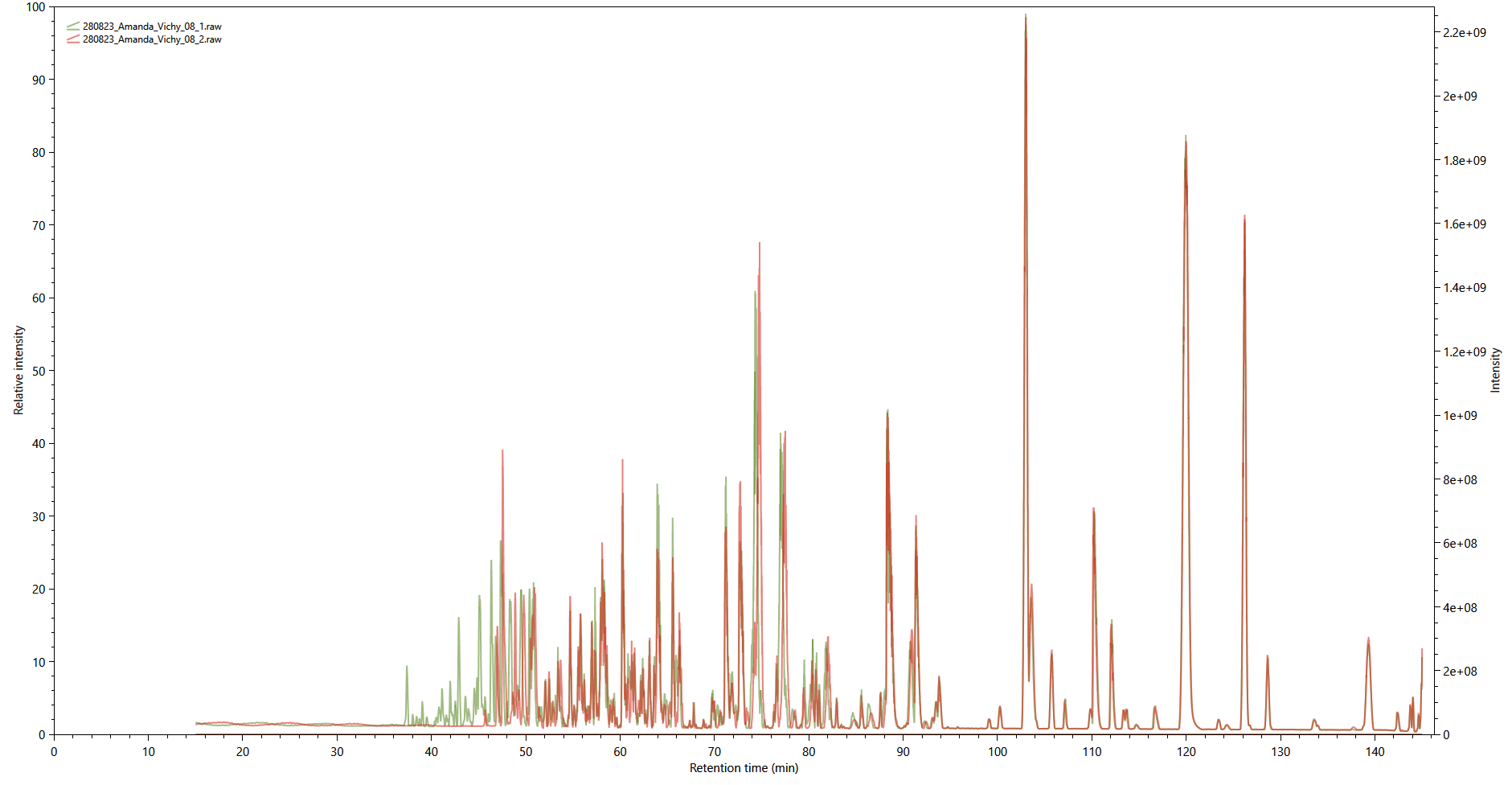
**
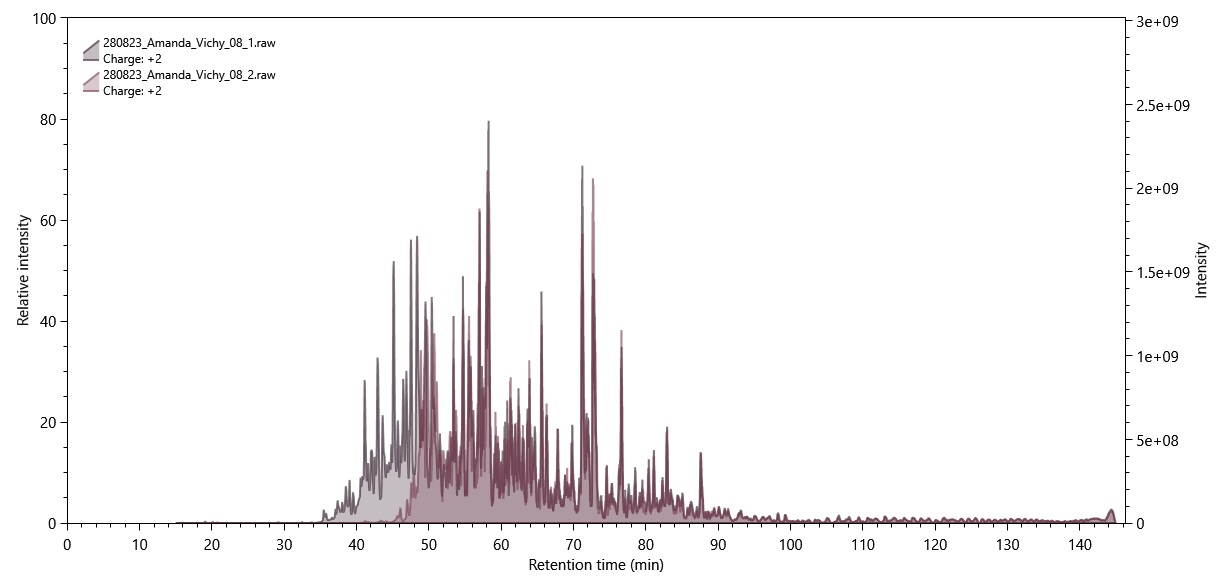


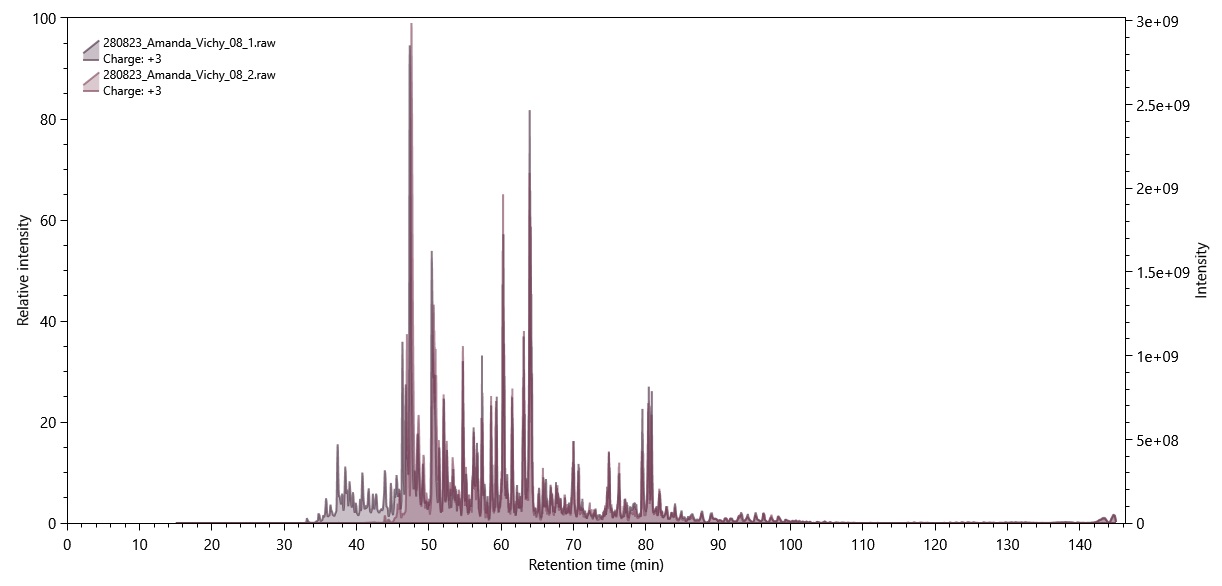

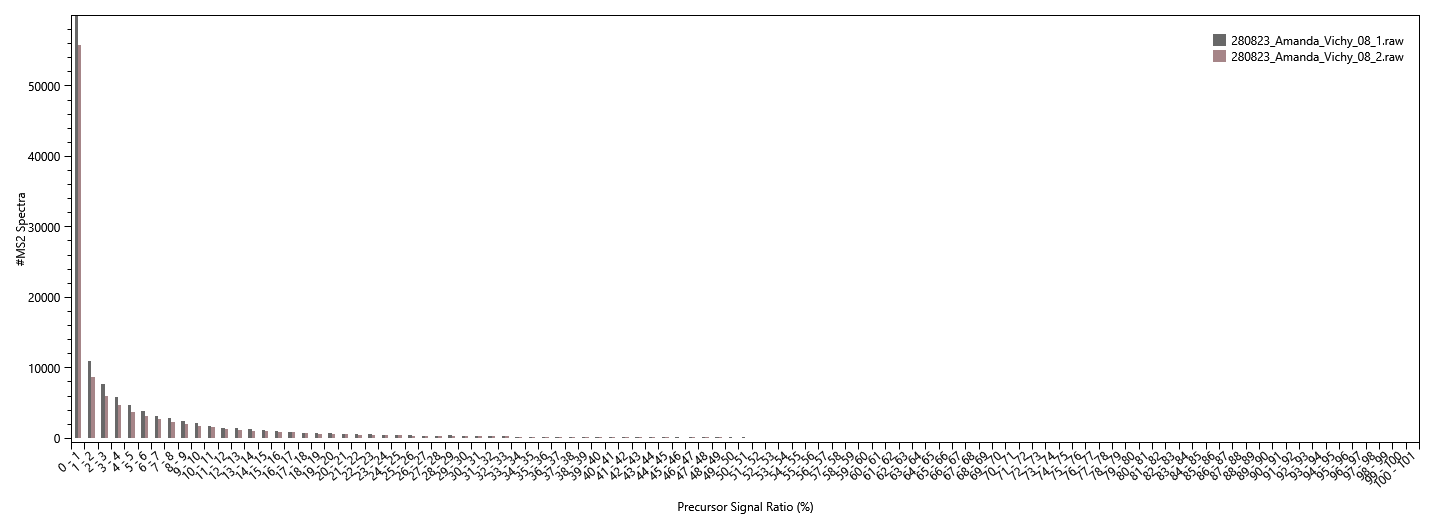


**
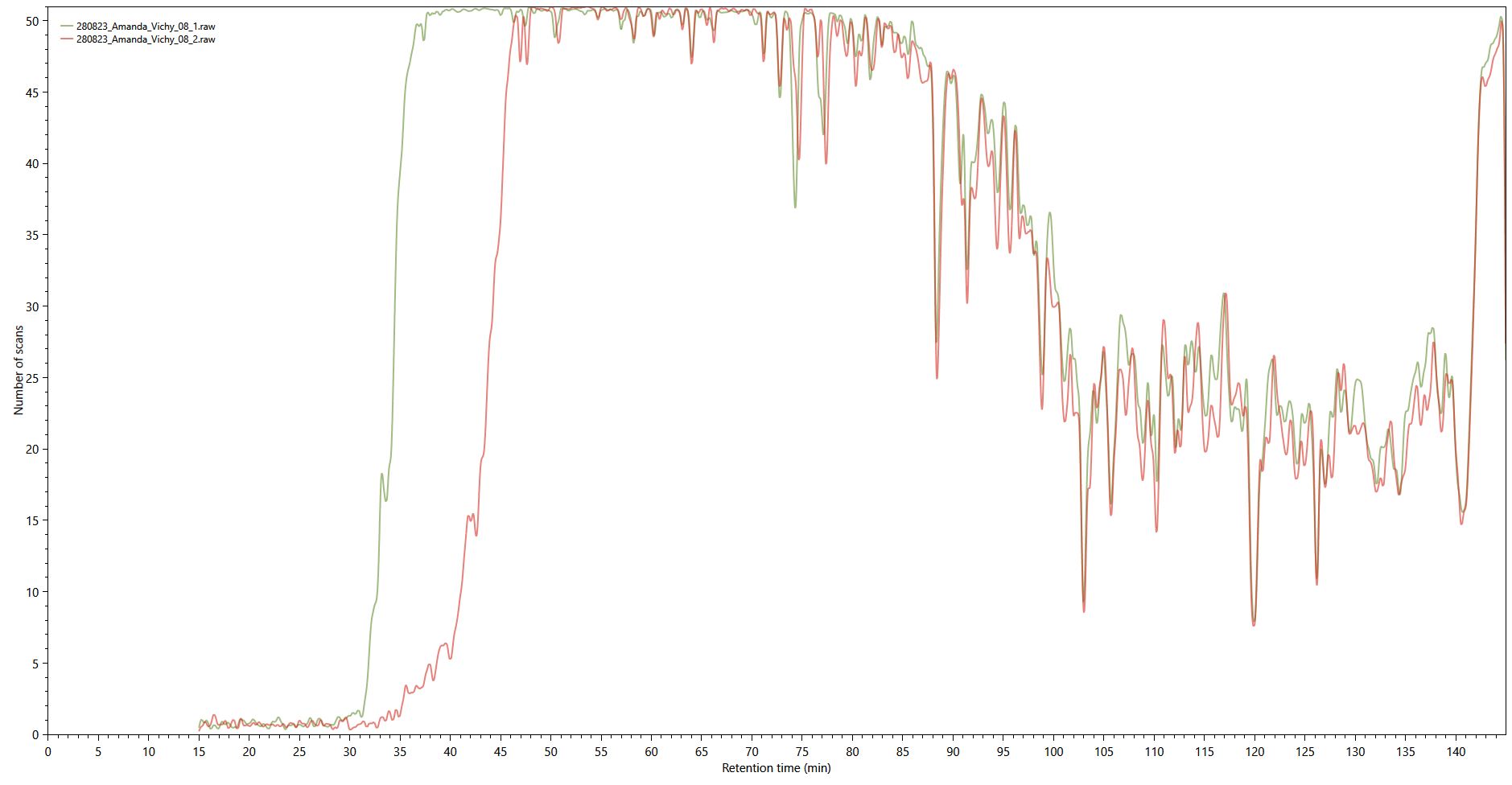

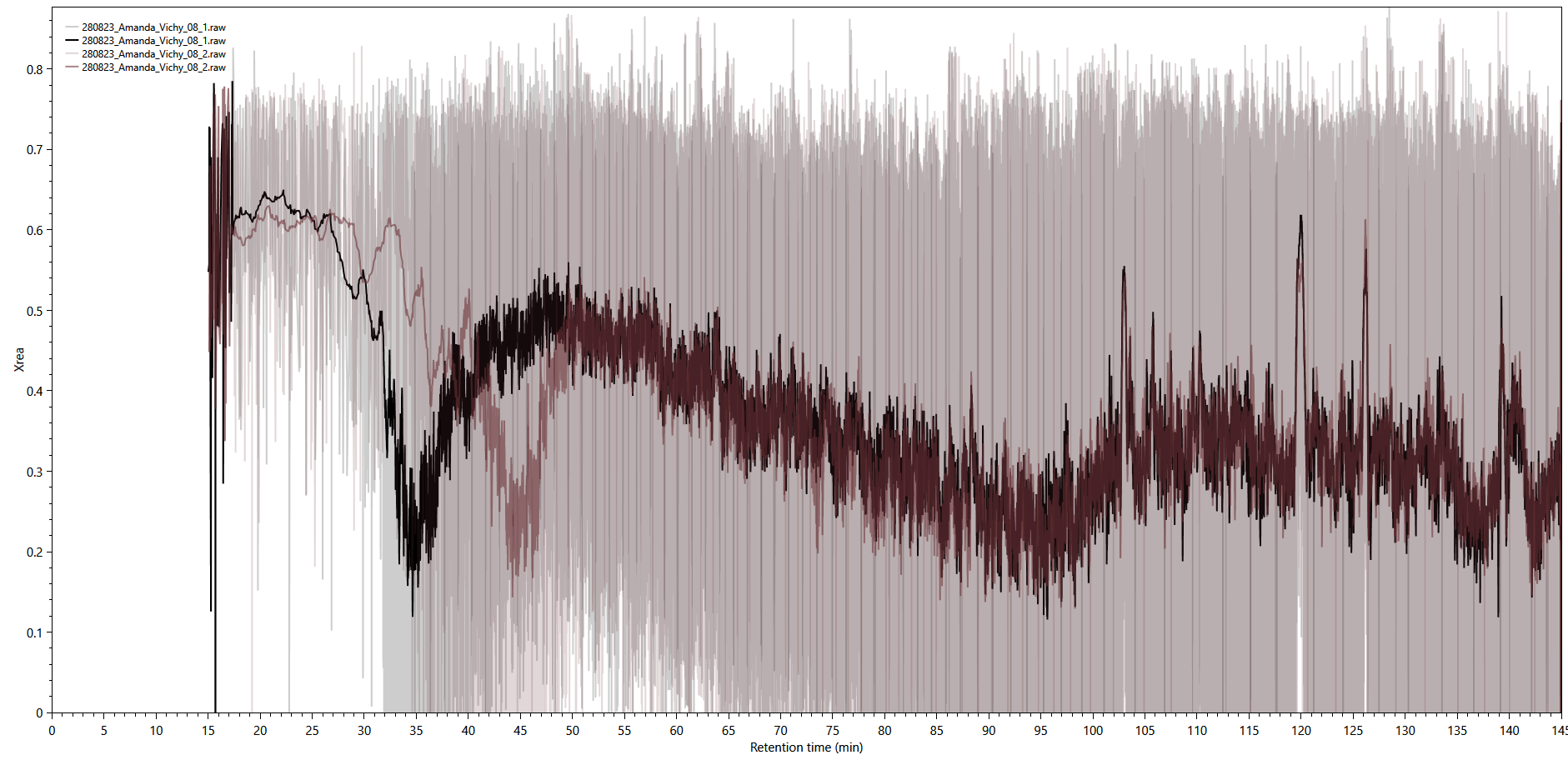
**

- - **10**

**
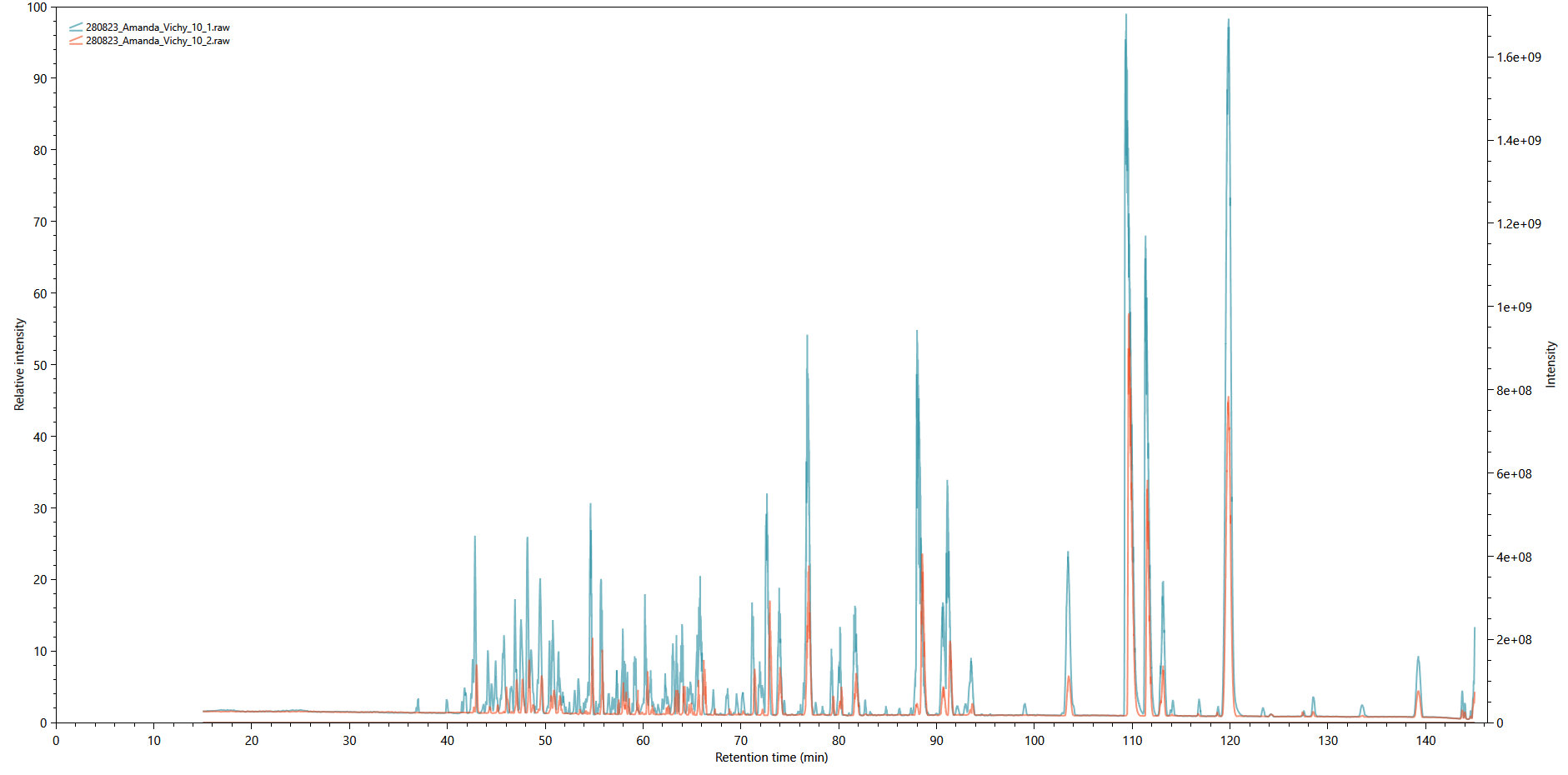
**
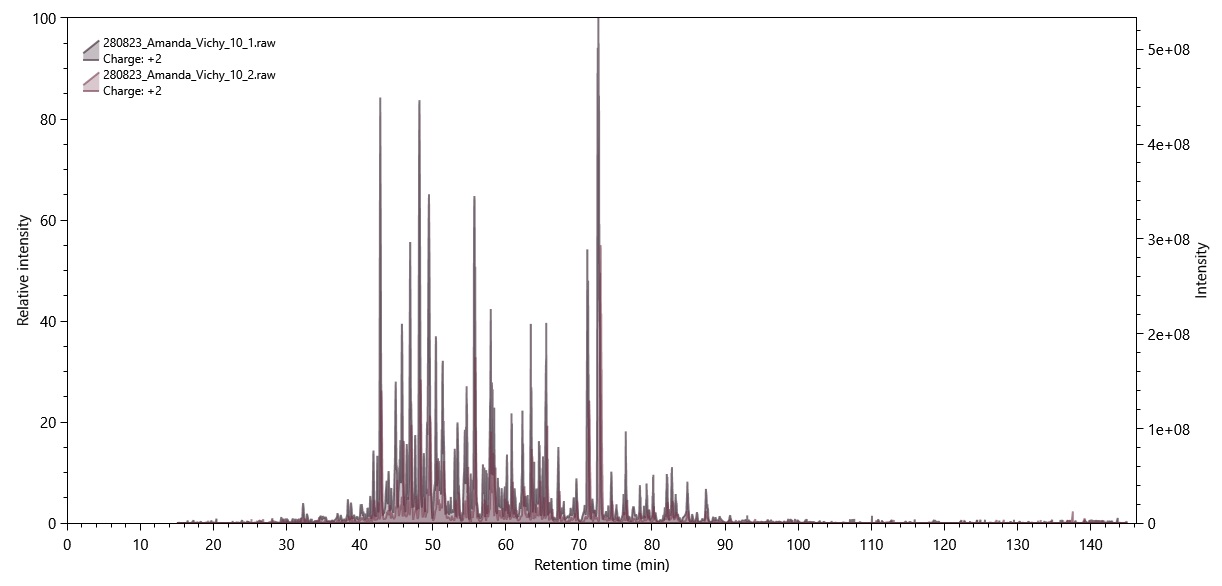


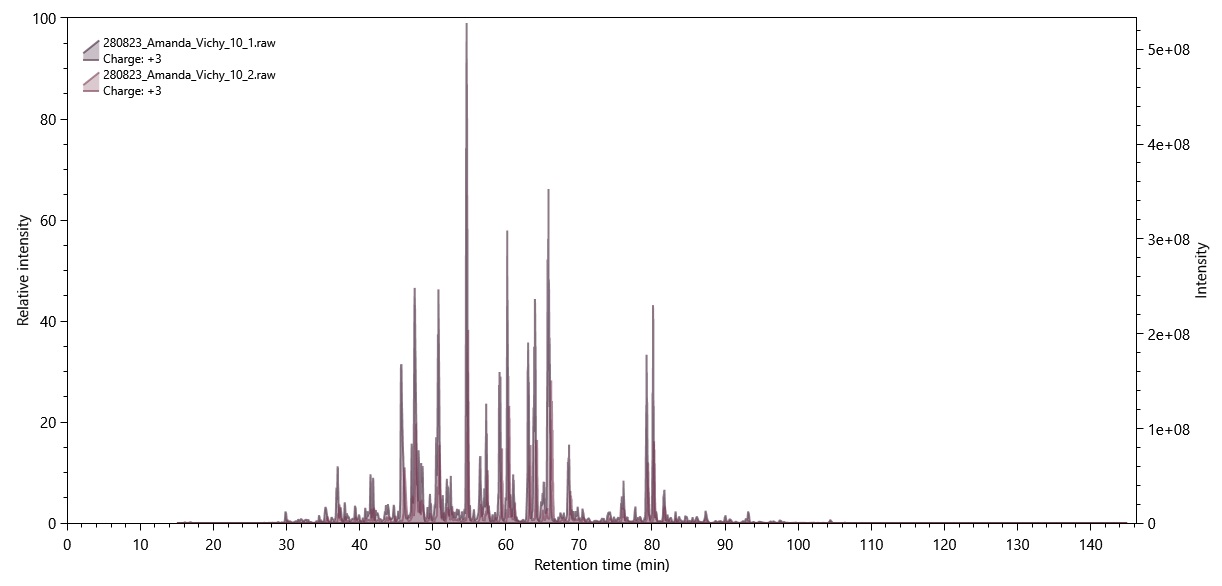
**
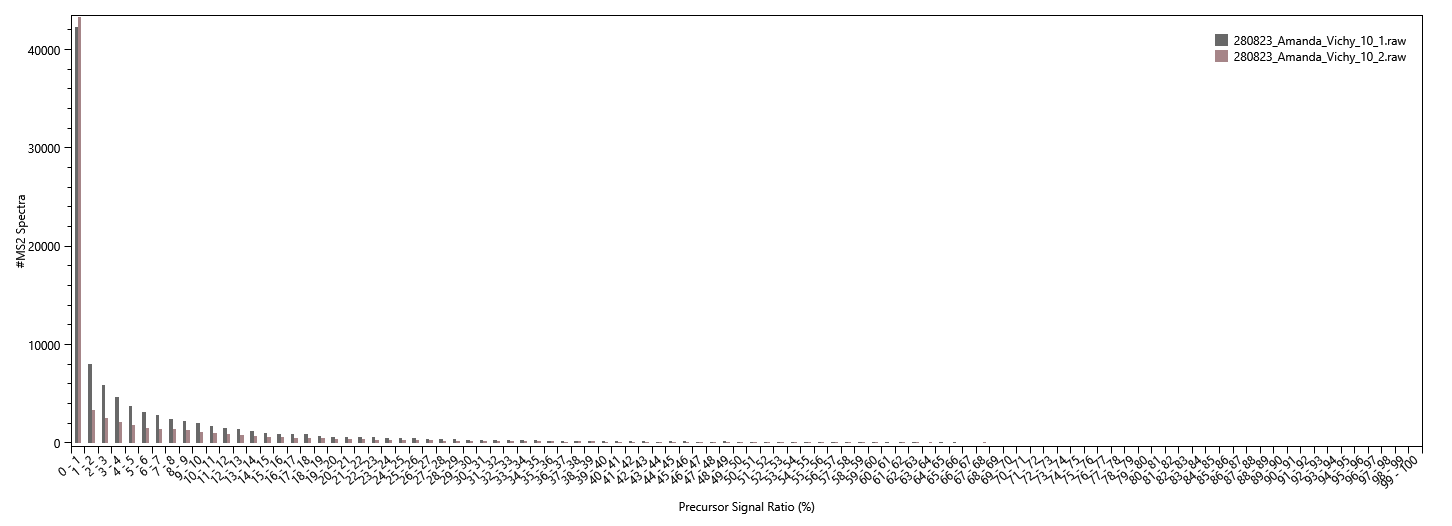

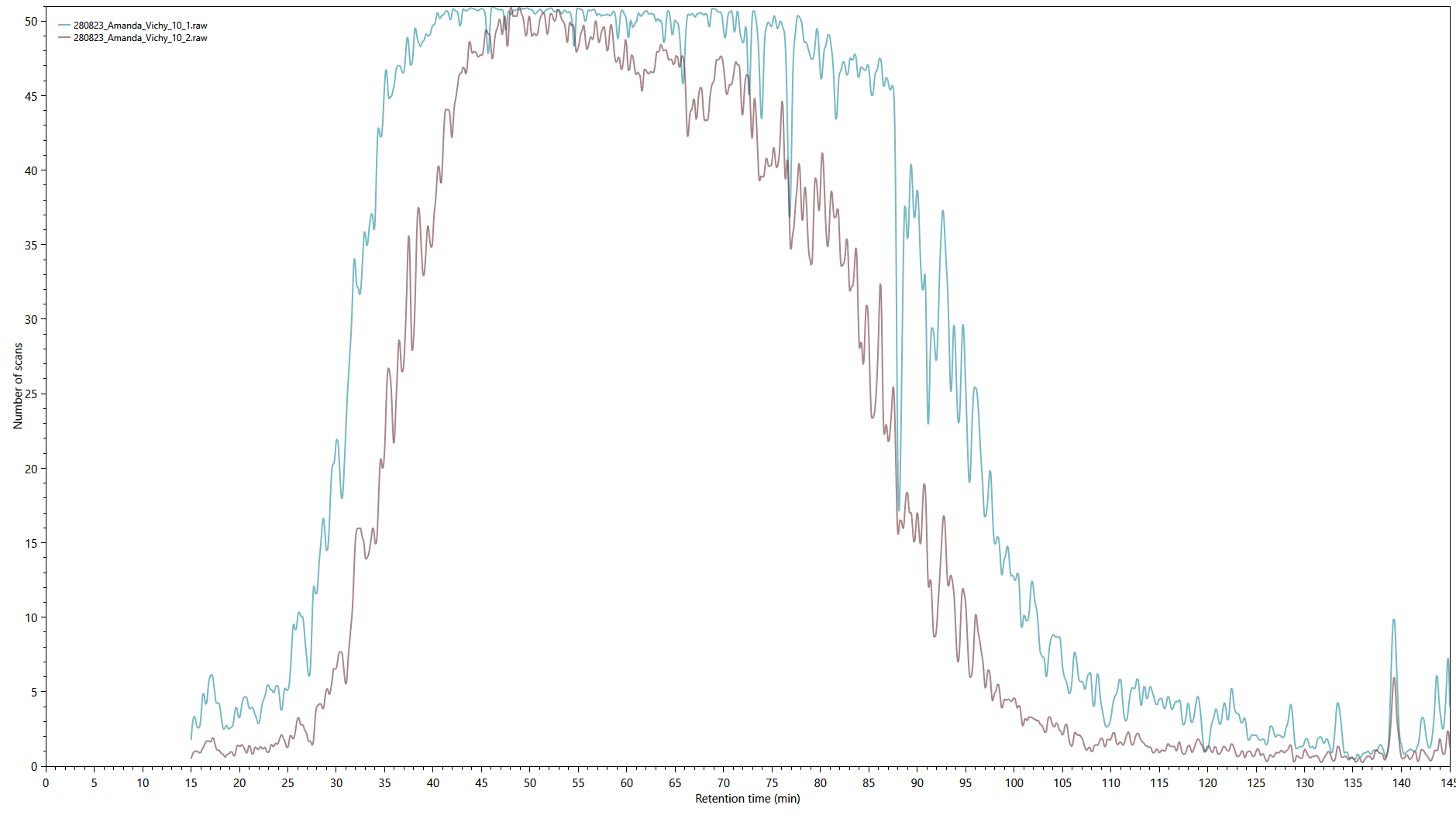

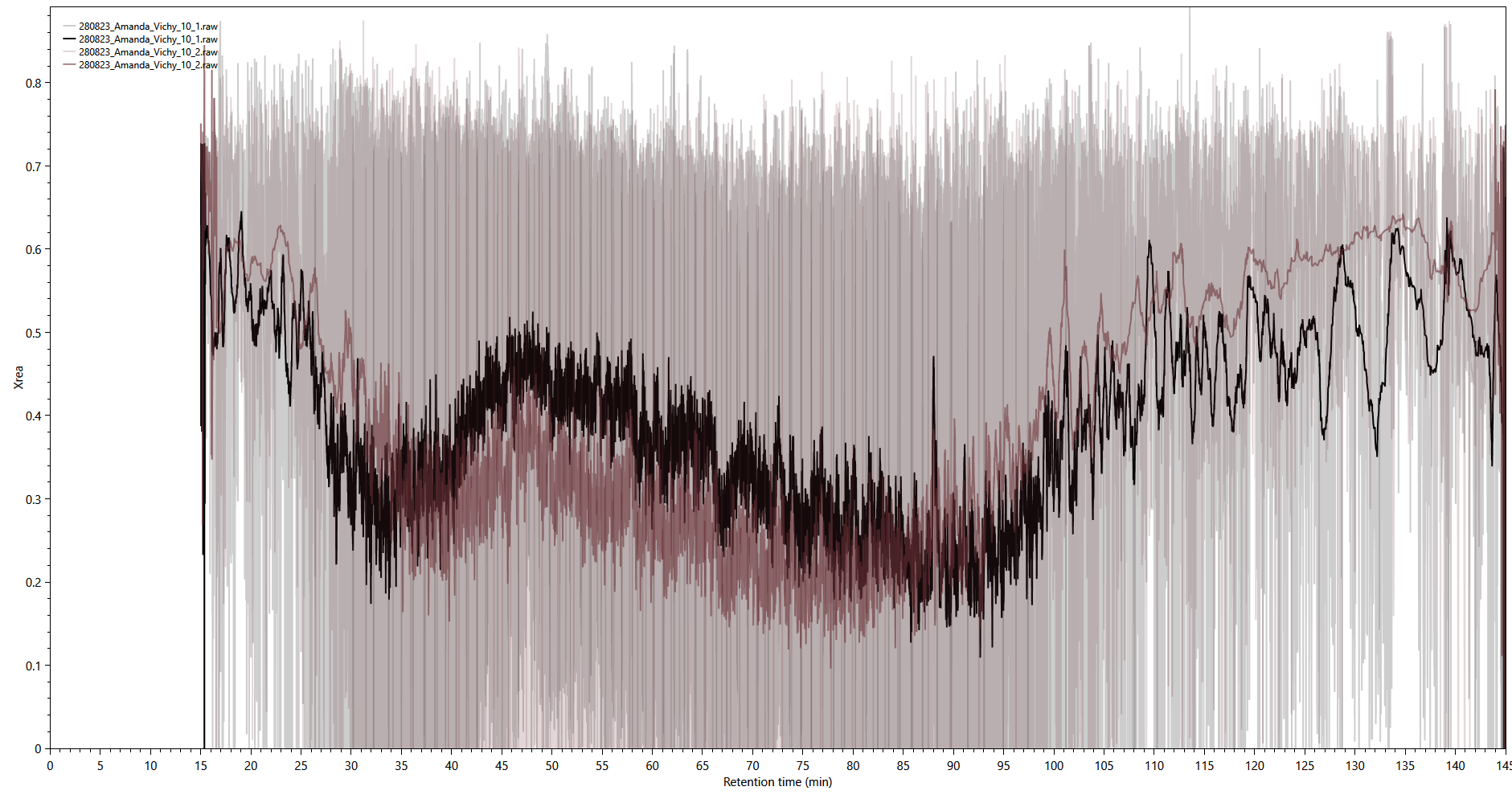
**

- - **16**


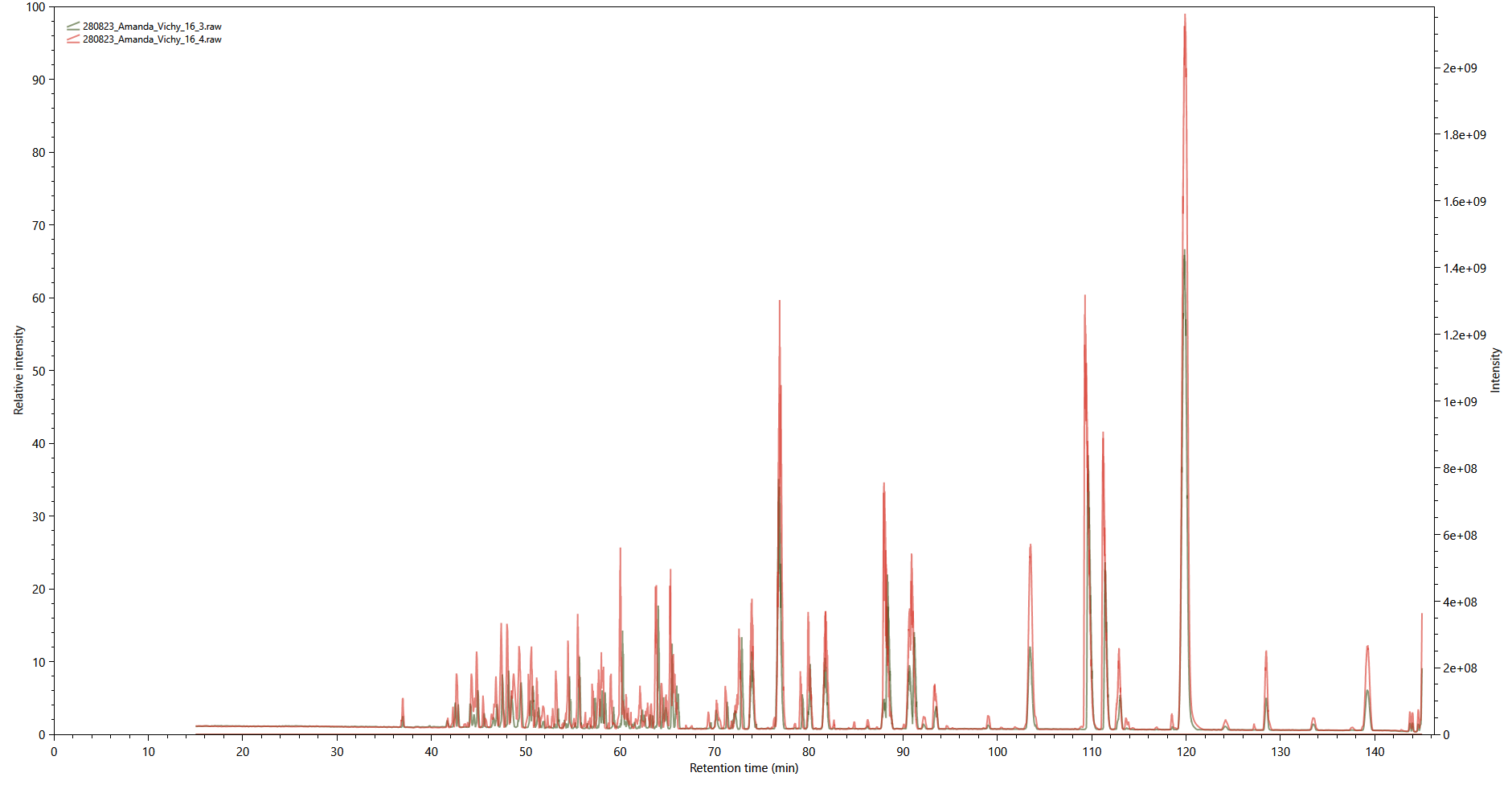


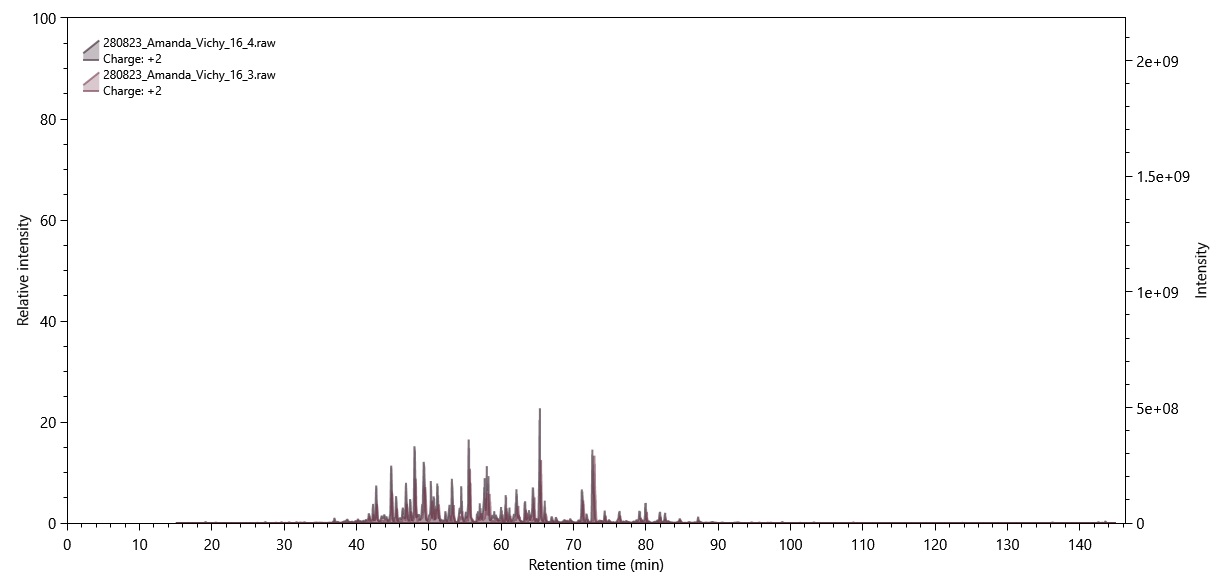


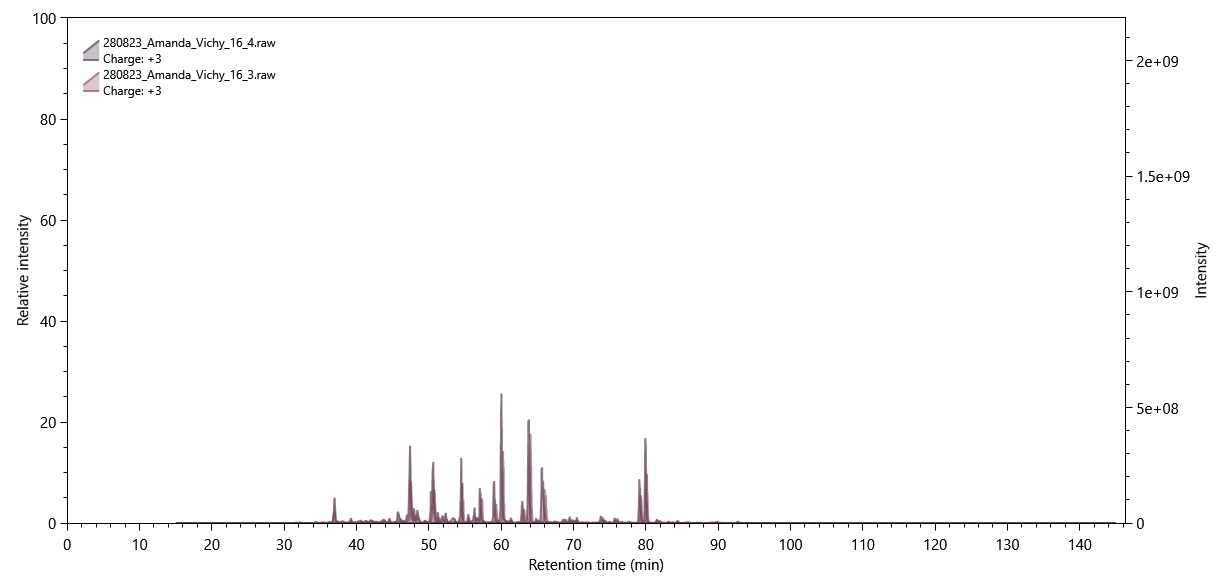

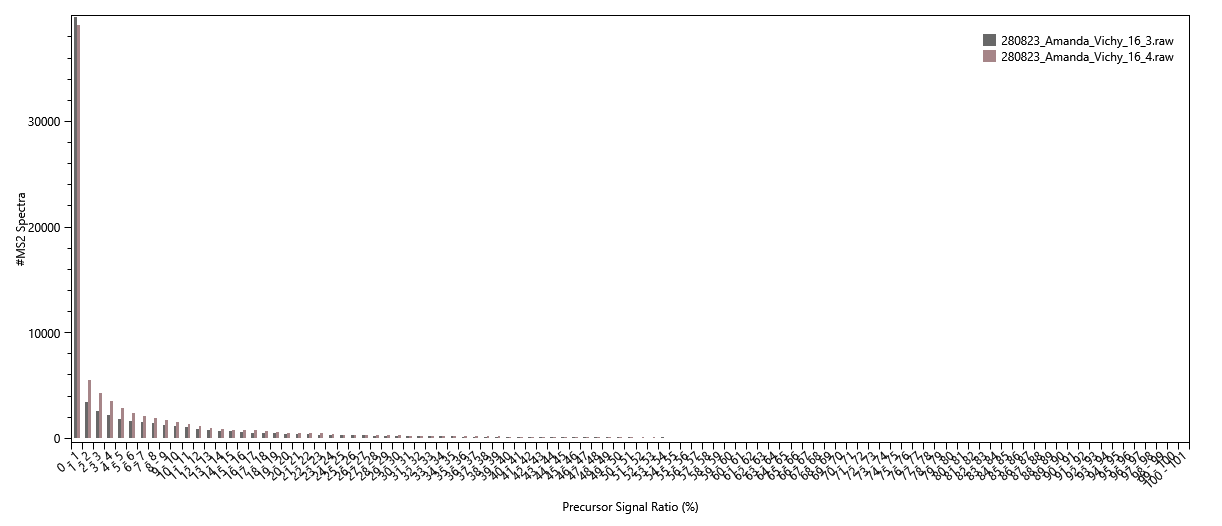

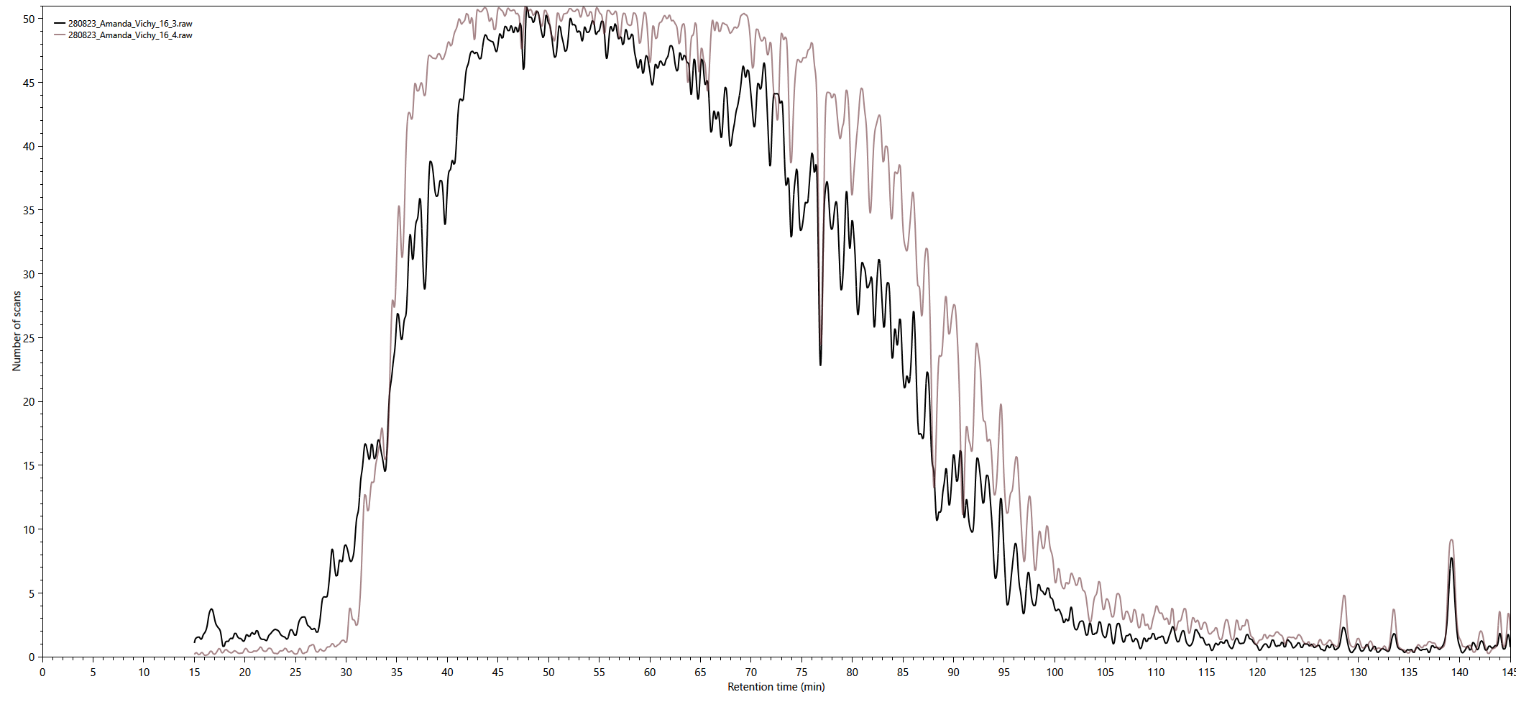

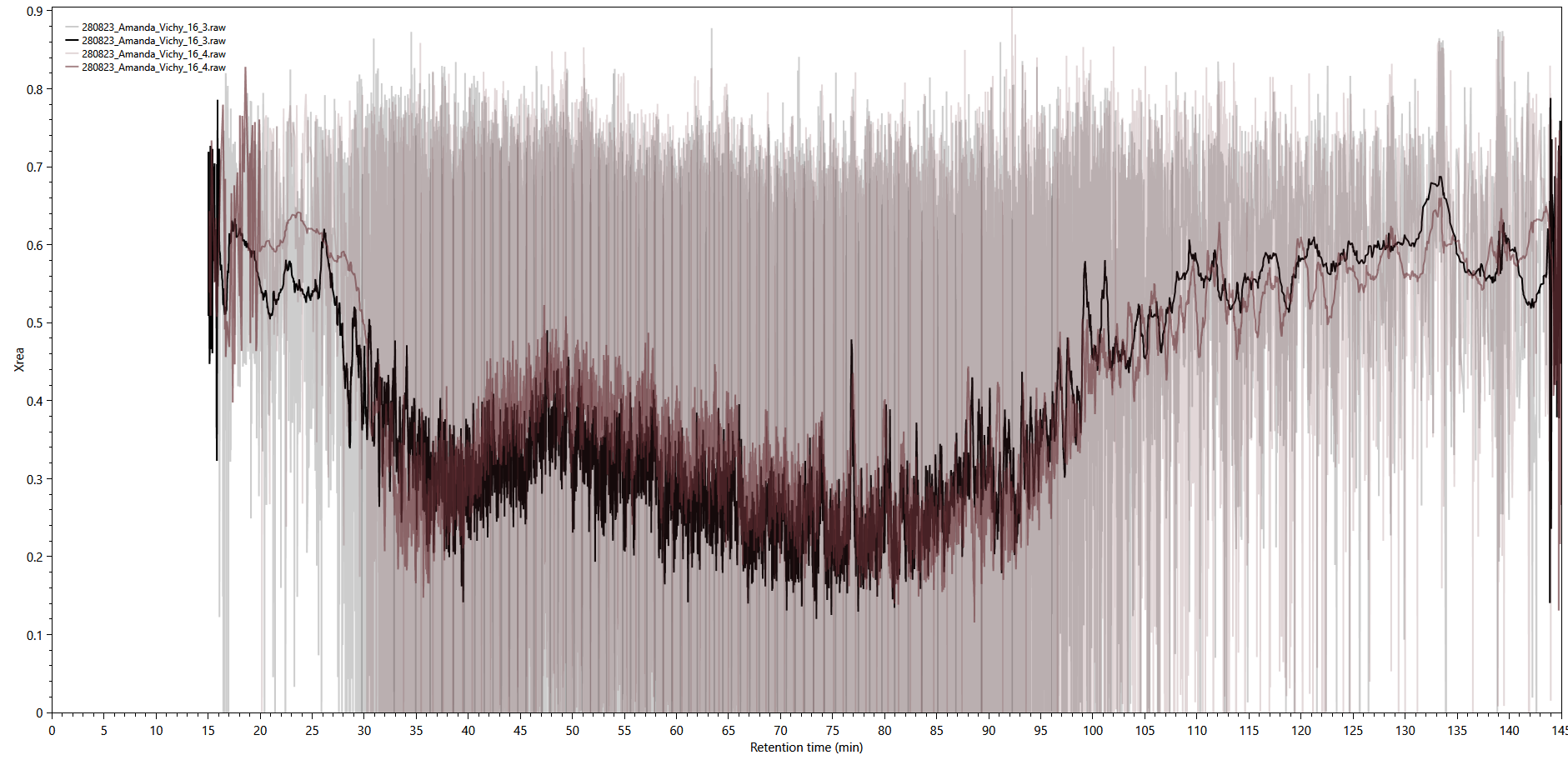


- **18**


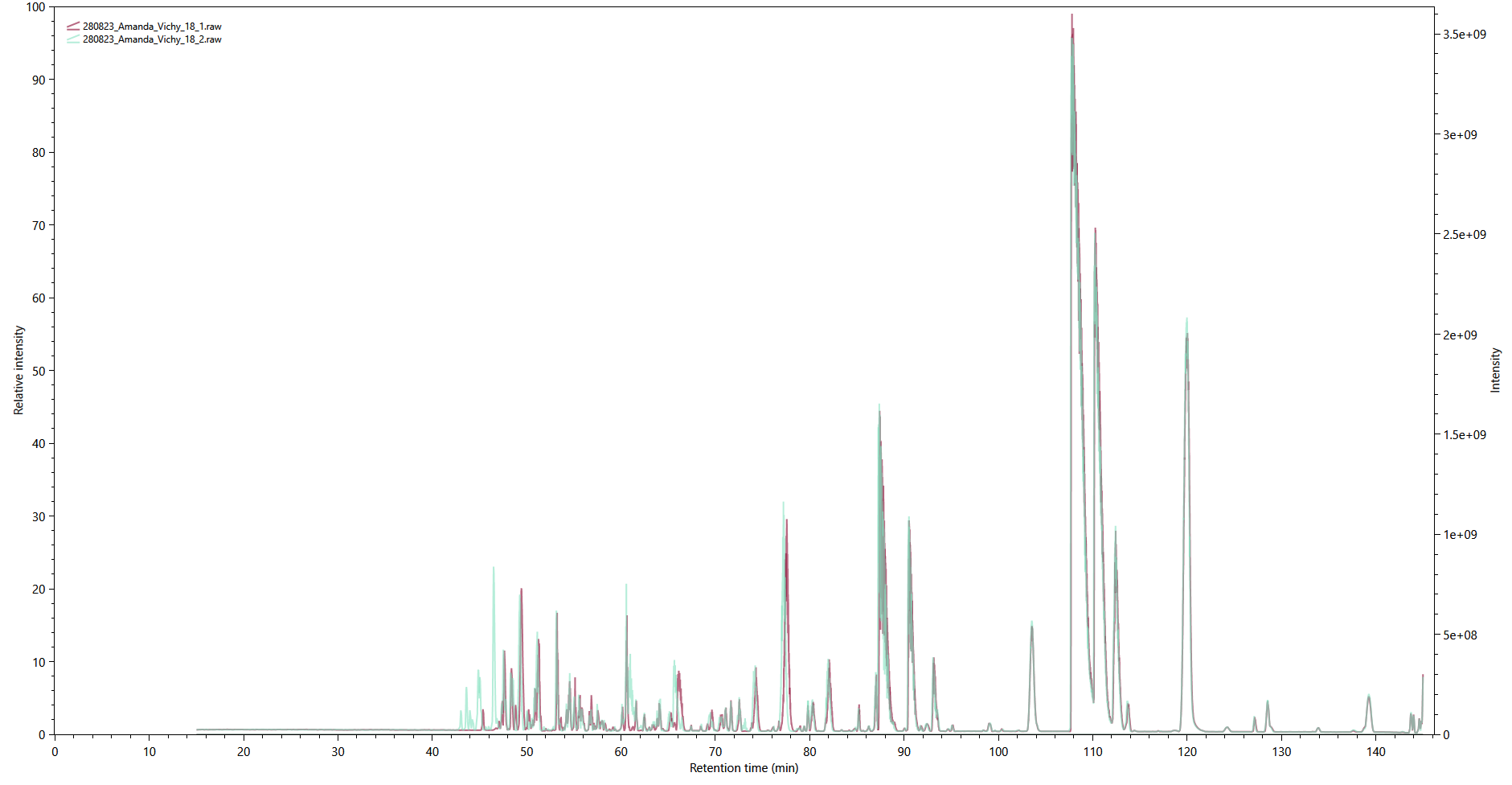

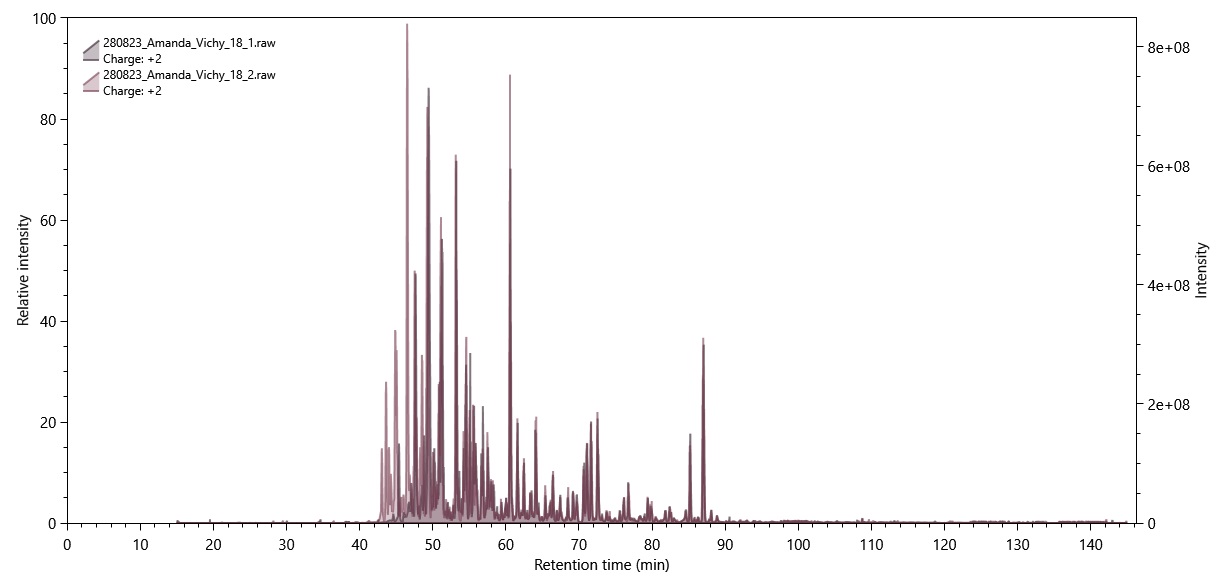

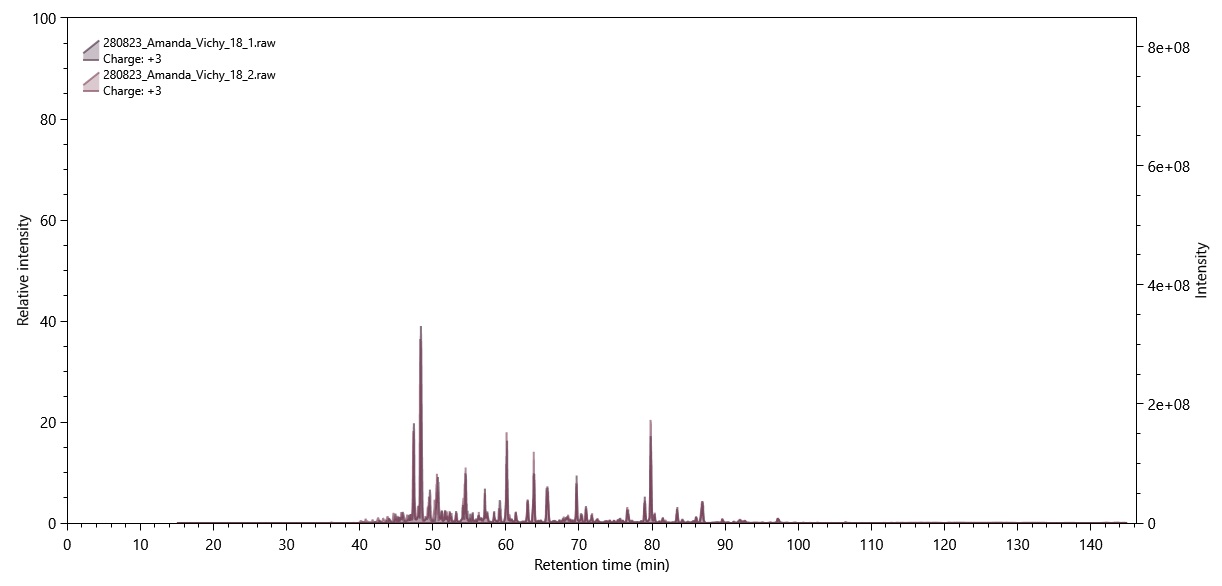

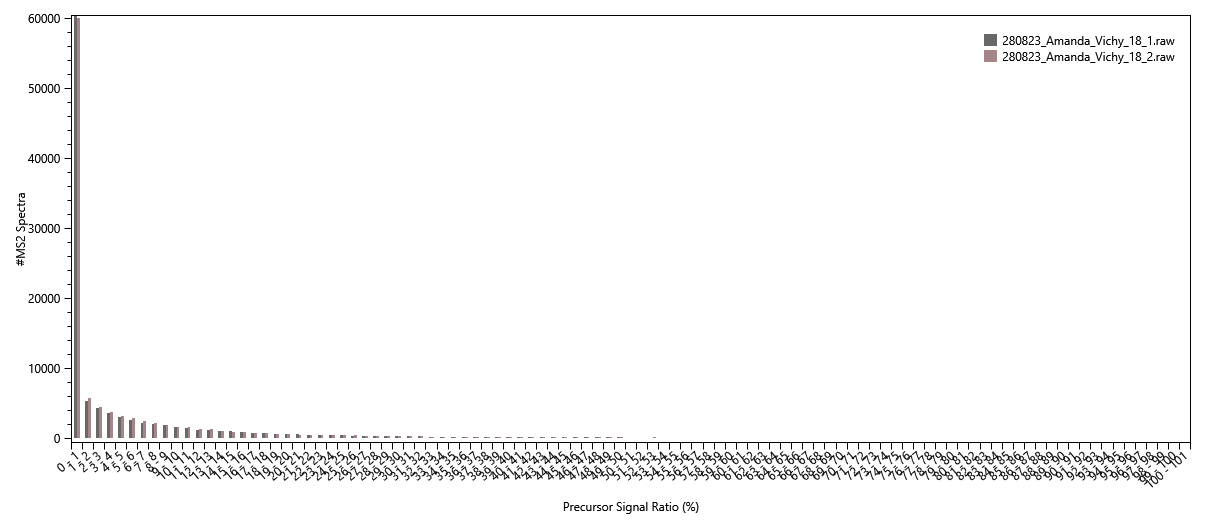


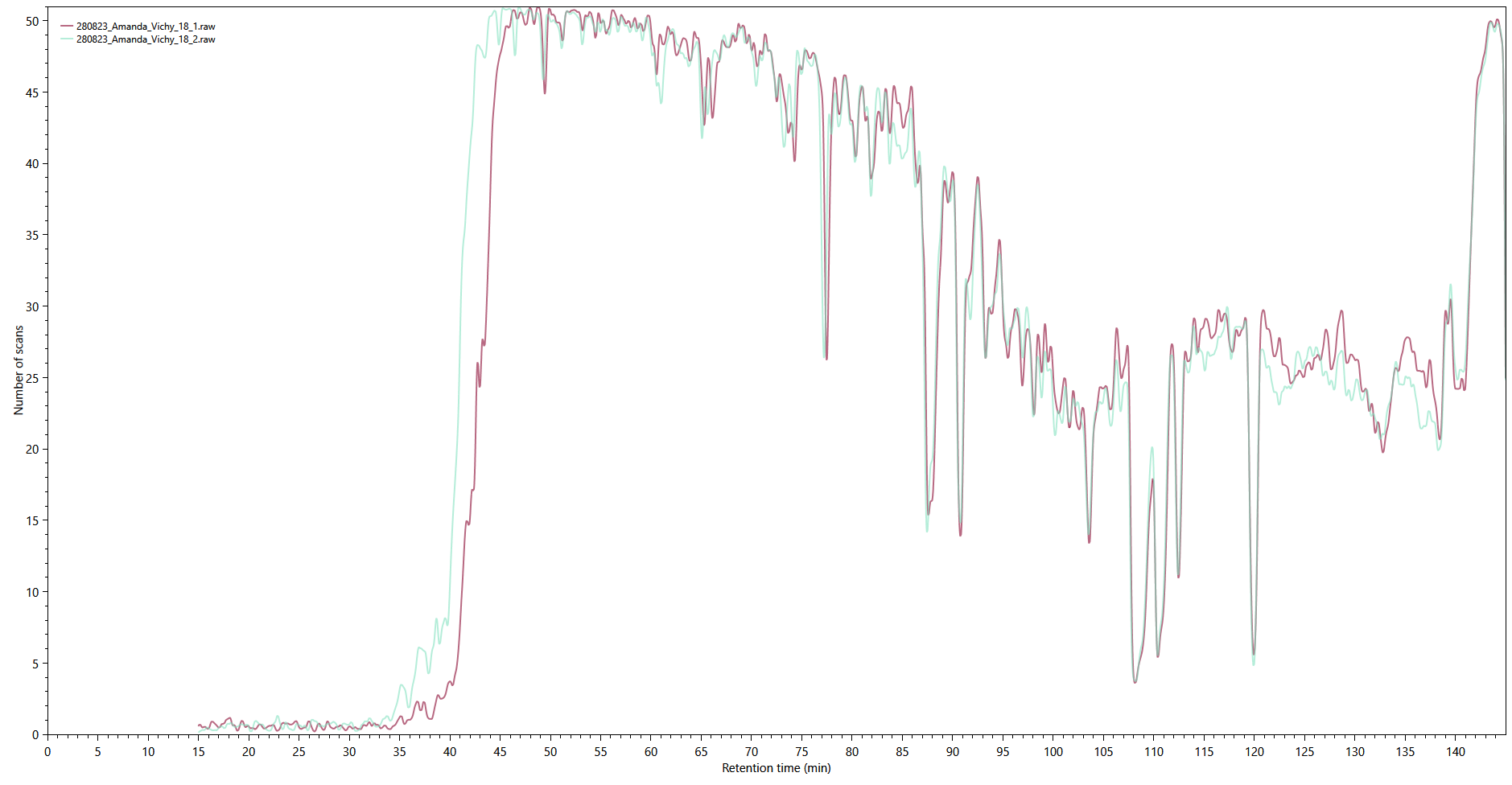

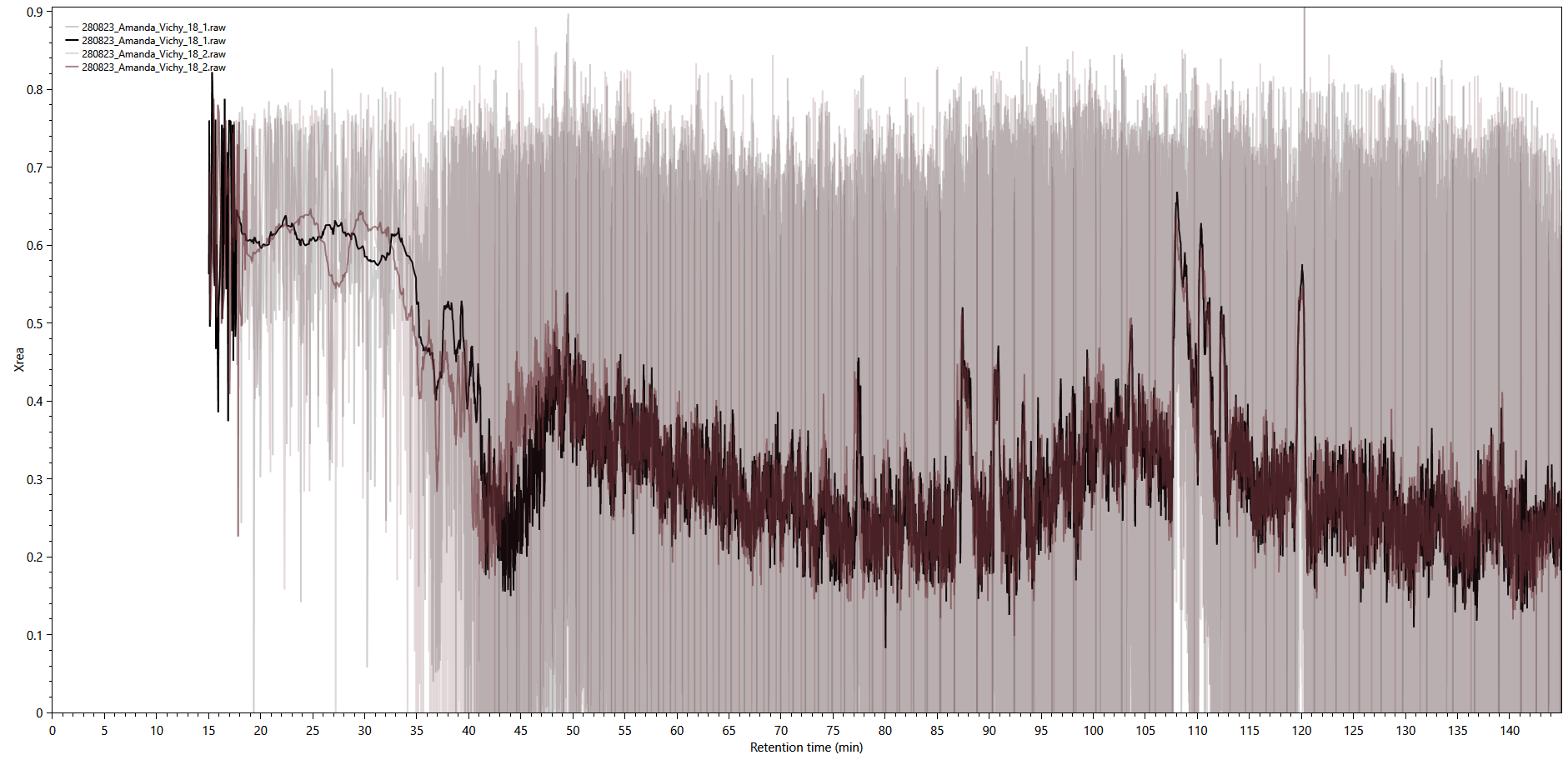


- **20**


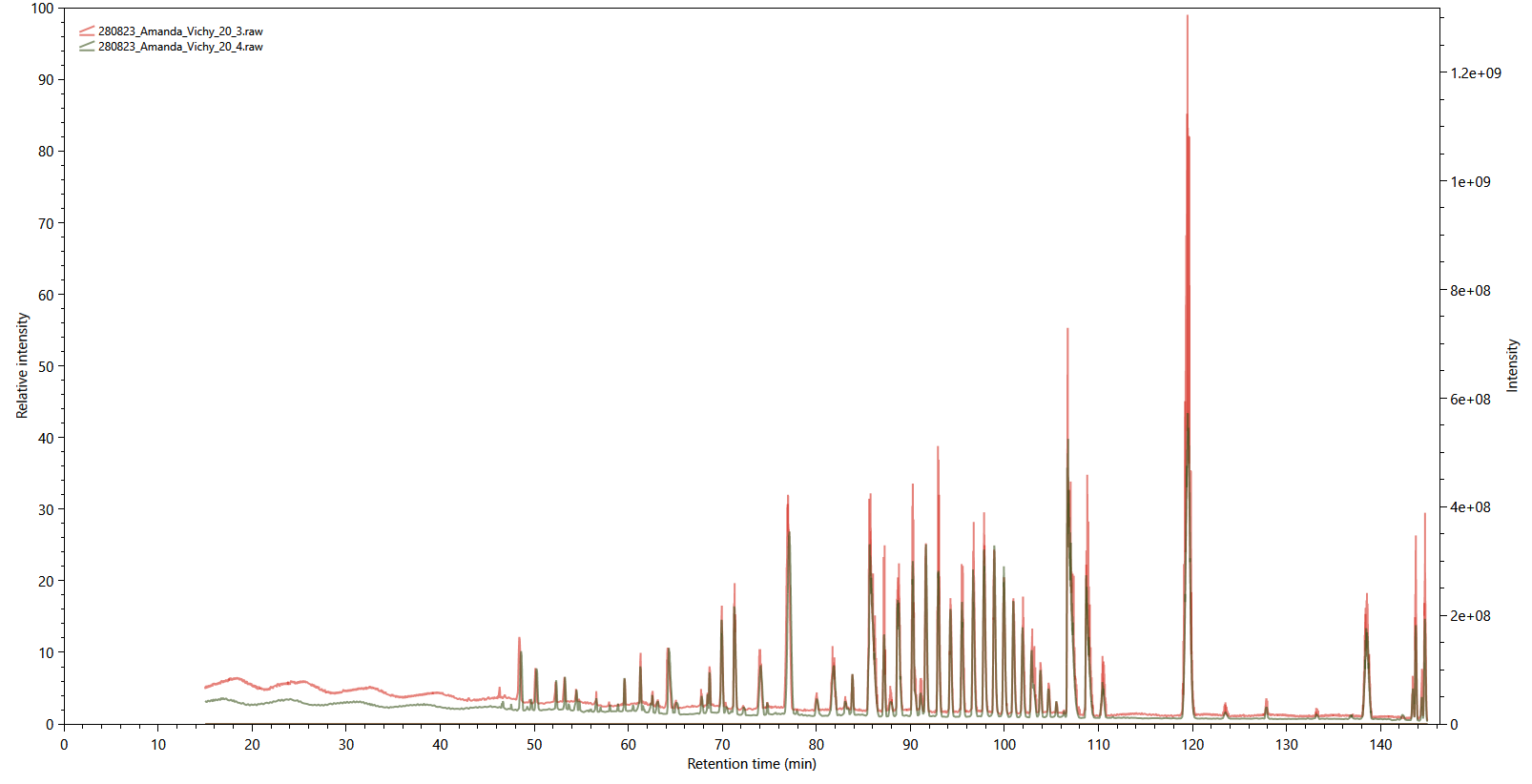


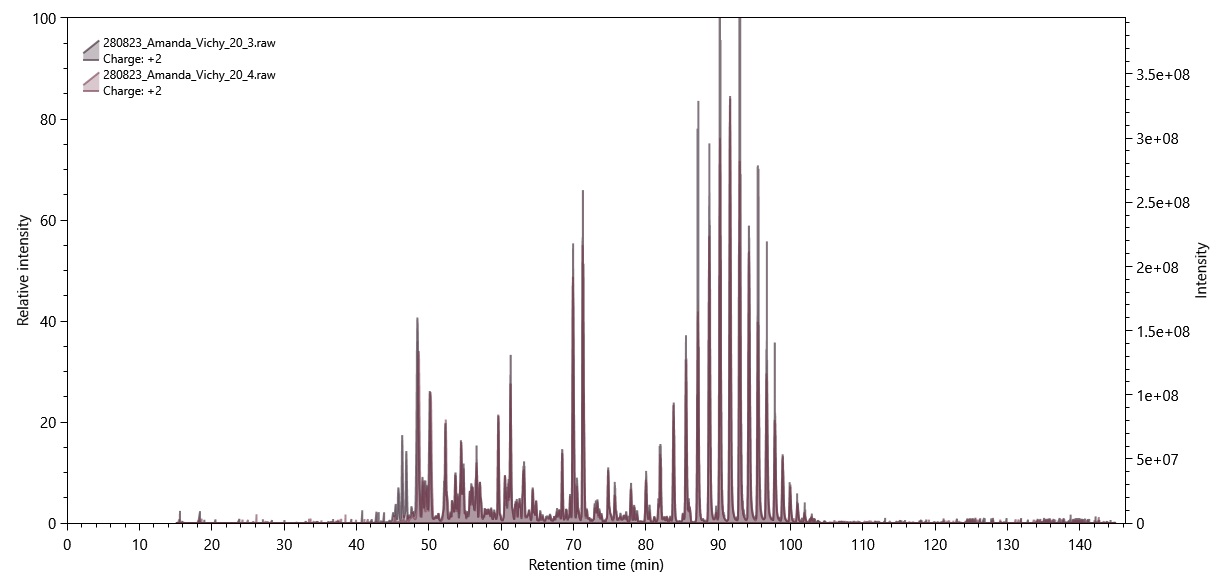


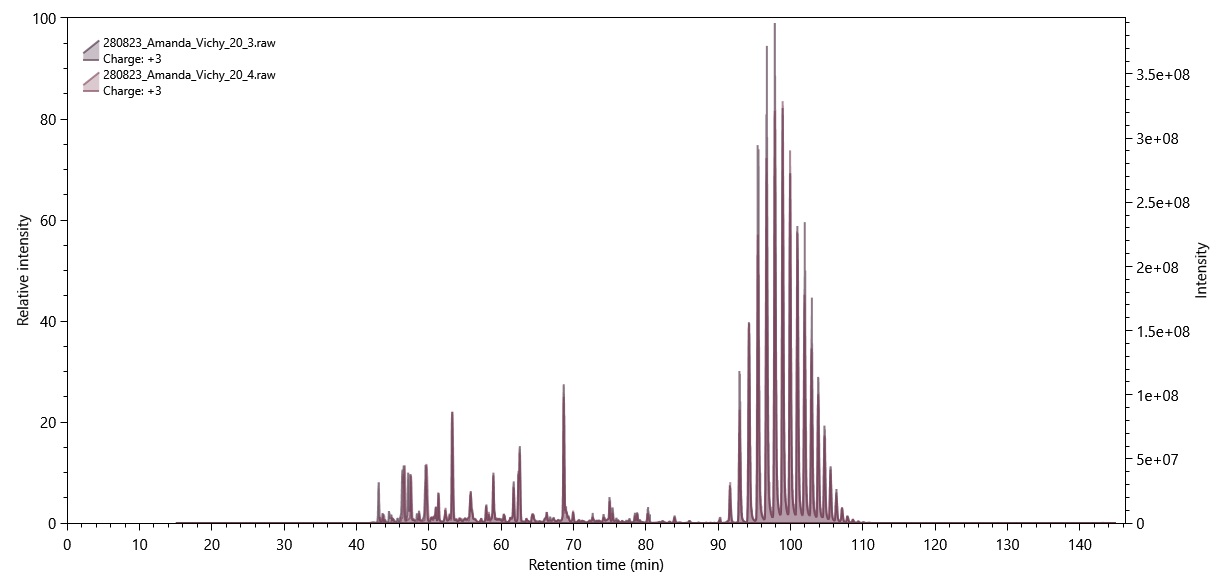

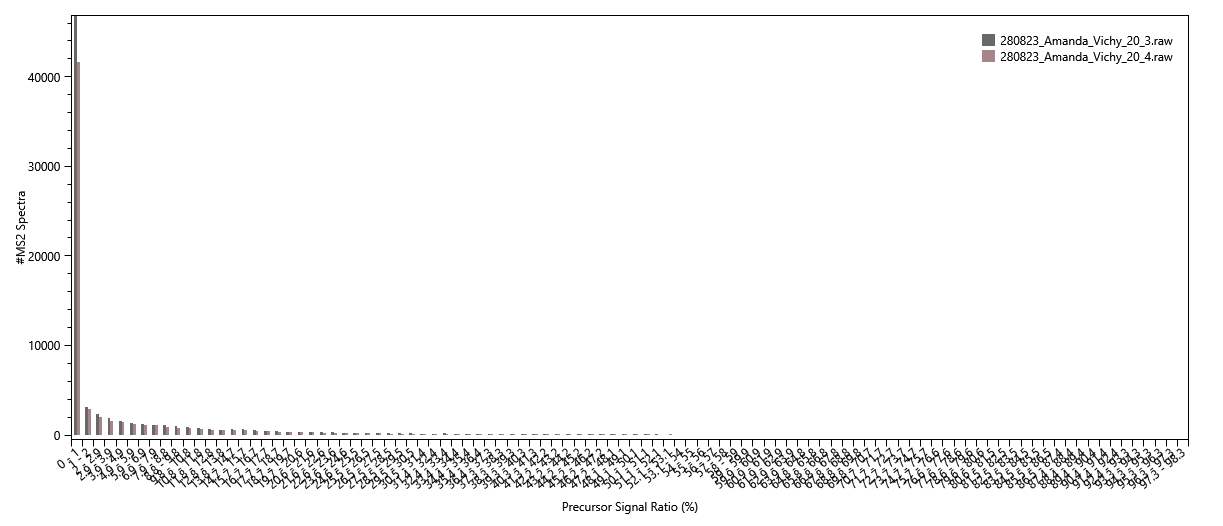

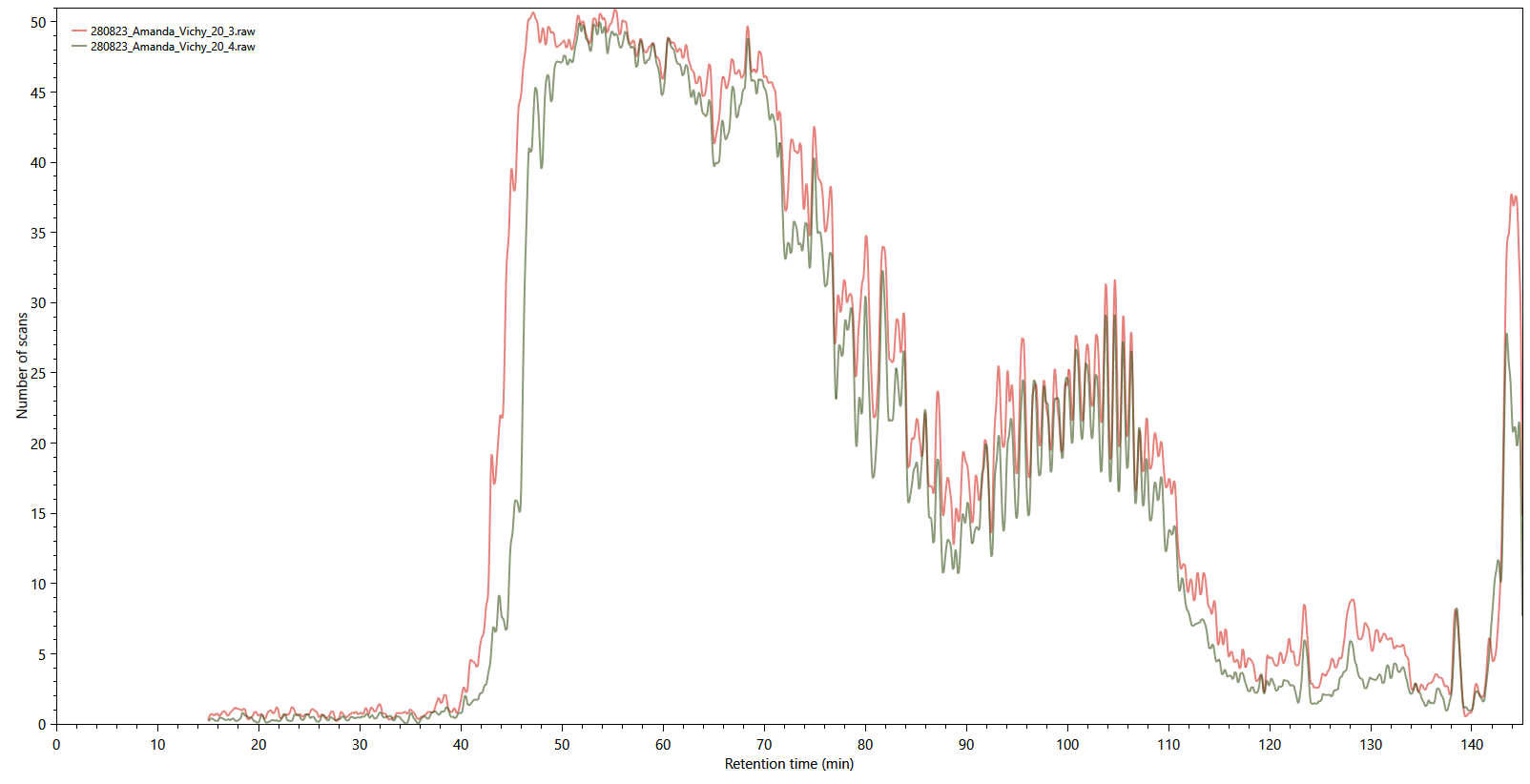

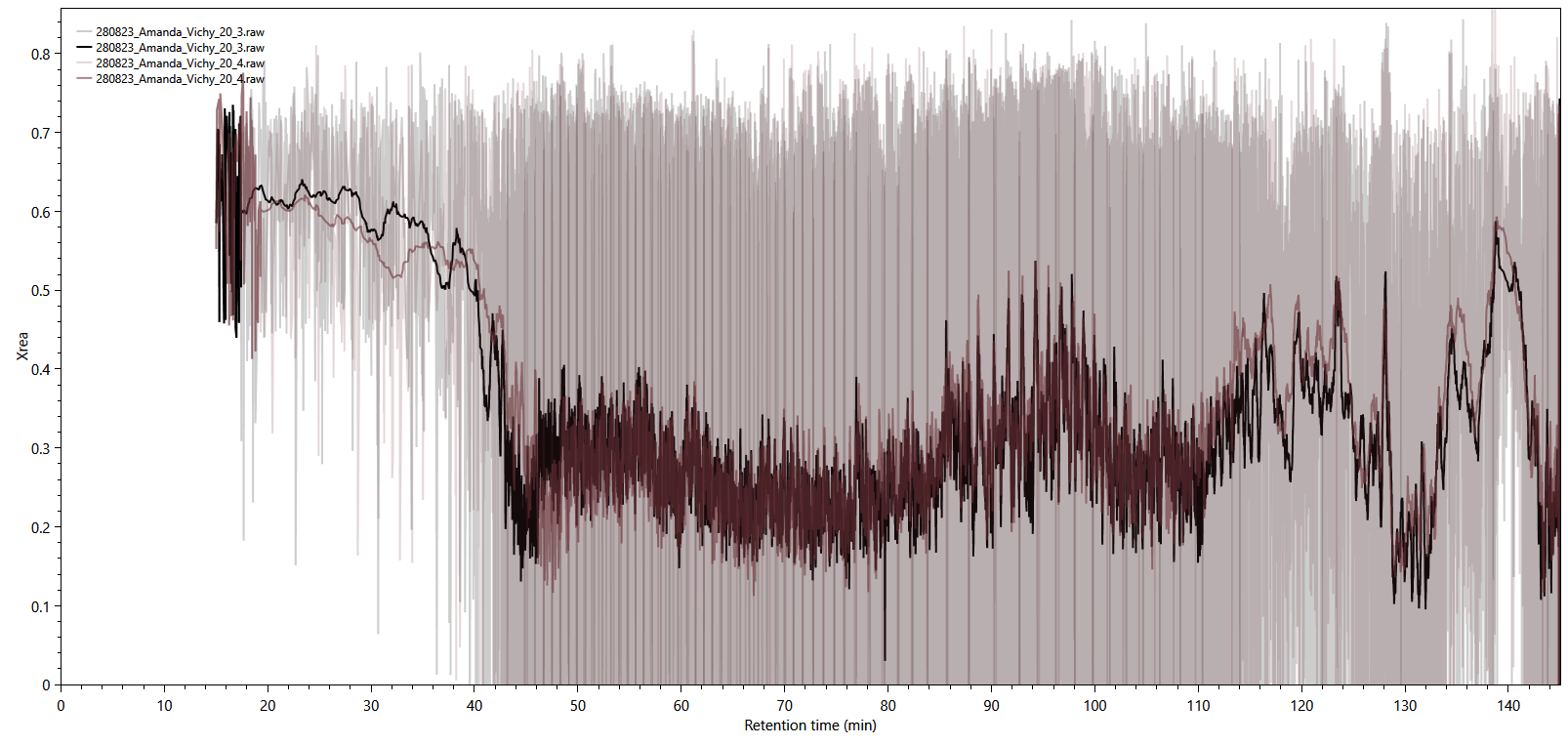


- - **22
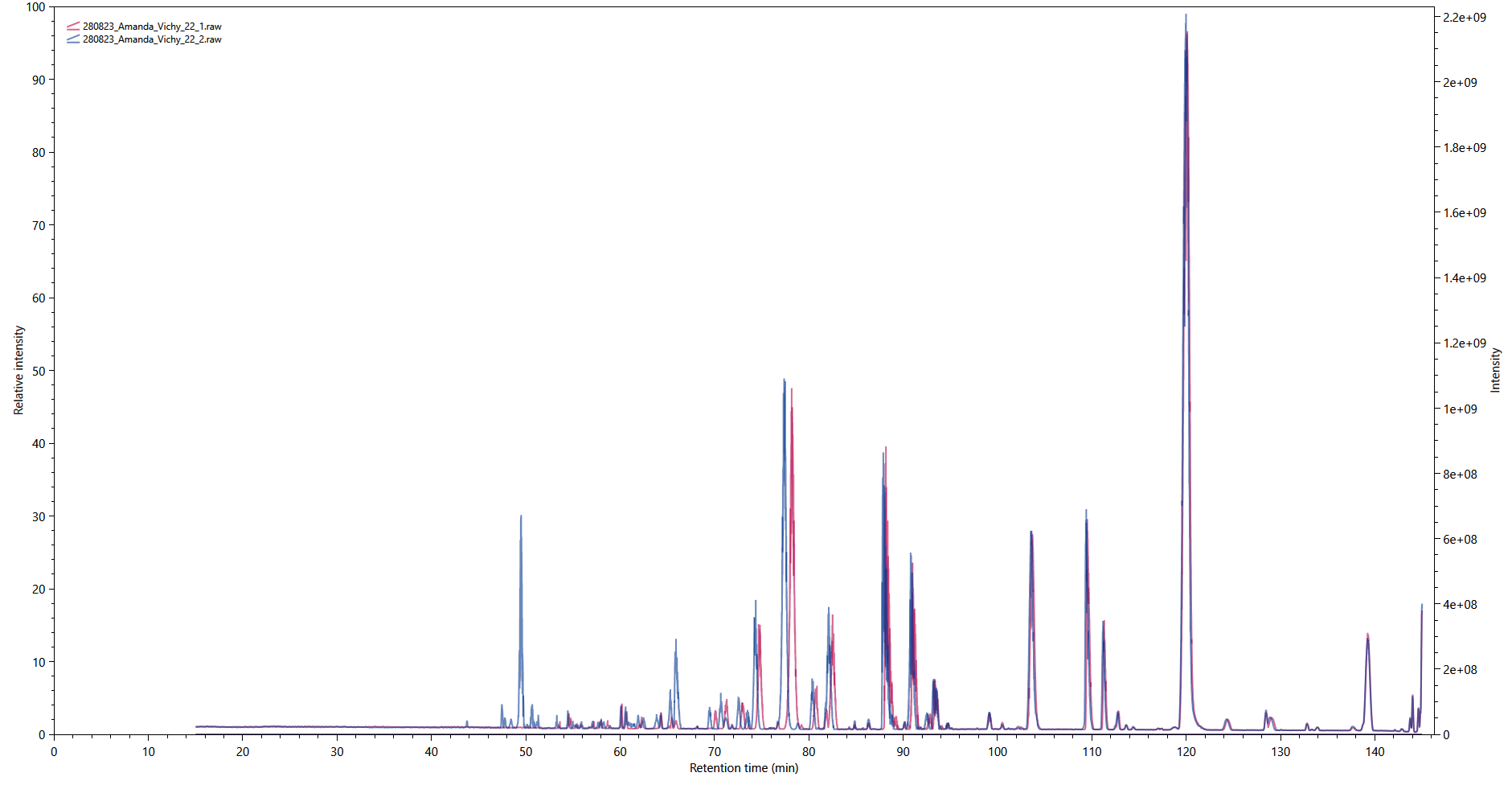

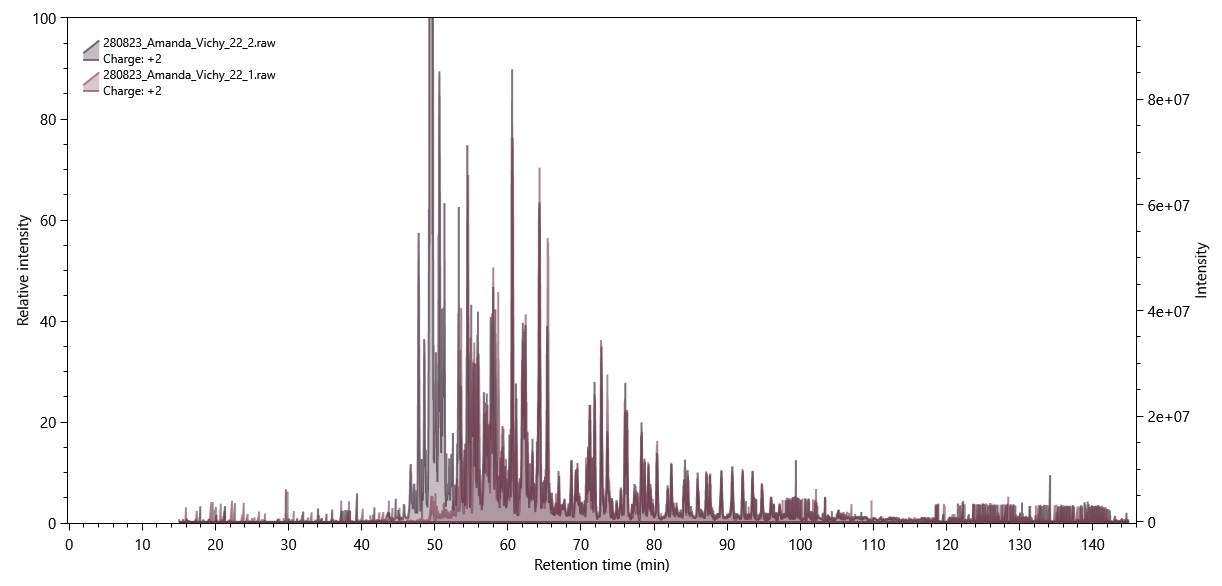

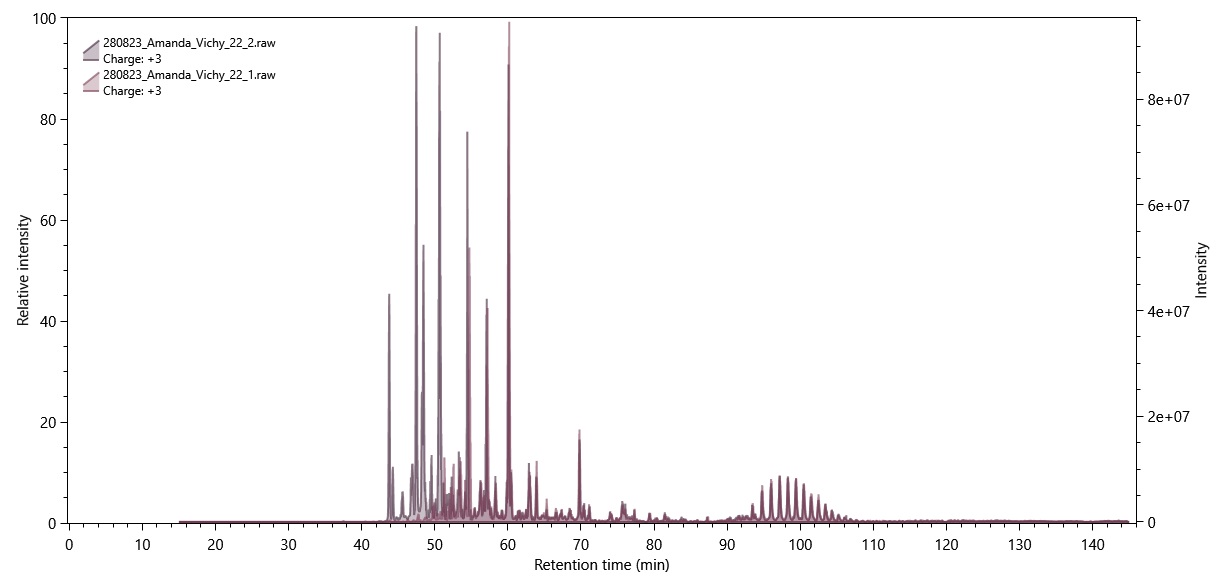

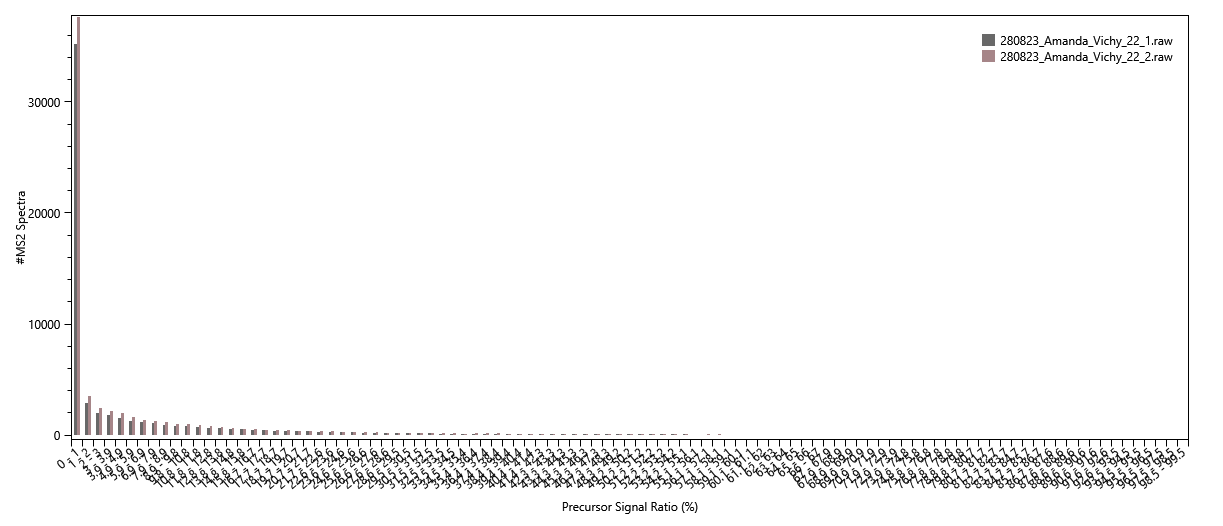

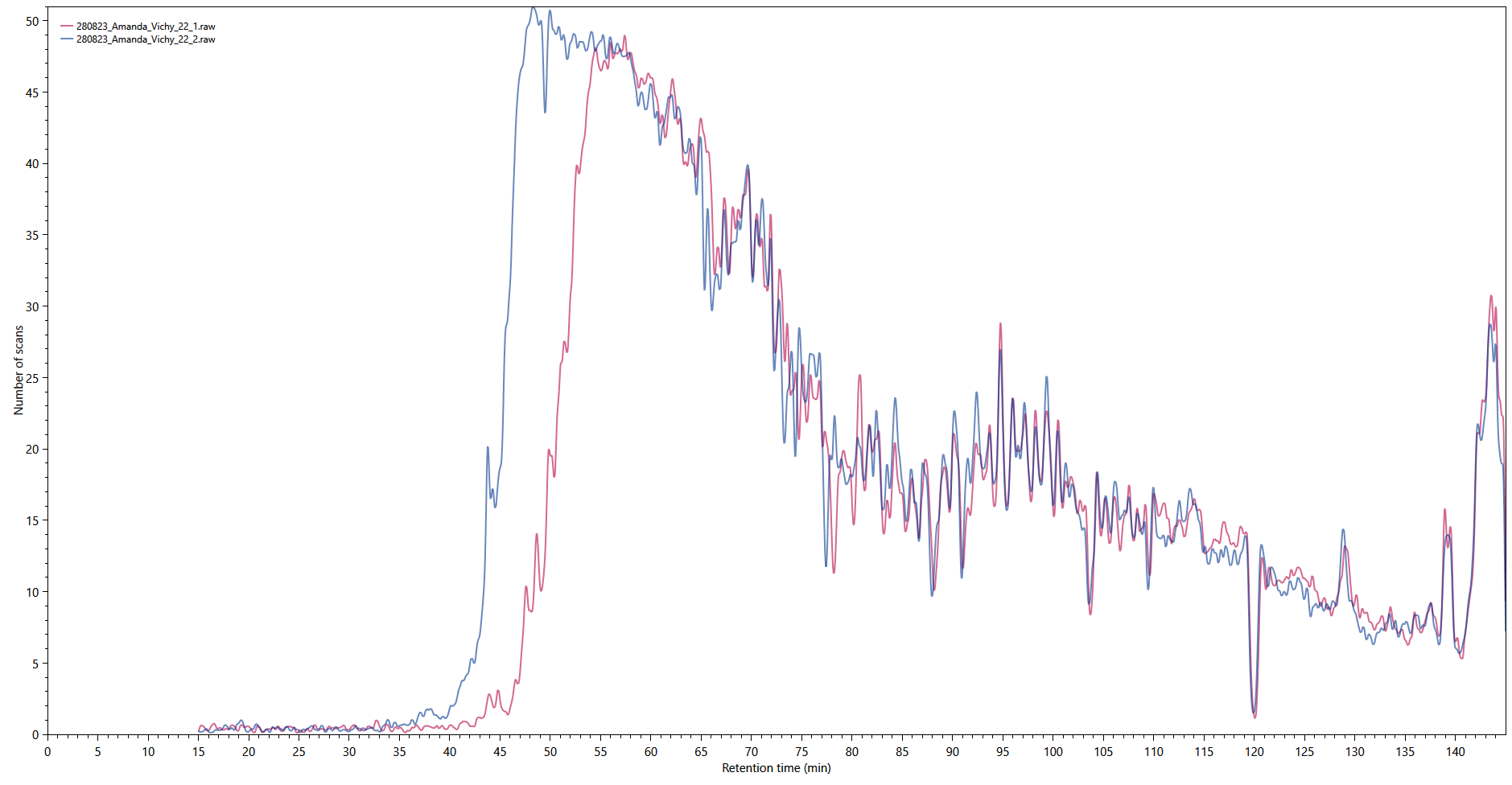

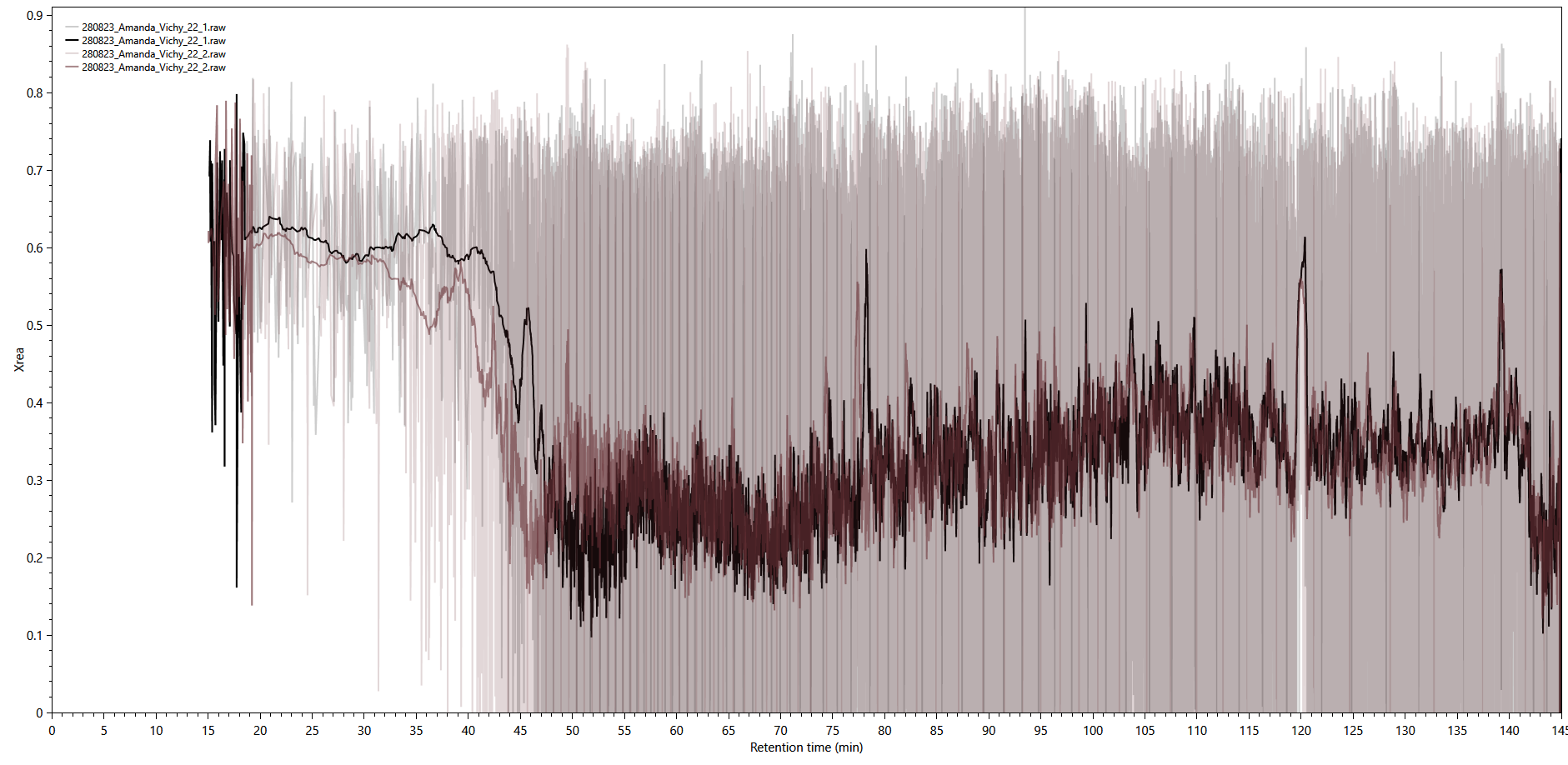
**
- **24**
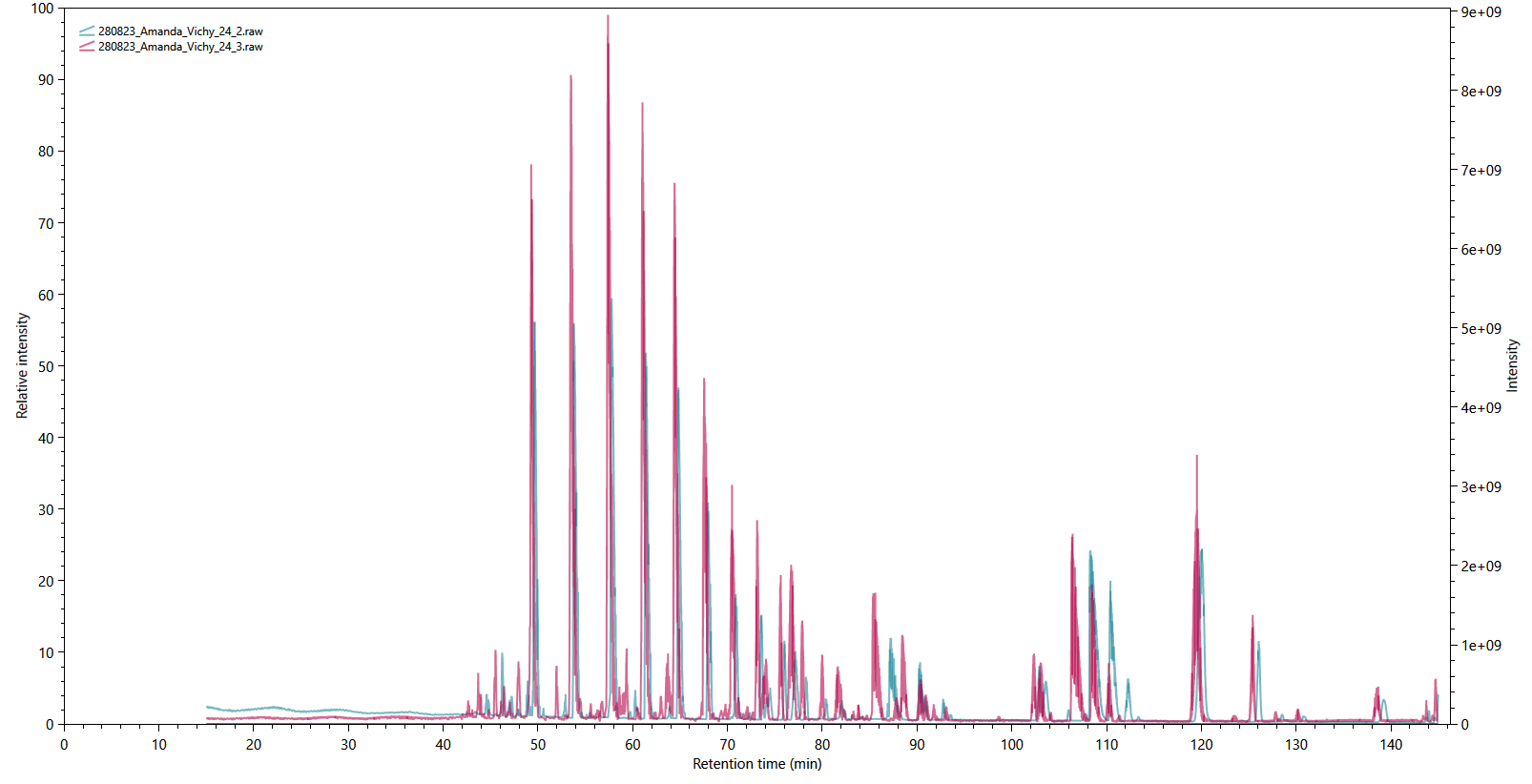

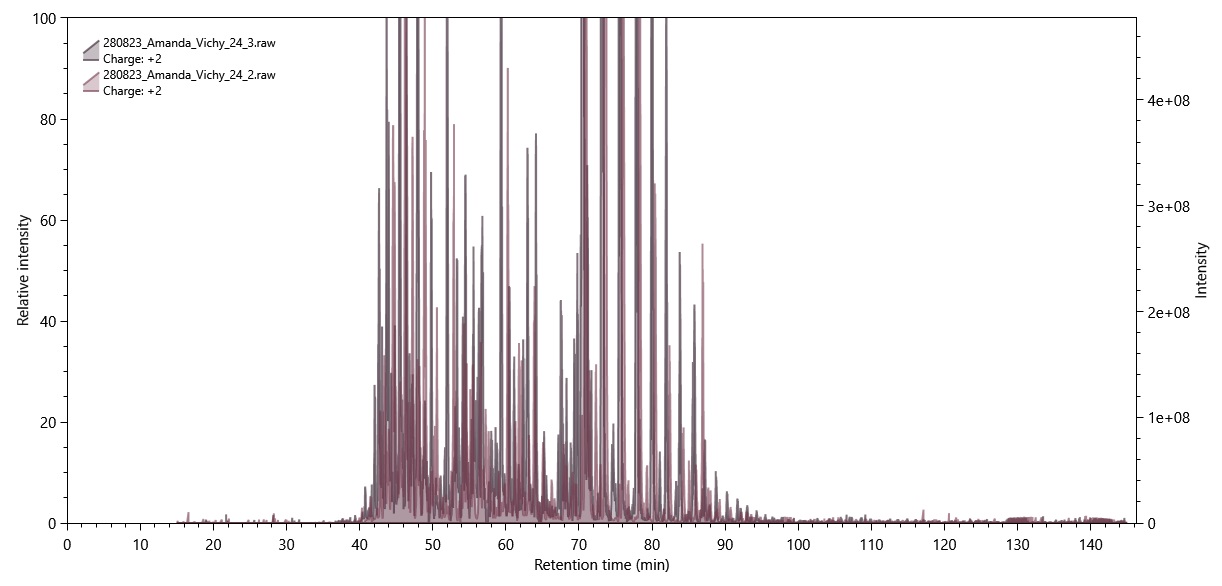

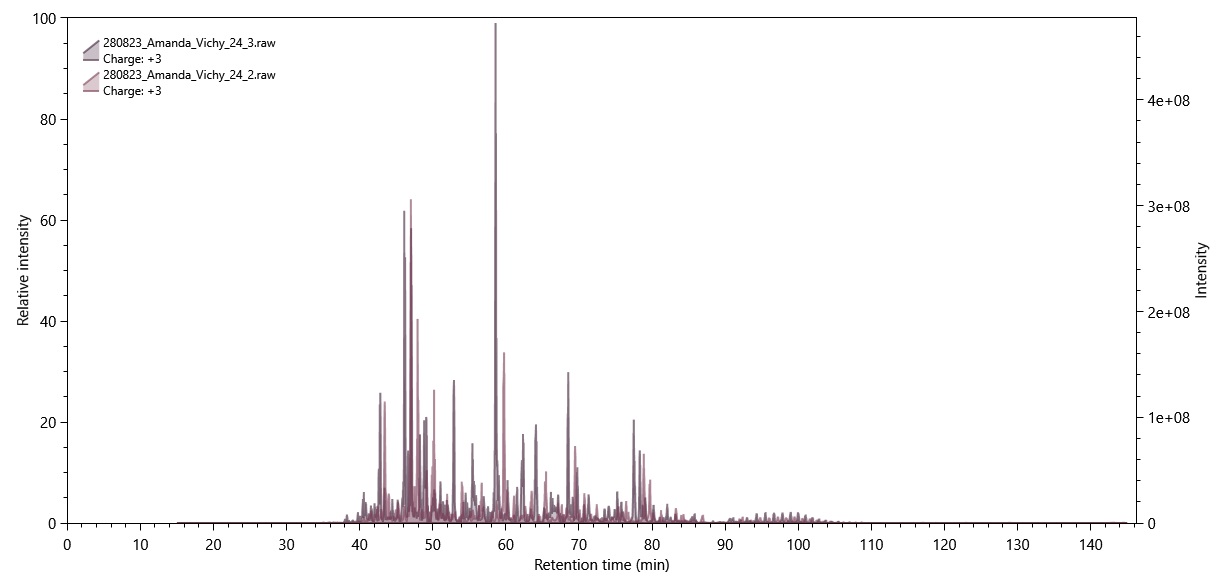

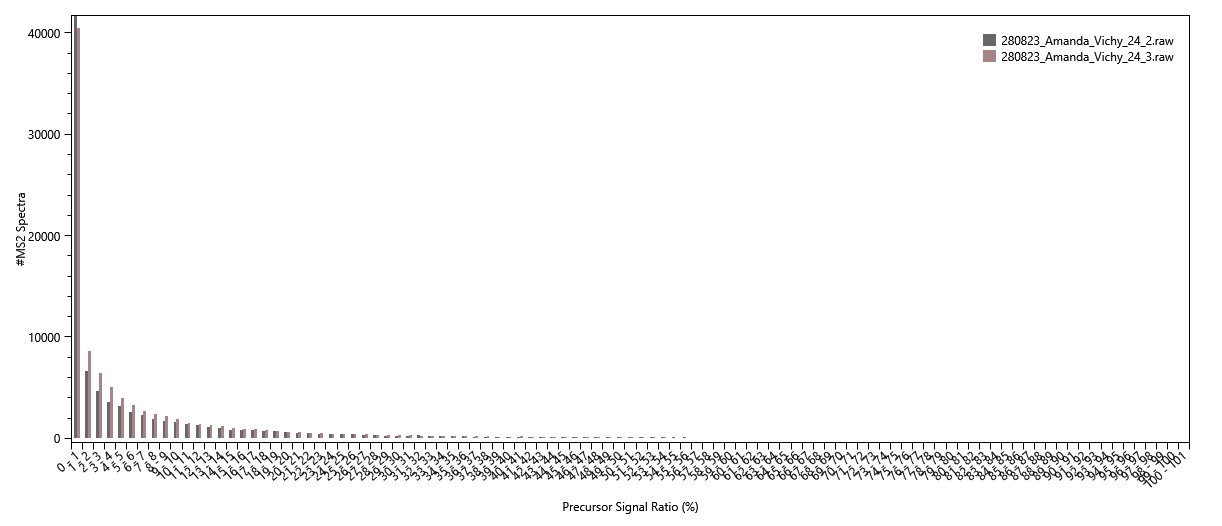

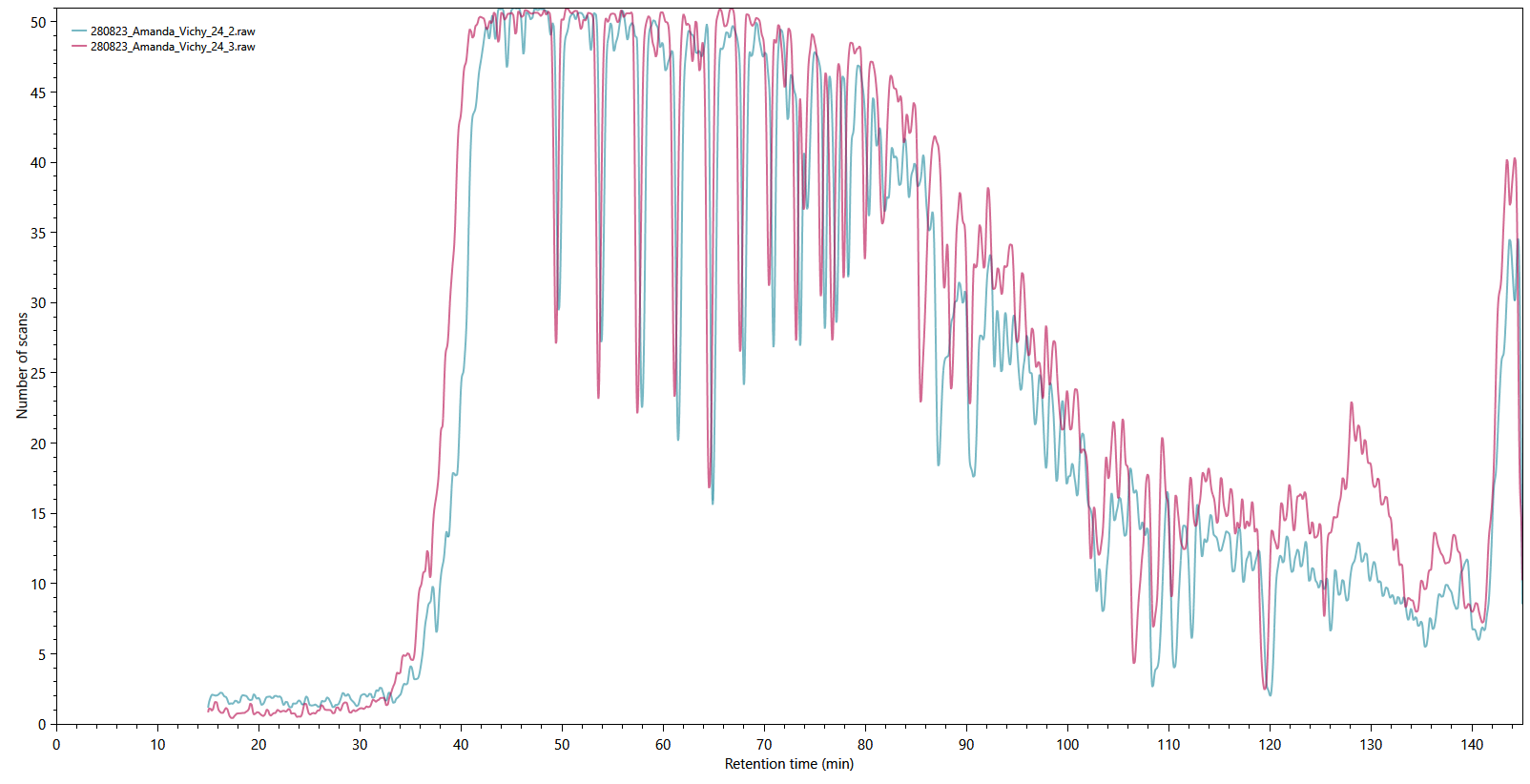

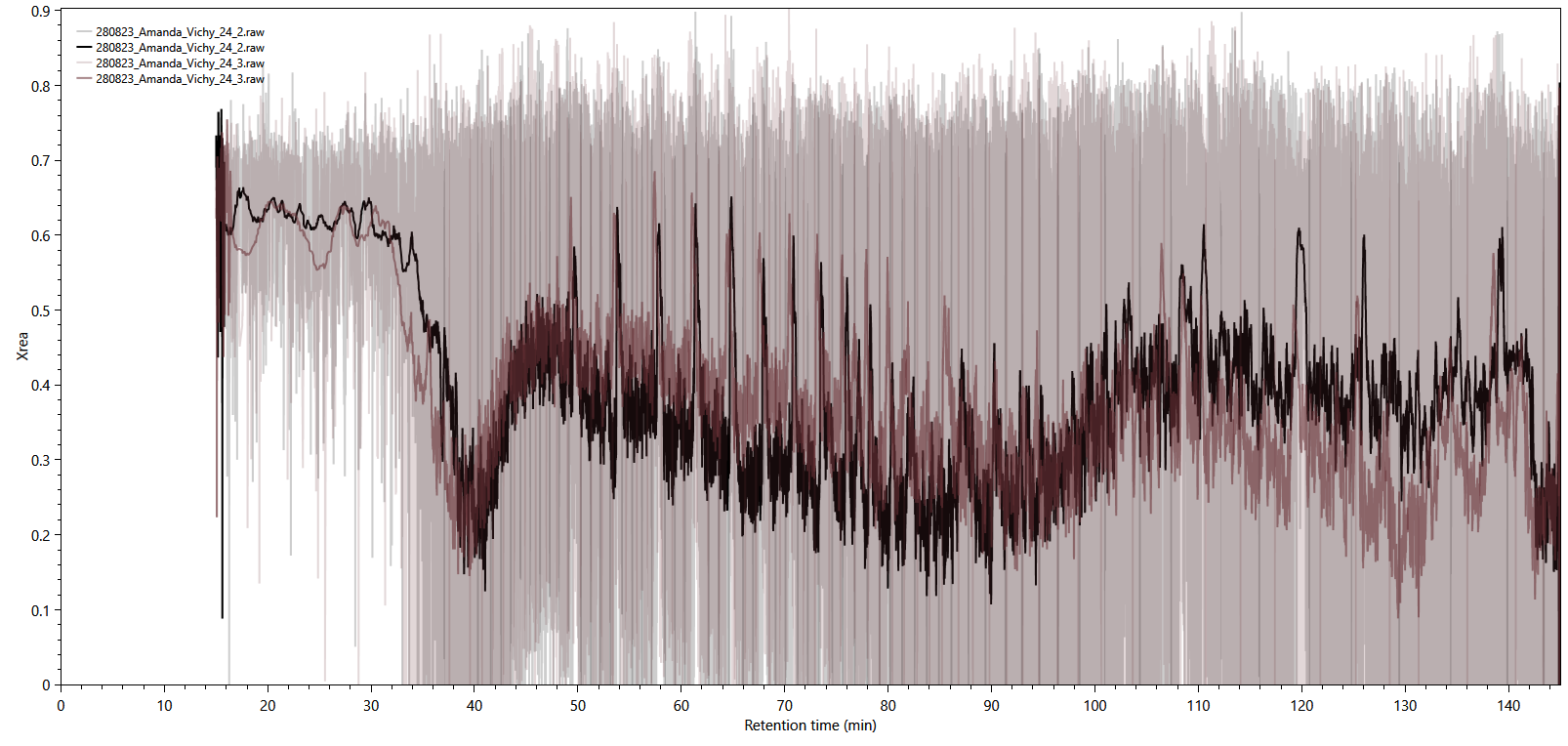

- **30
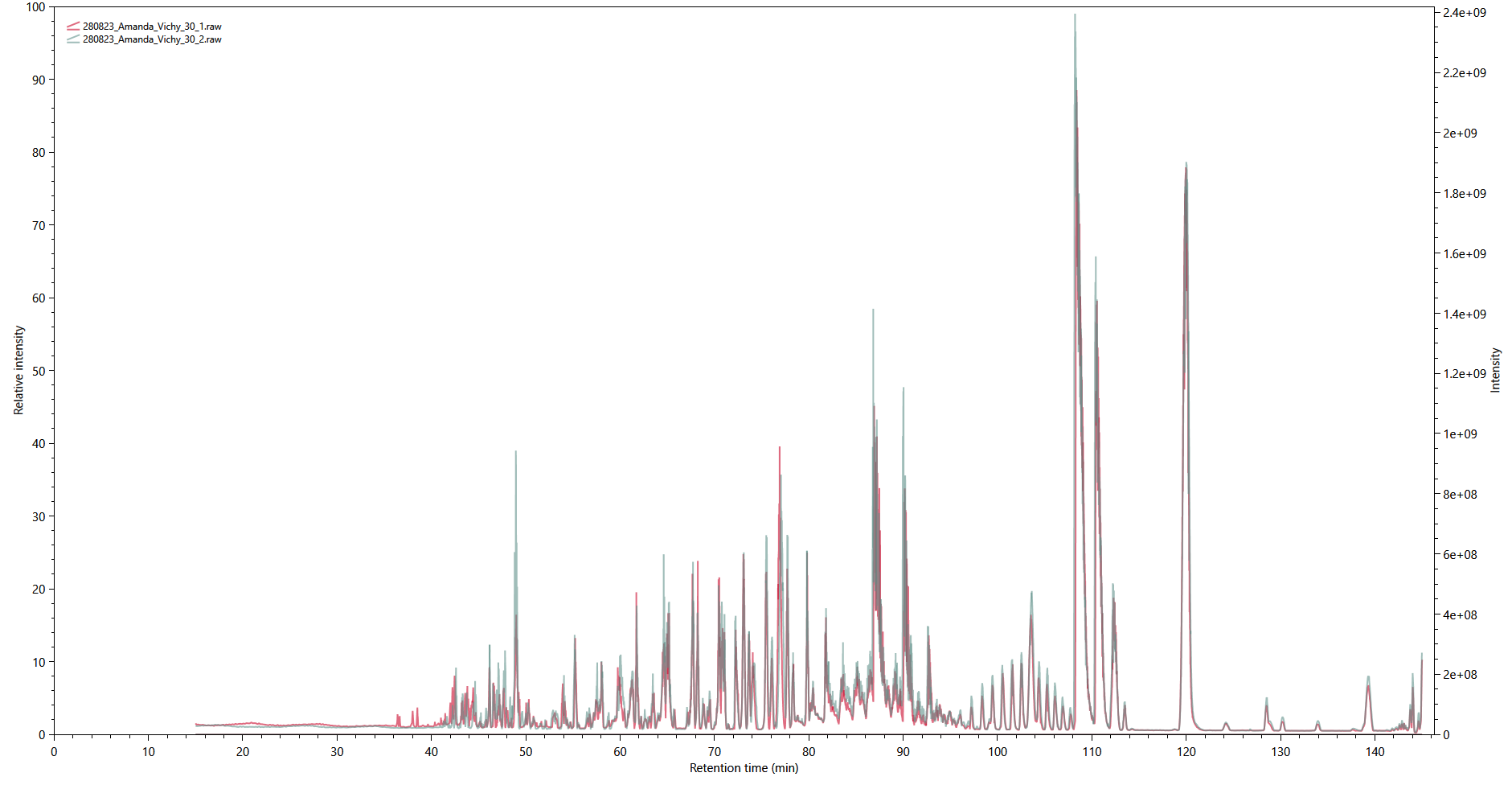

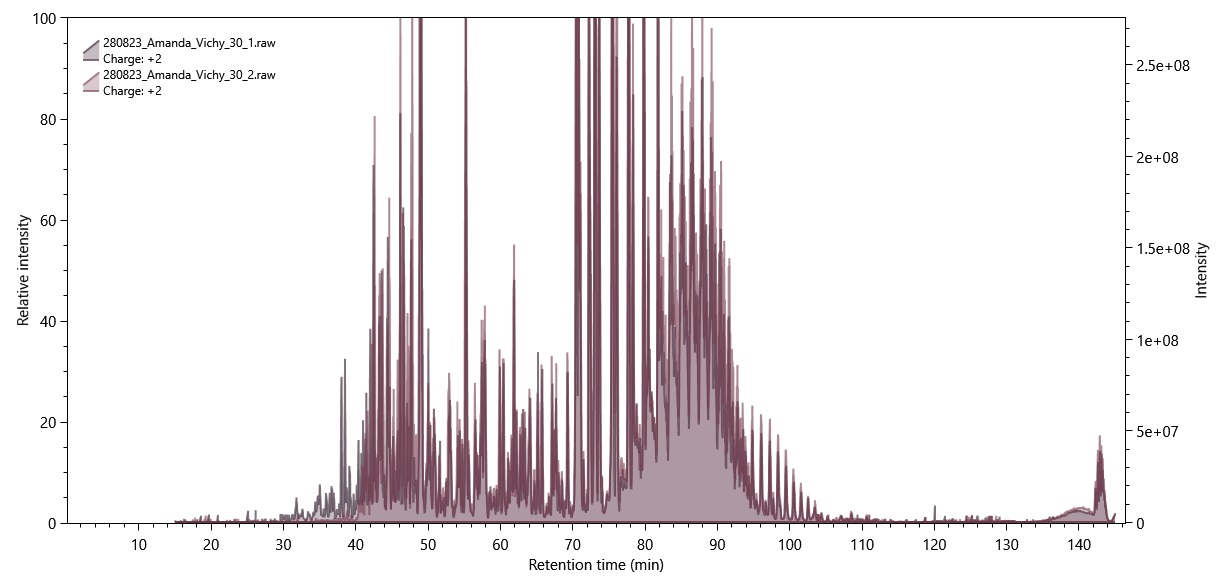

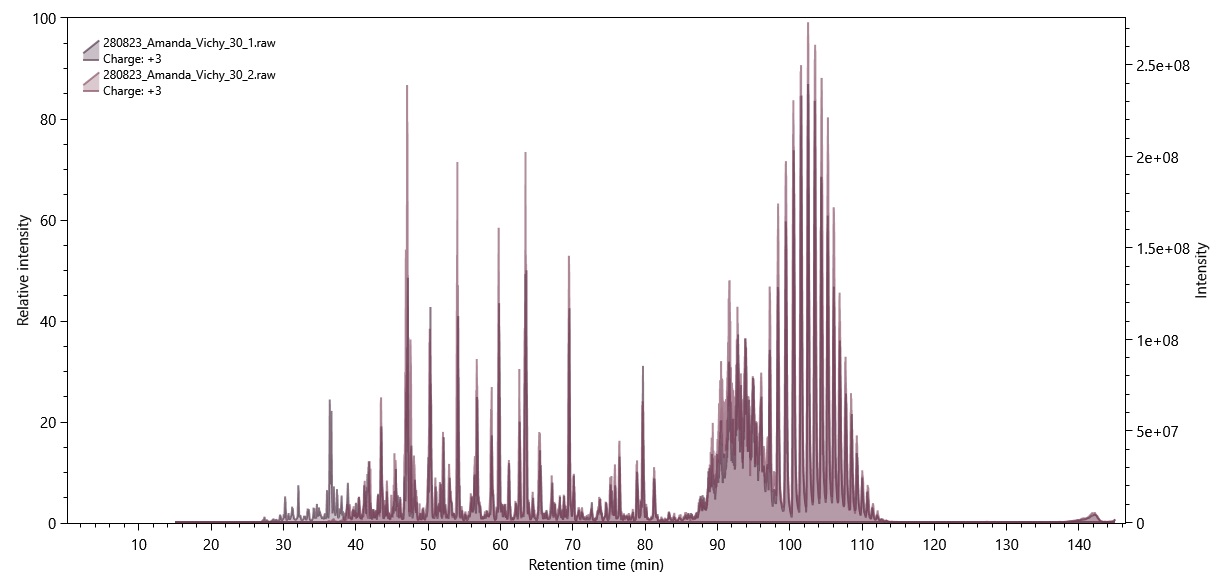

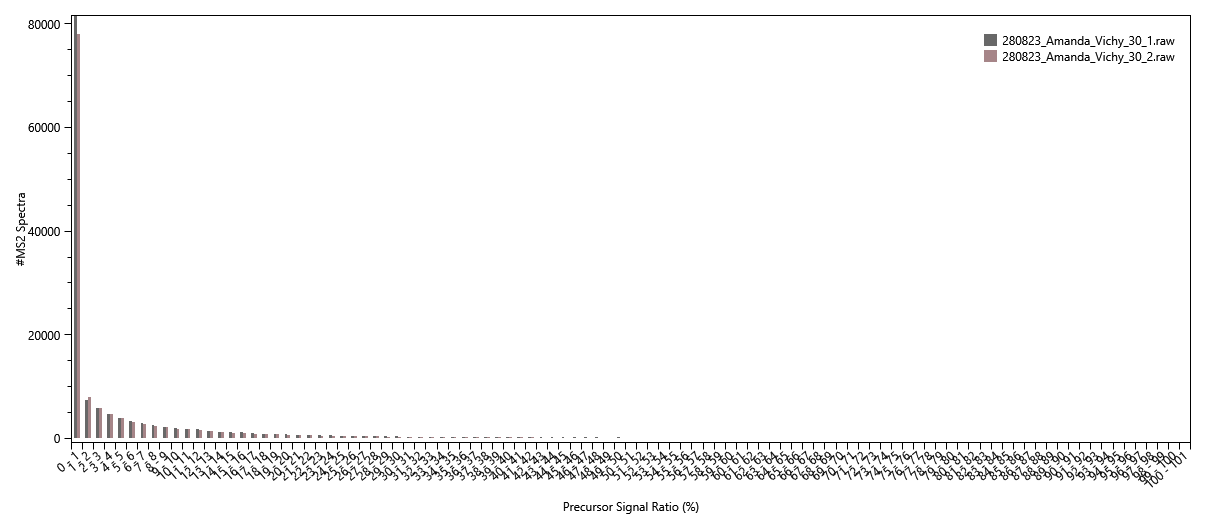

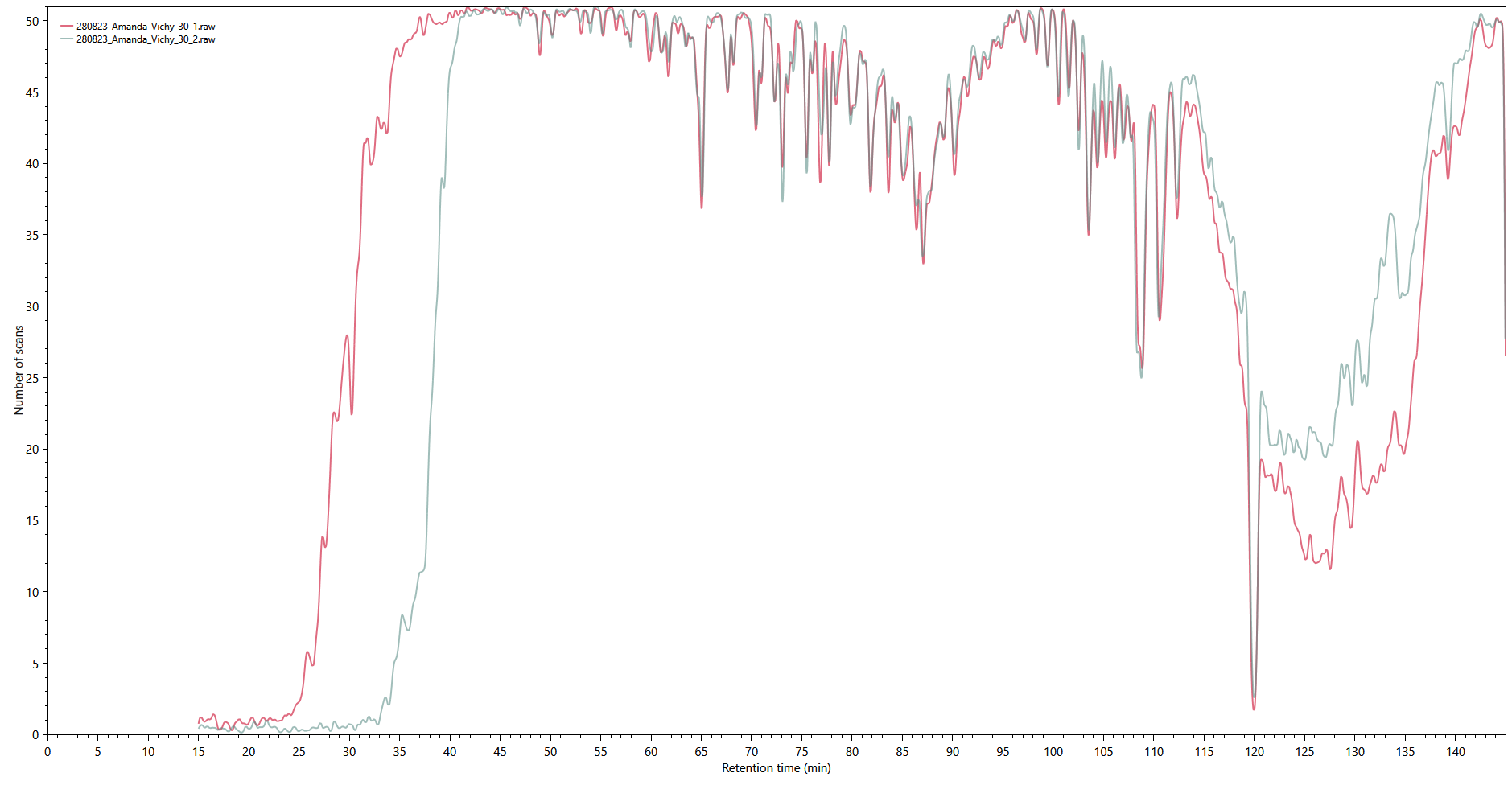

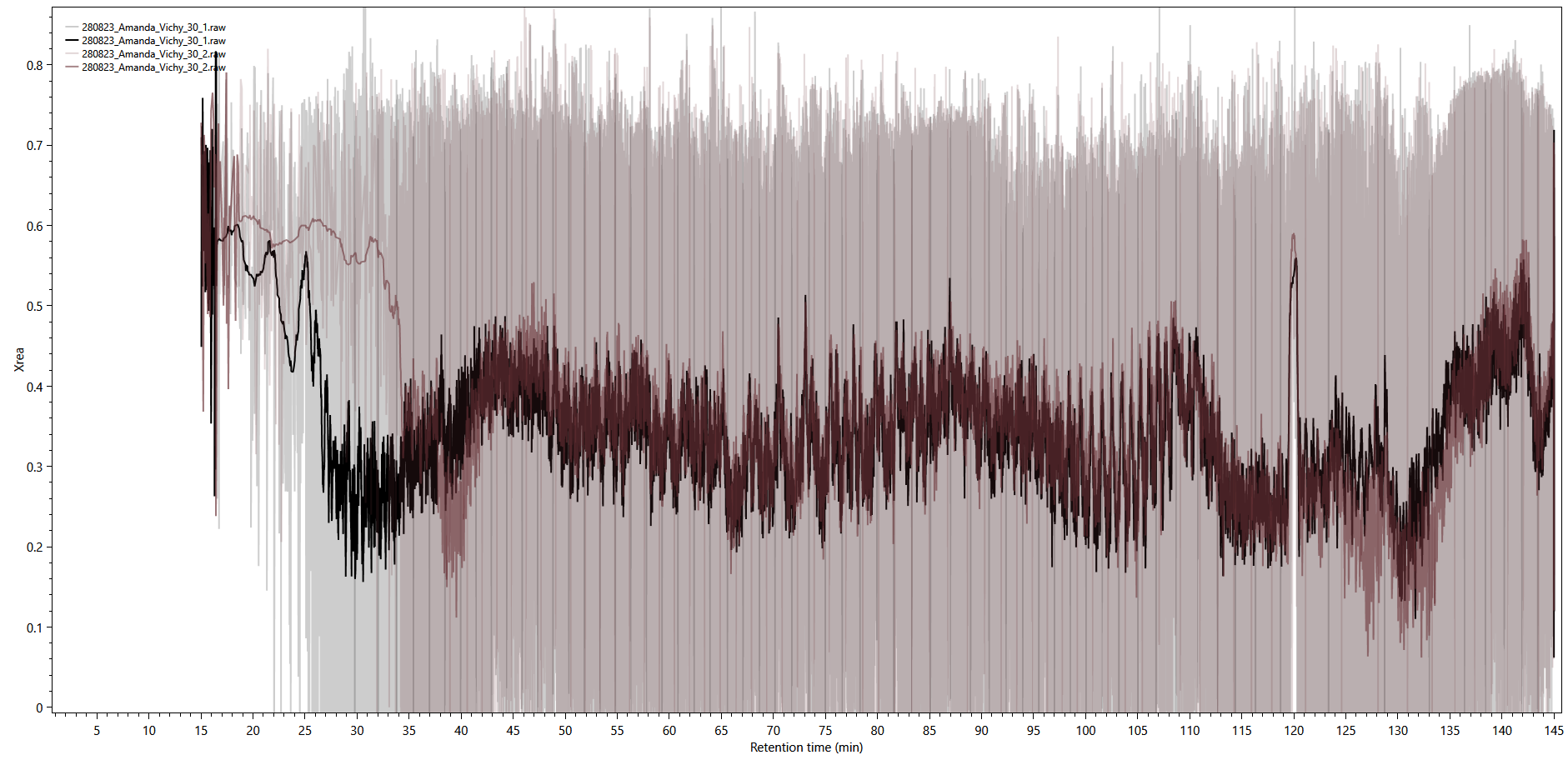
**
- **32
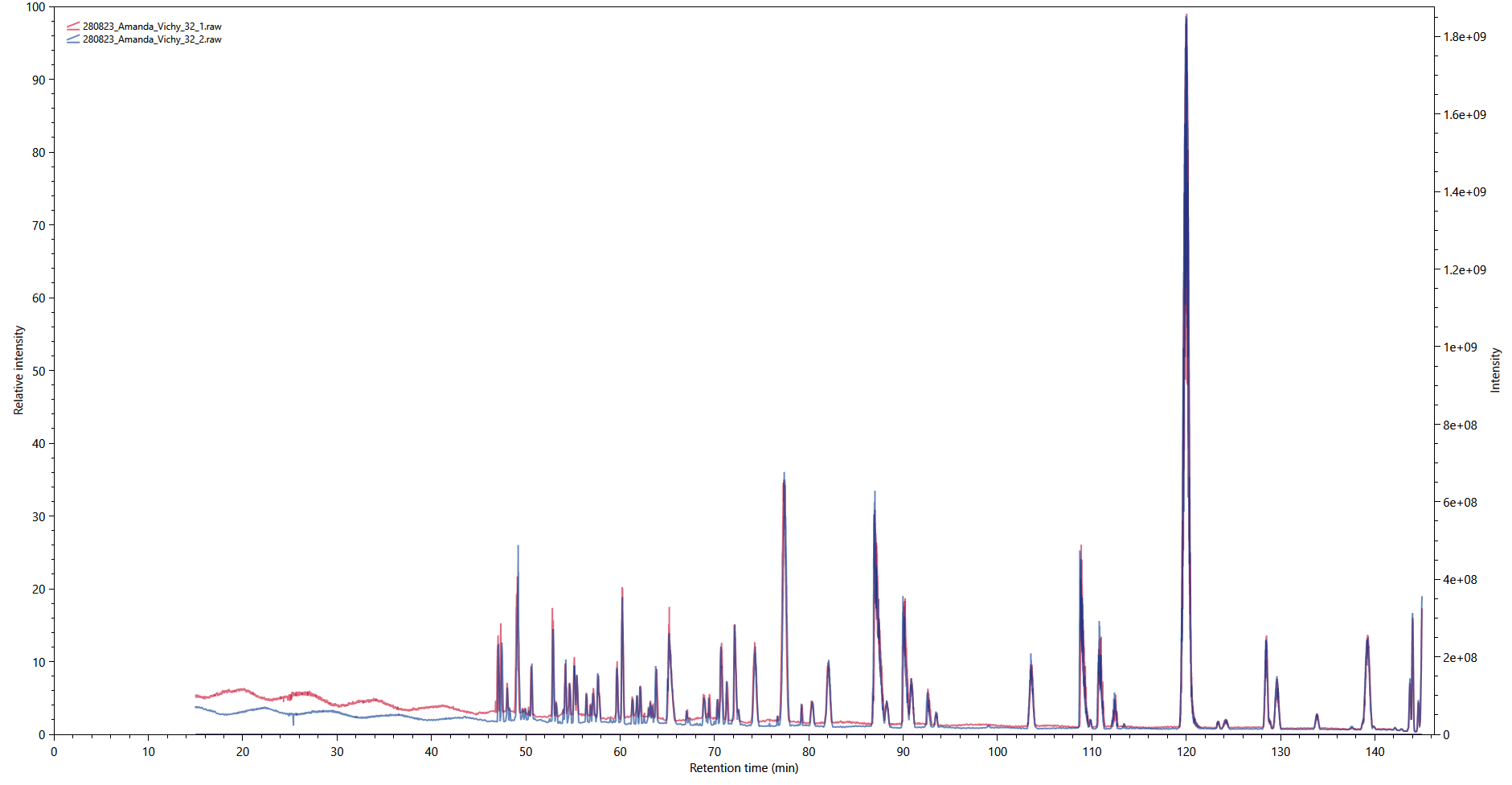

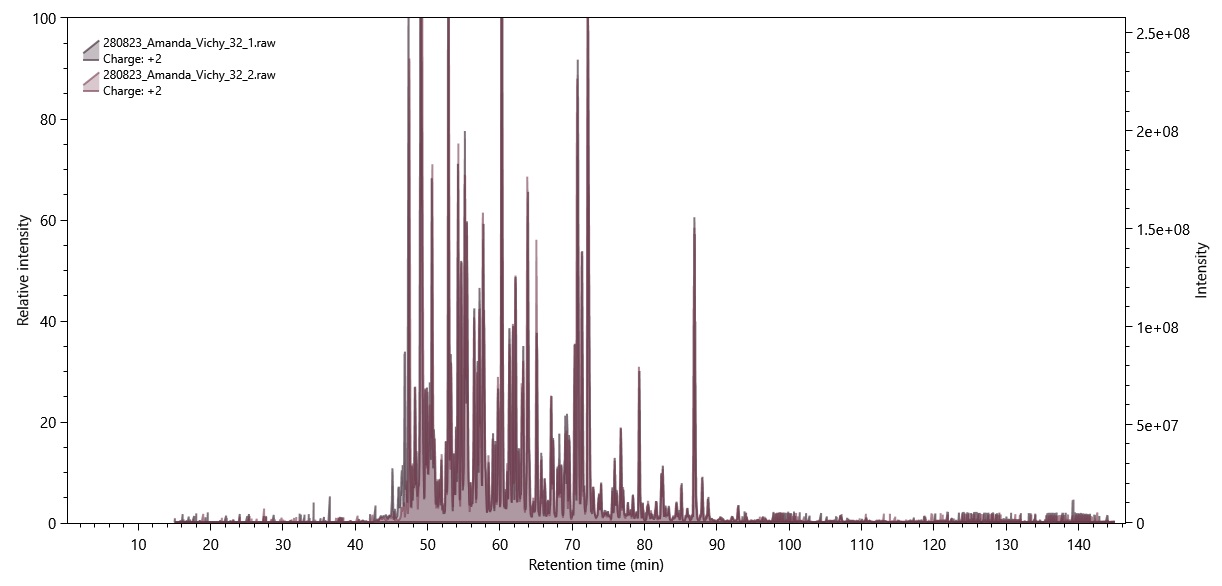

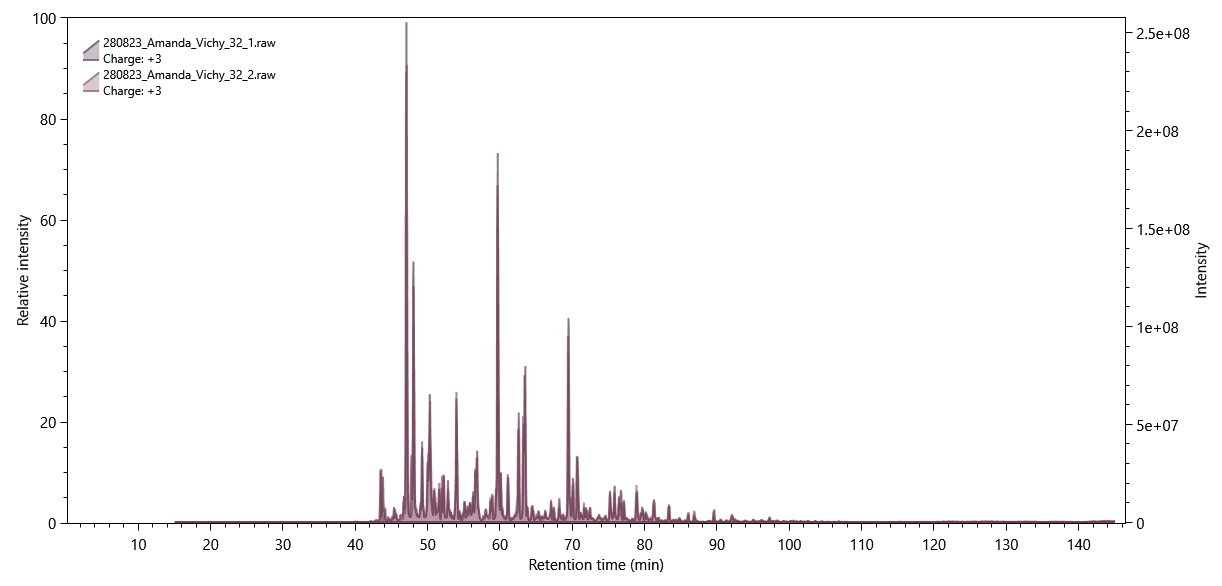

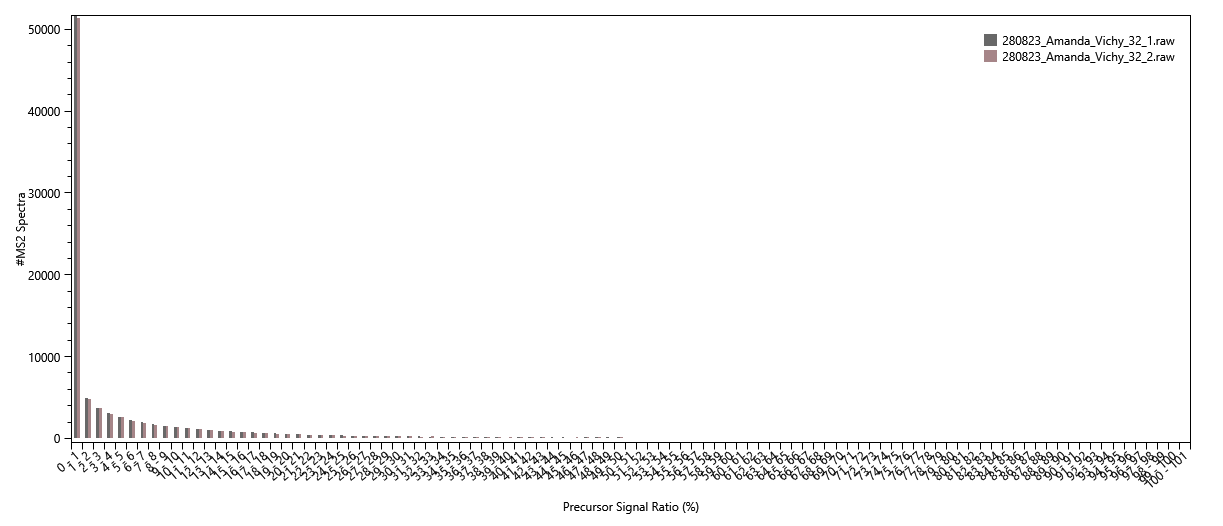

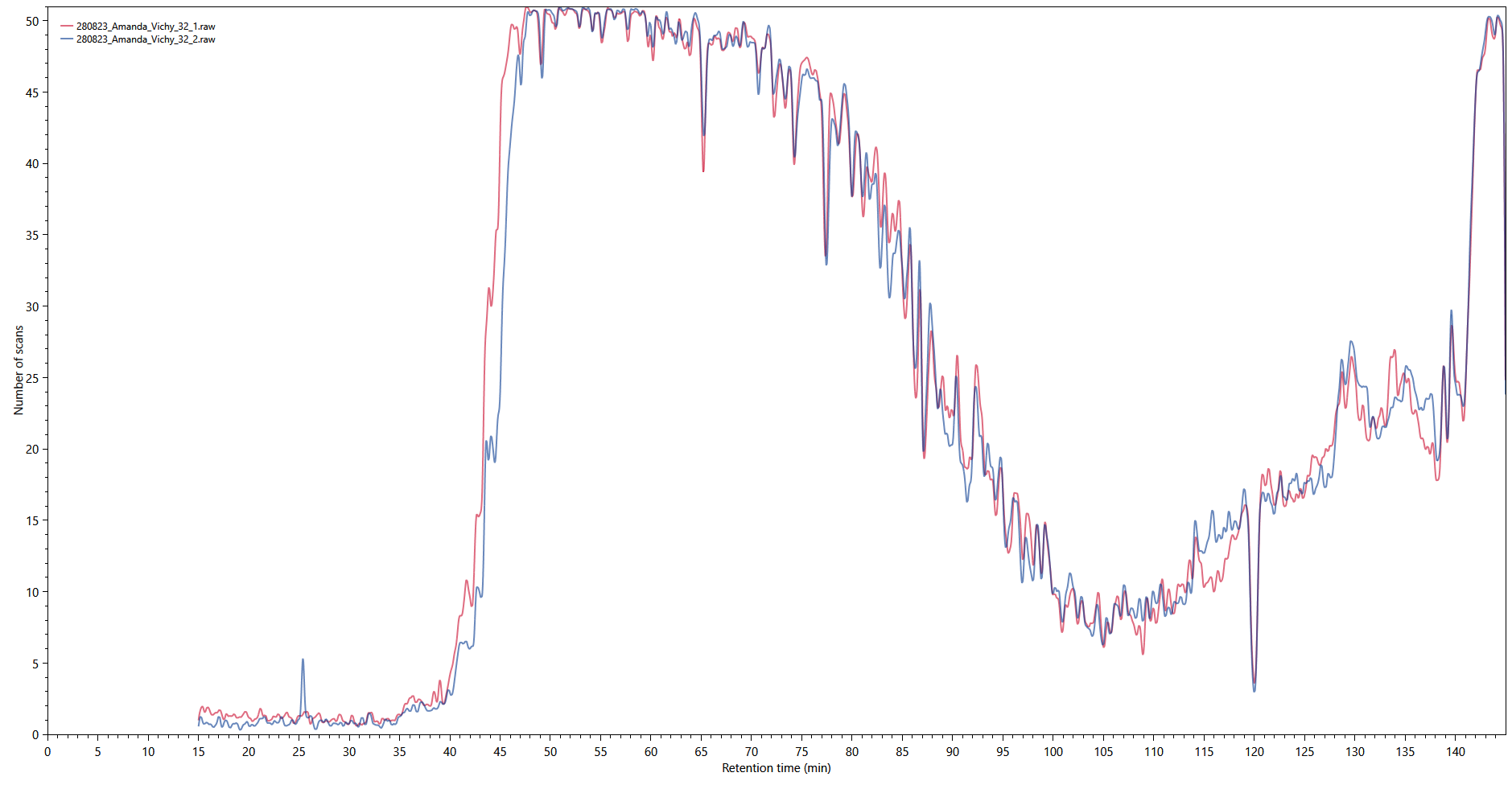

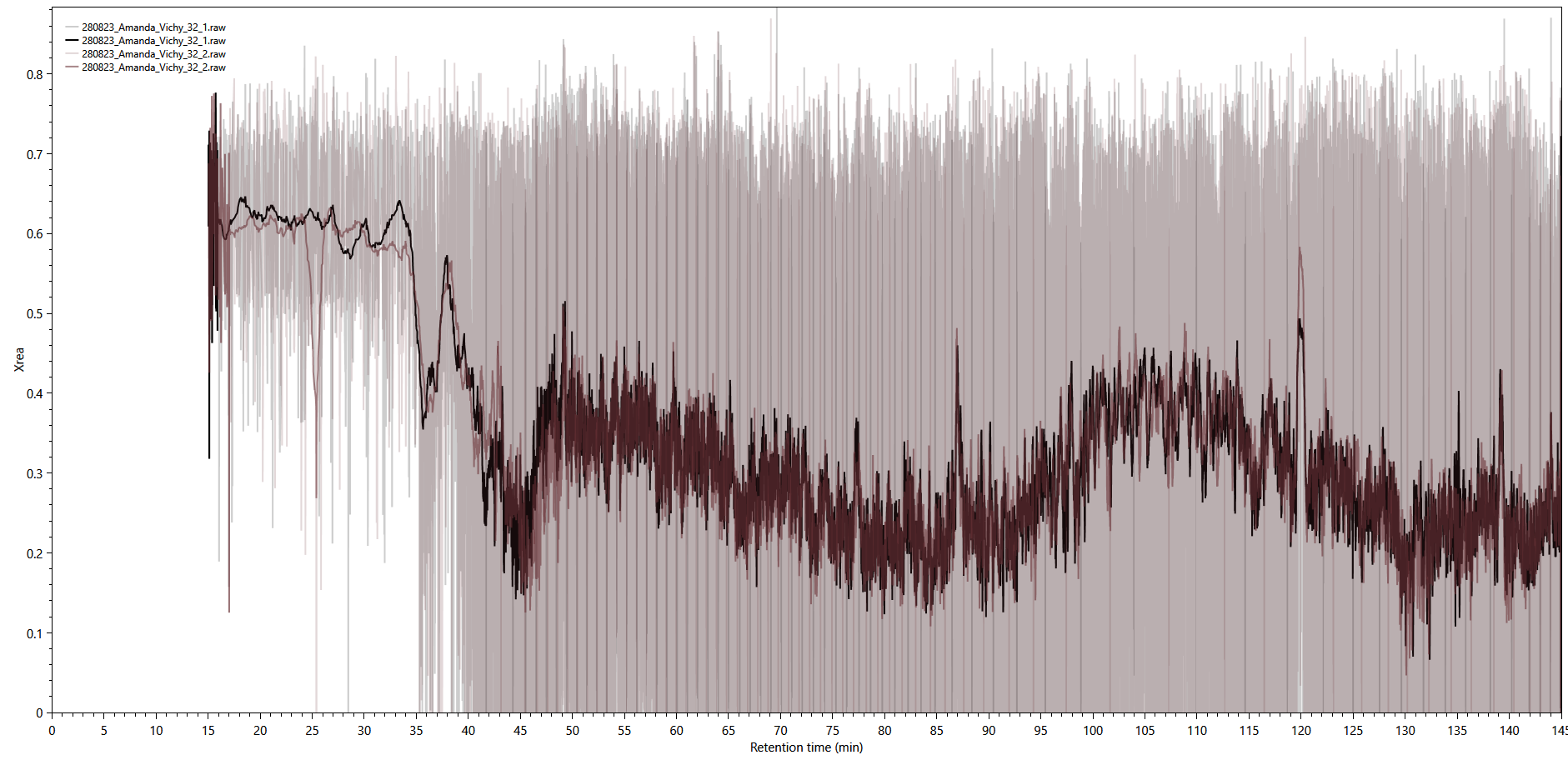
**

**34
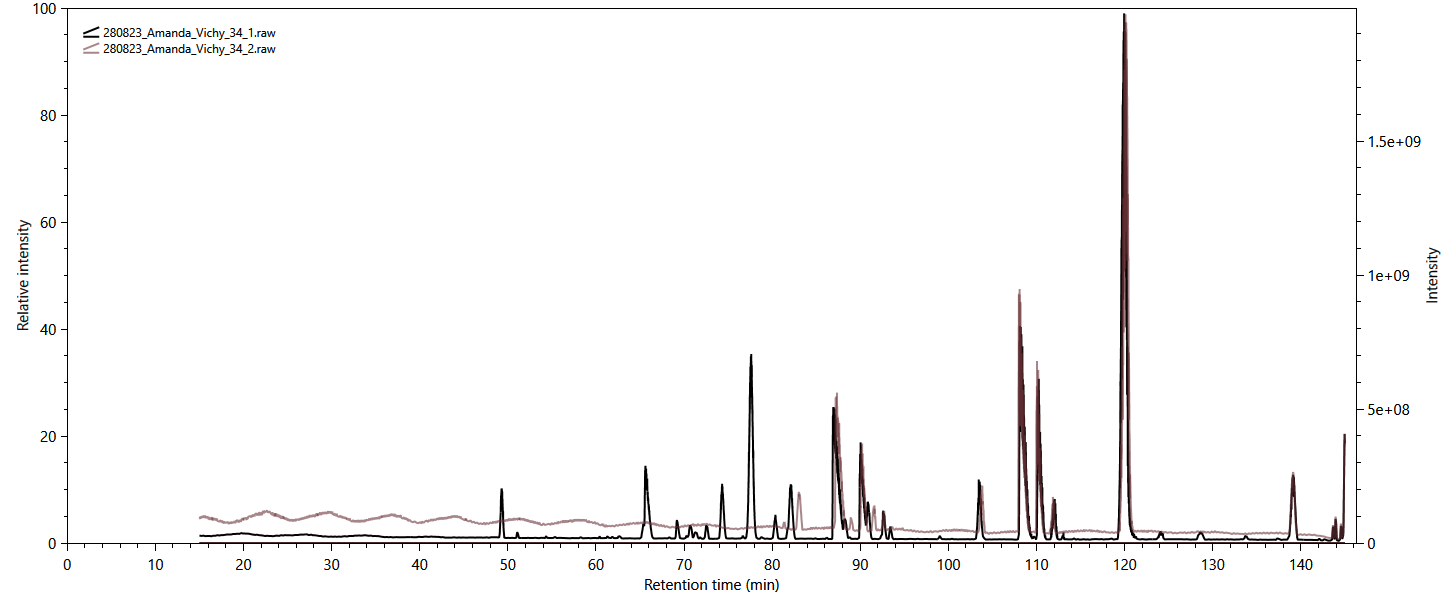
**

**
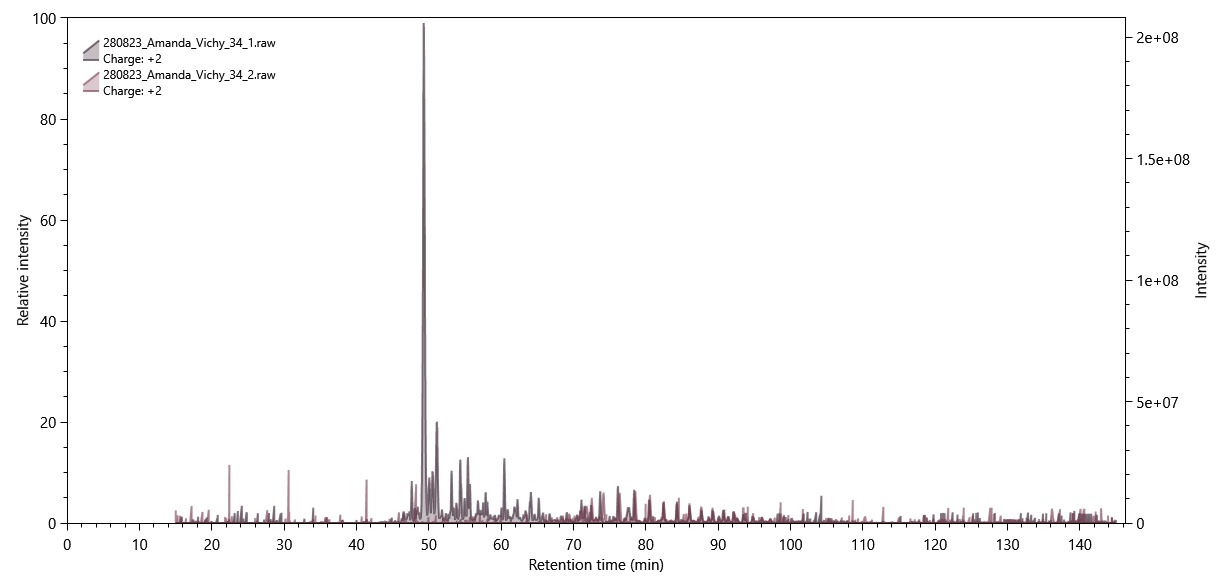
**

**
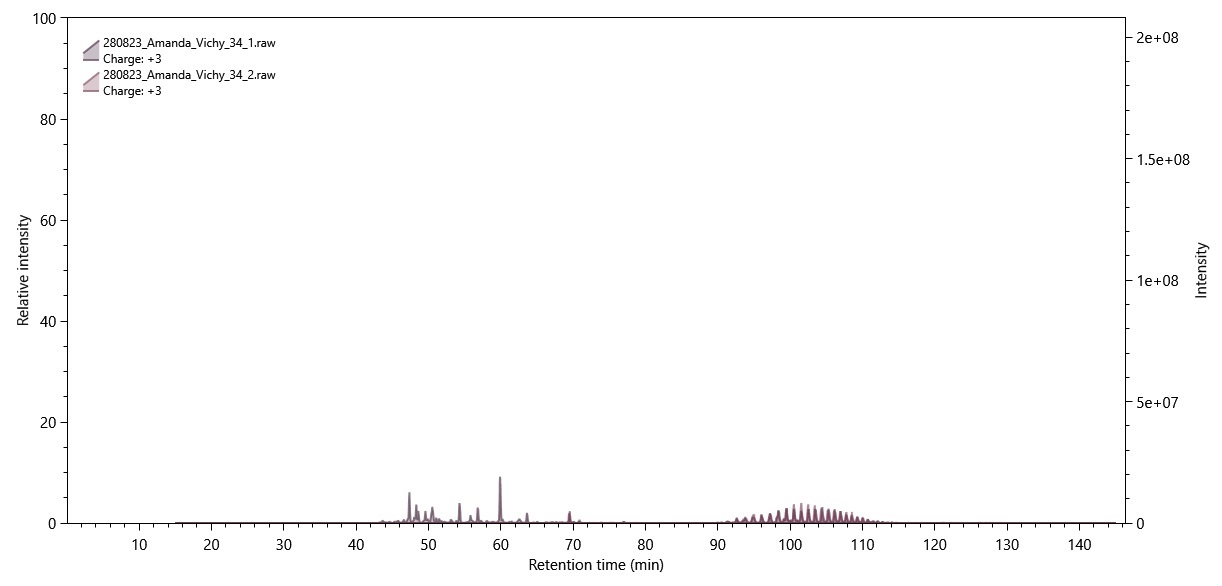

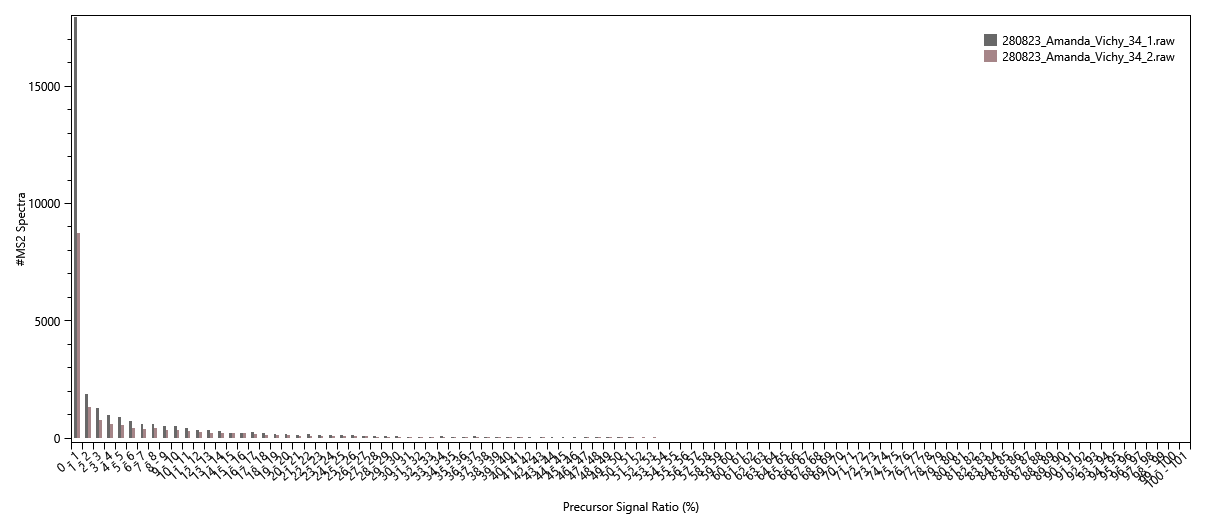

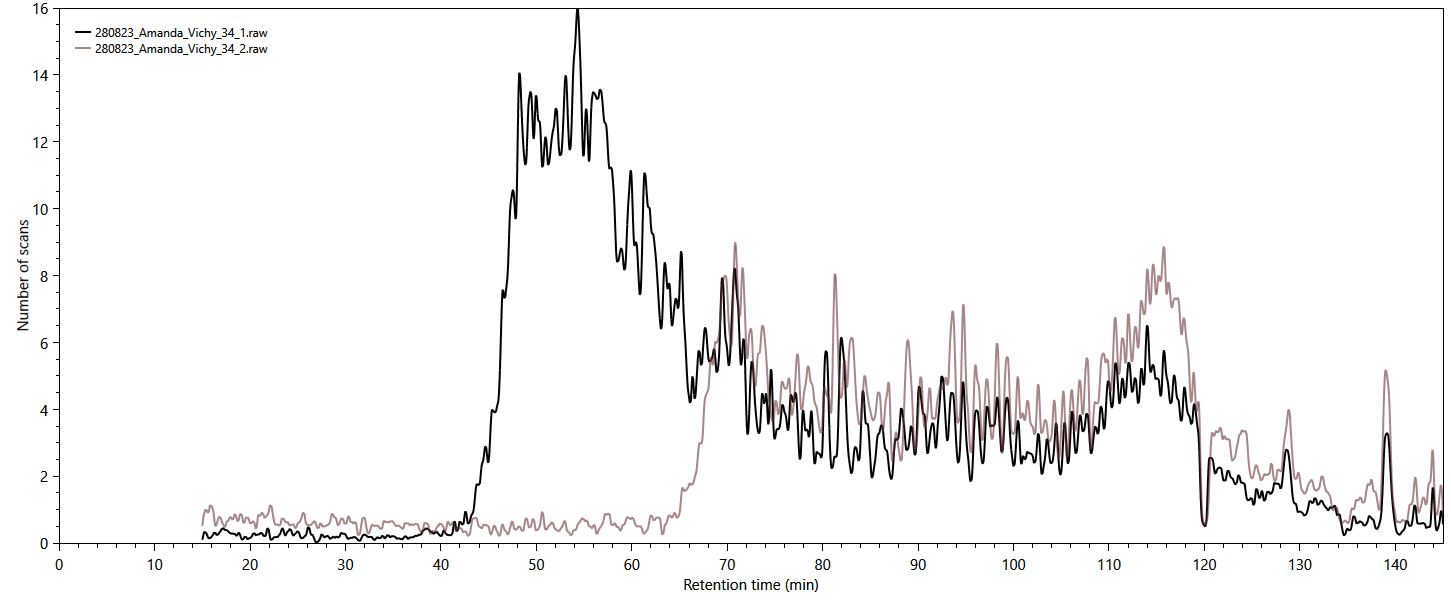

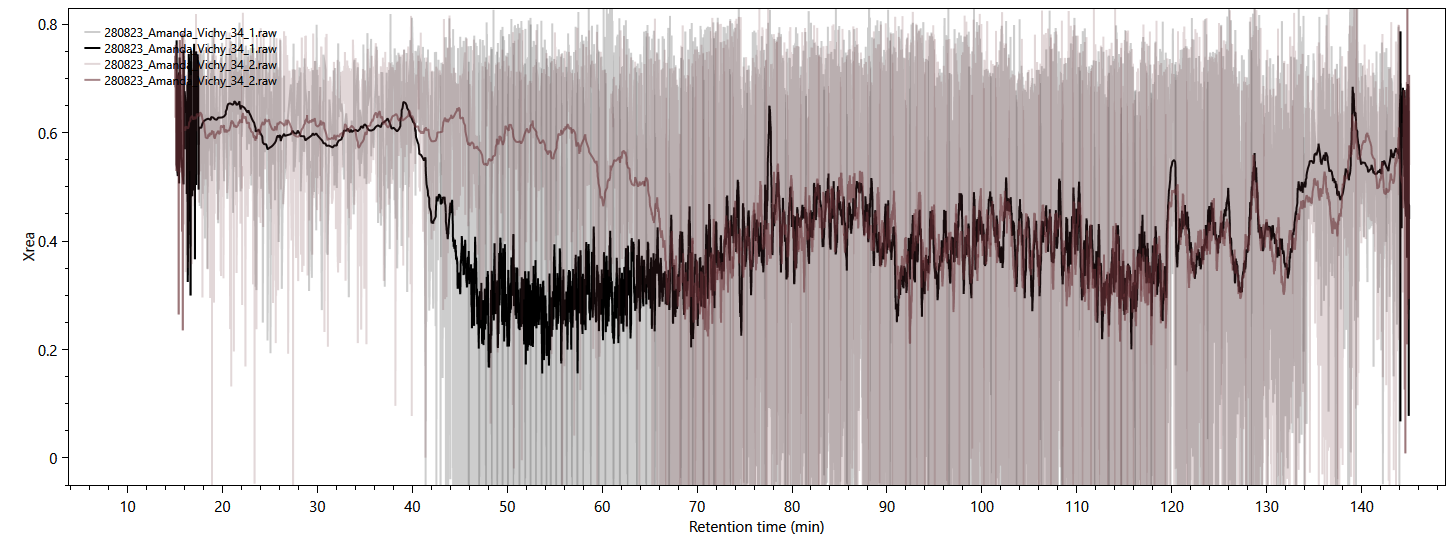
**

1. **RIGHT**

- **07
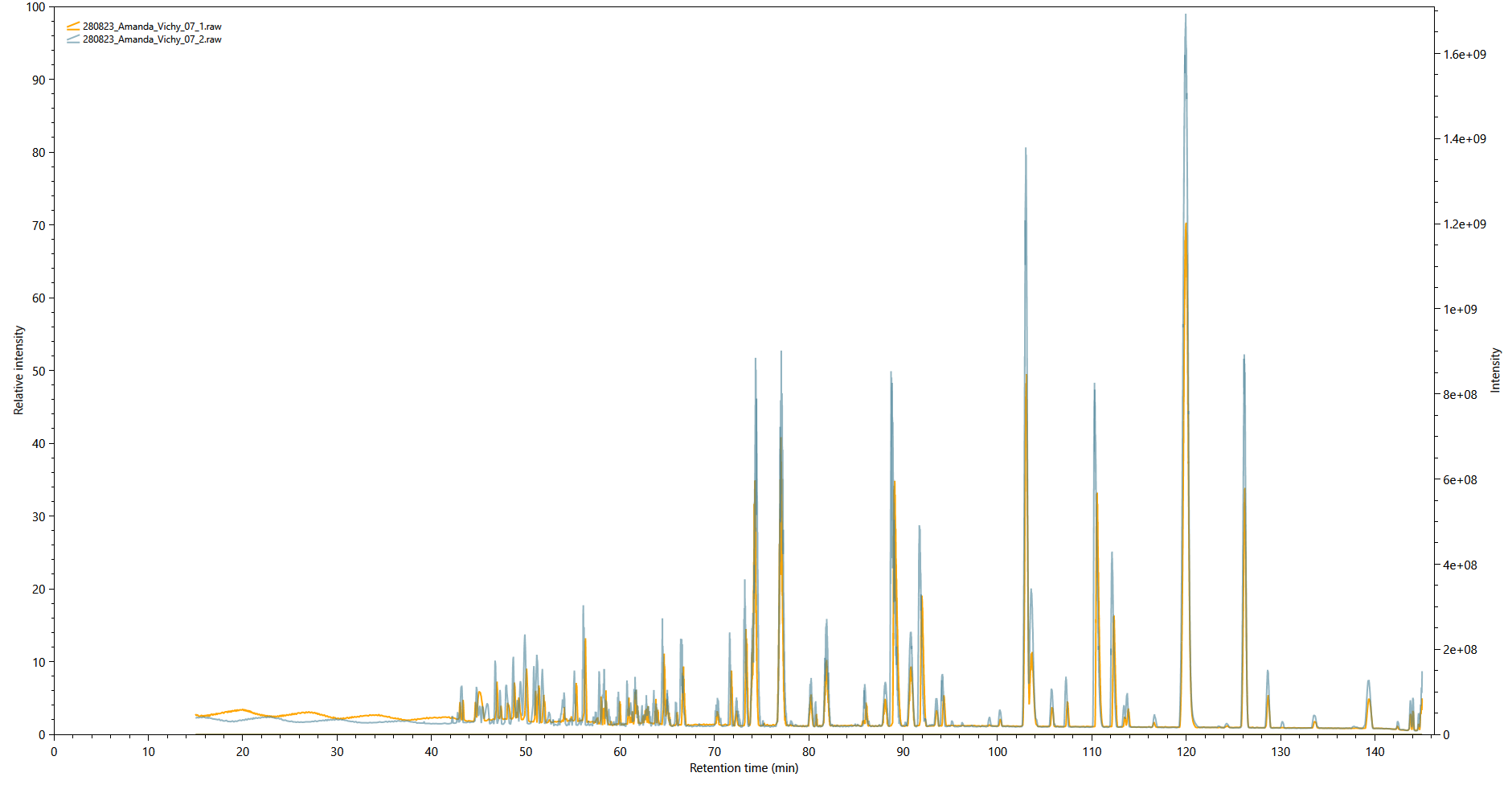

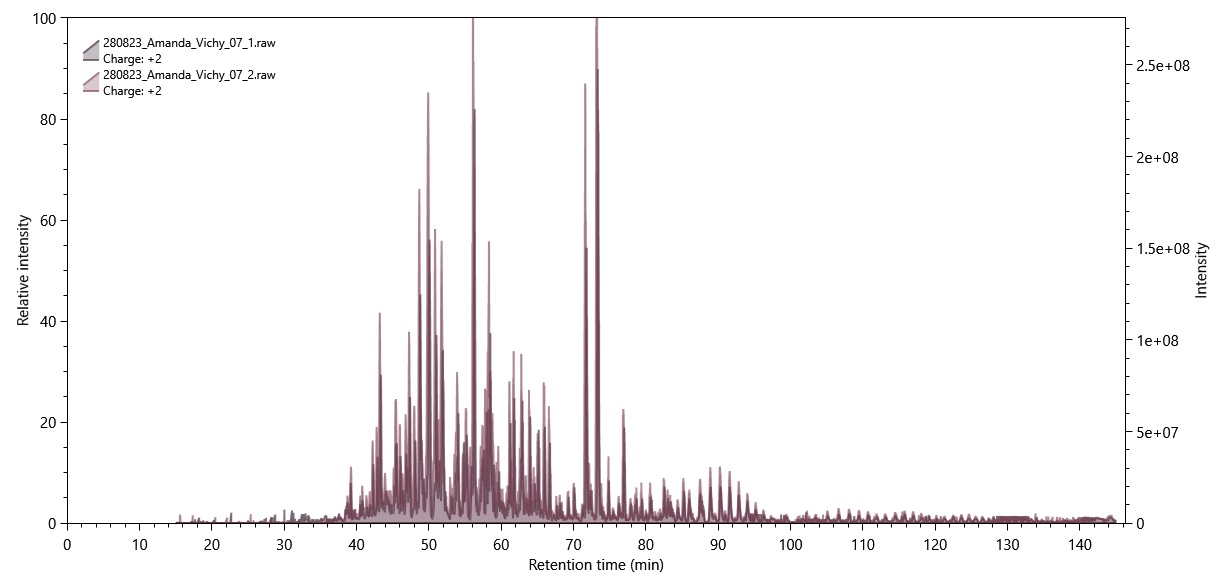

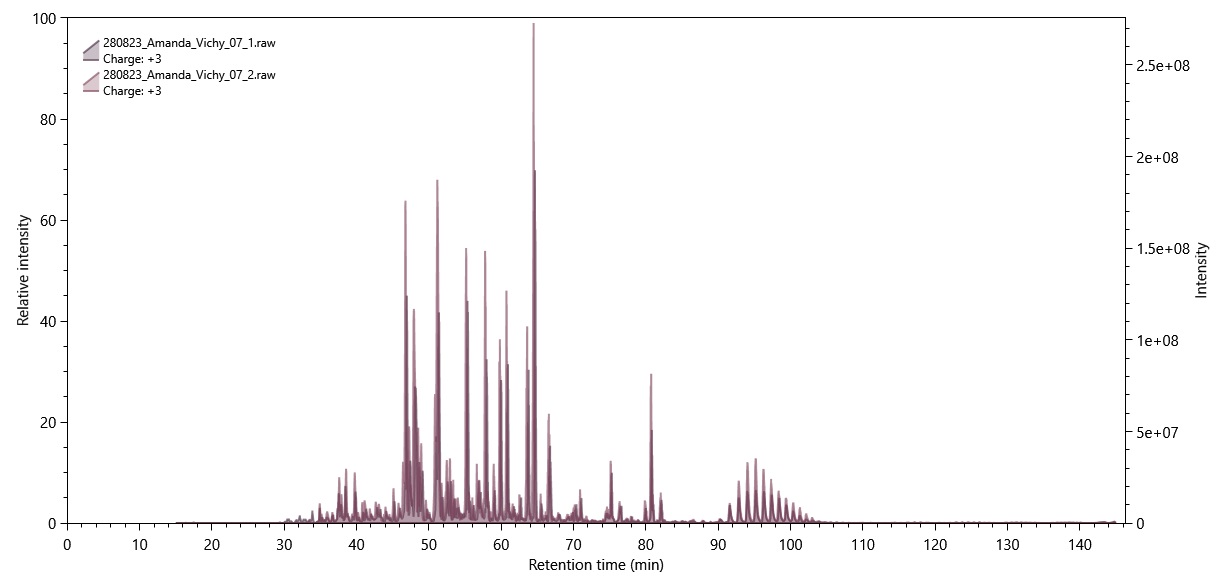

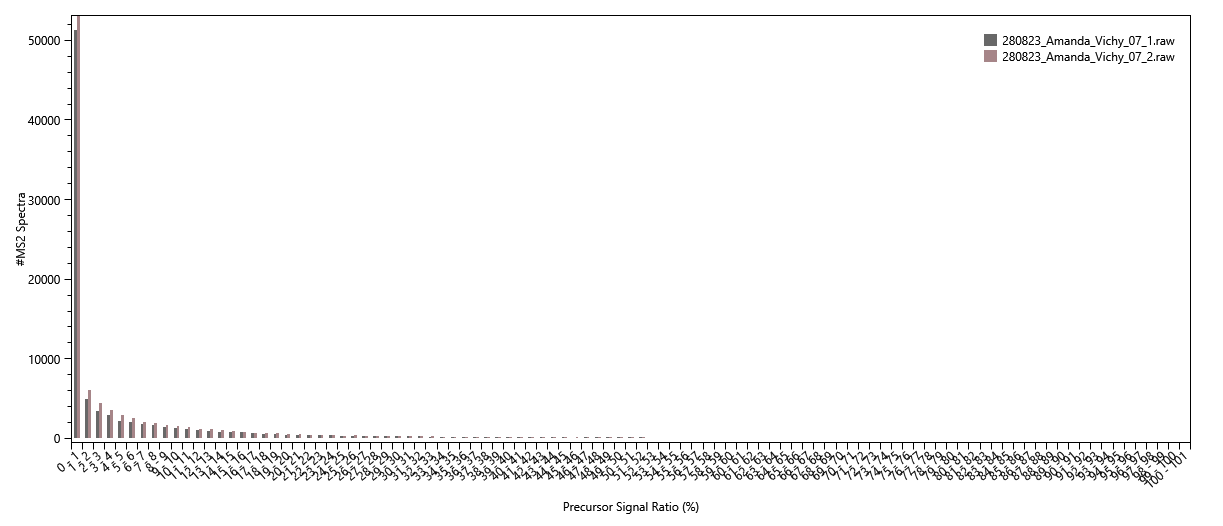

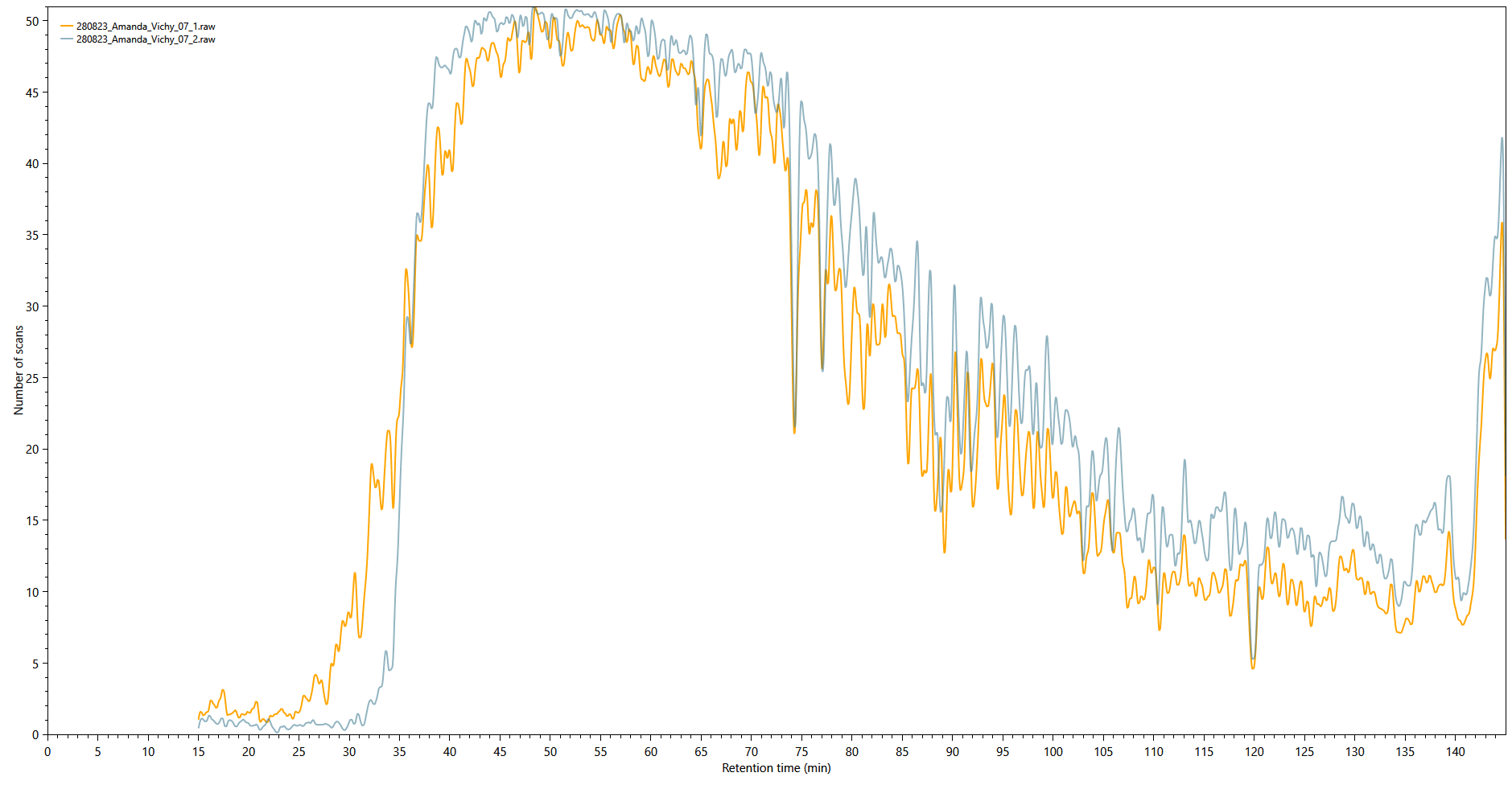

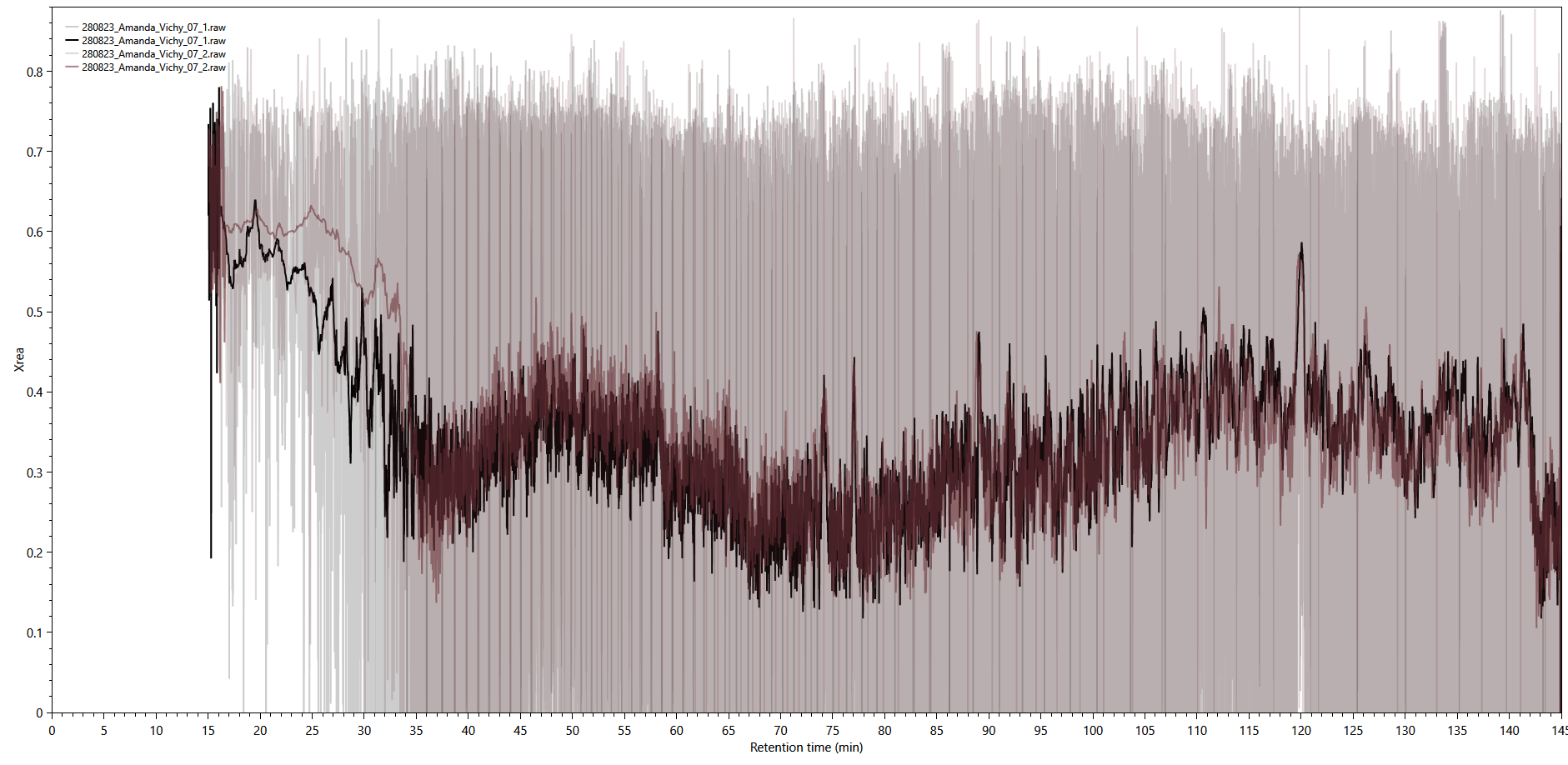
**
- **09**

**
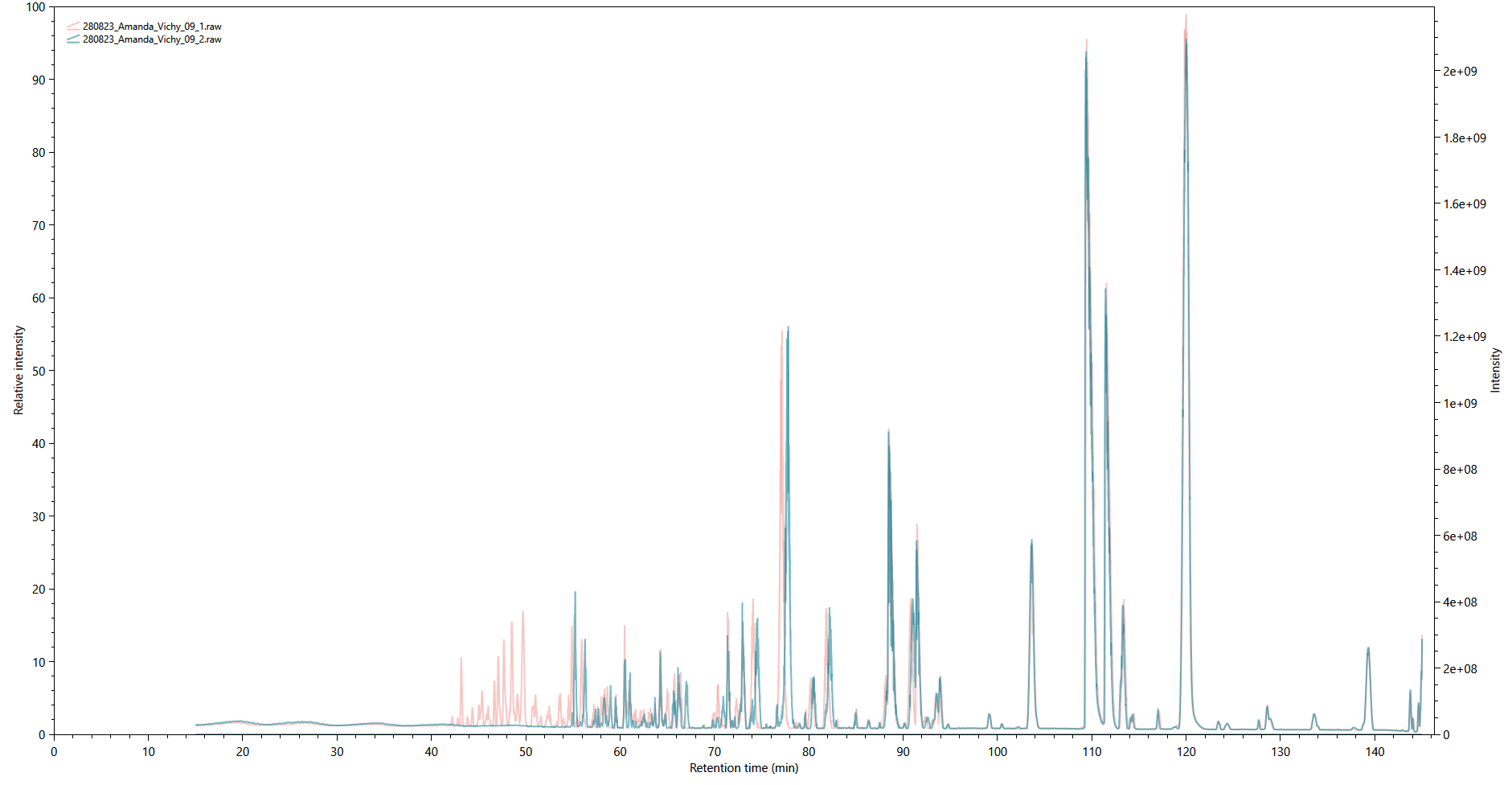

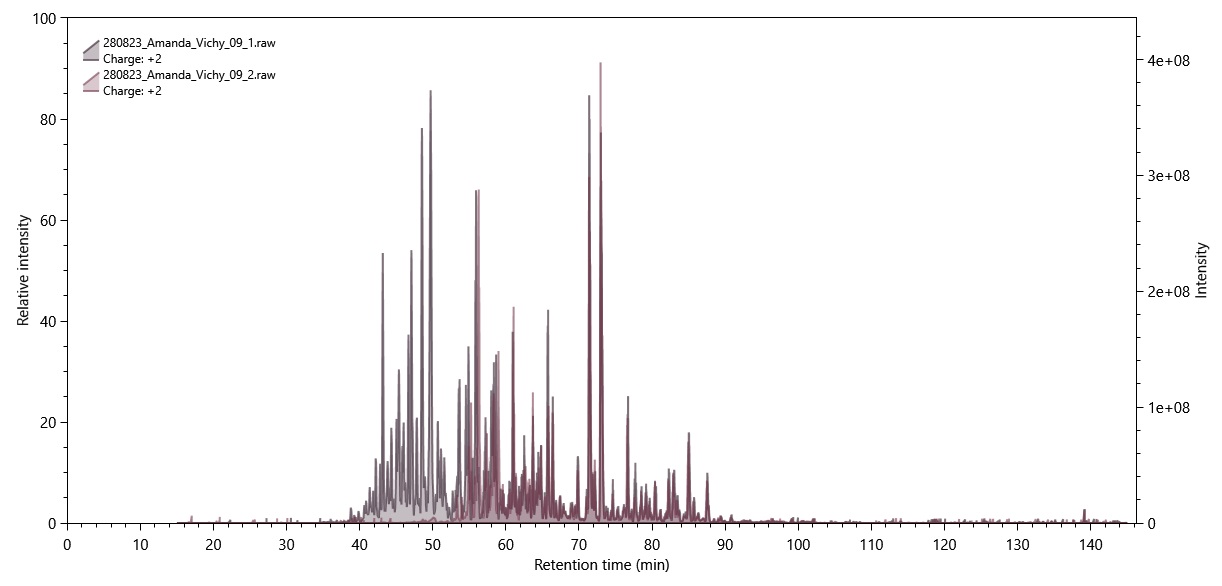

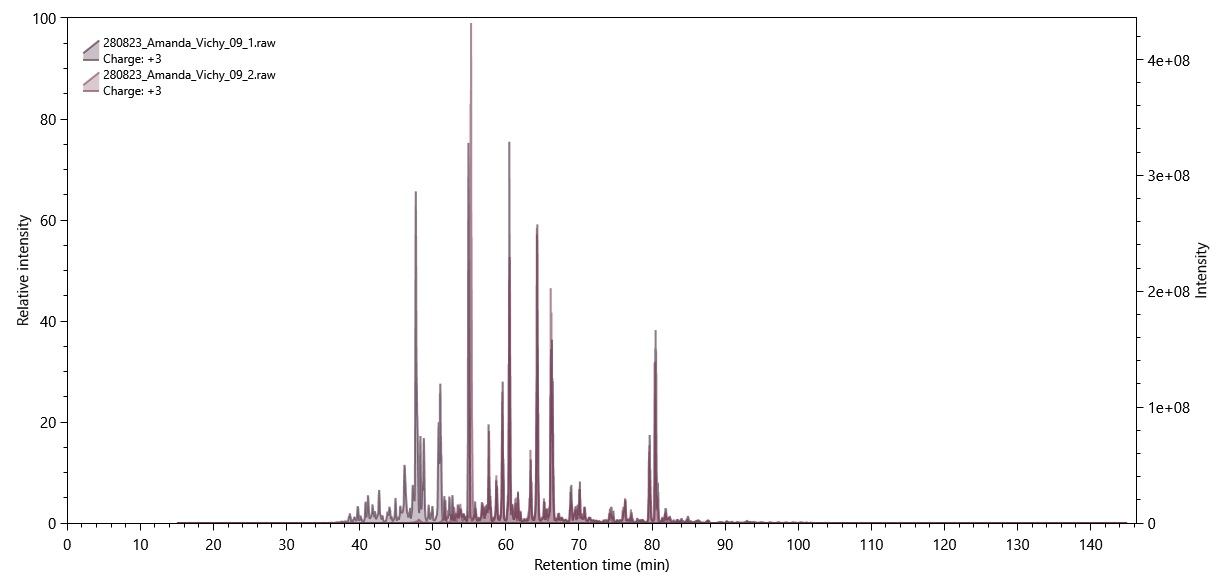

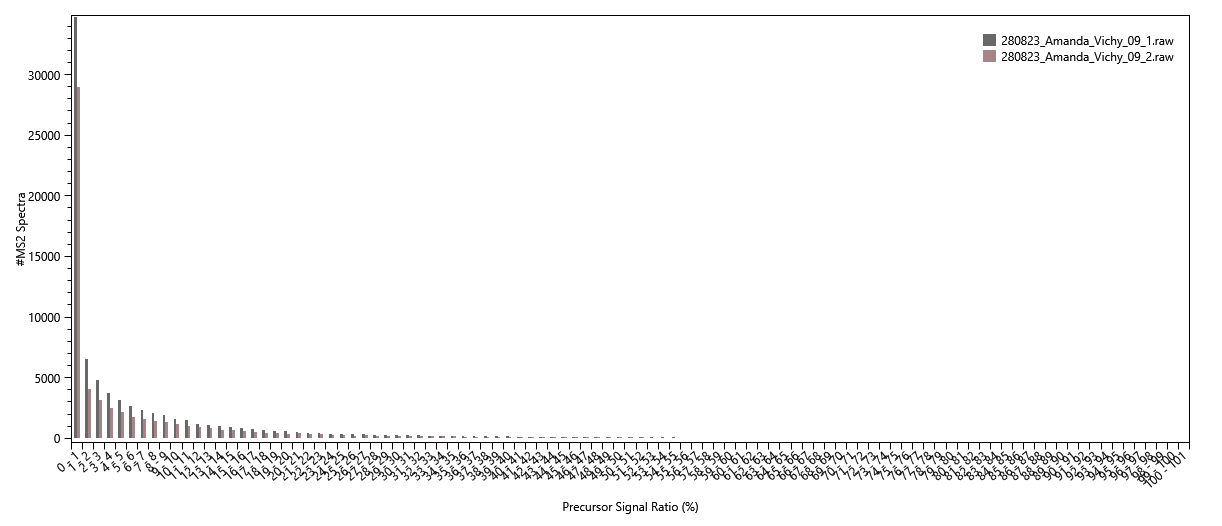

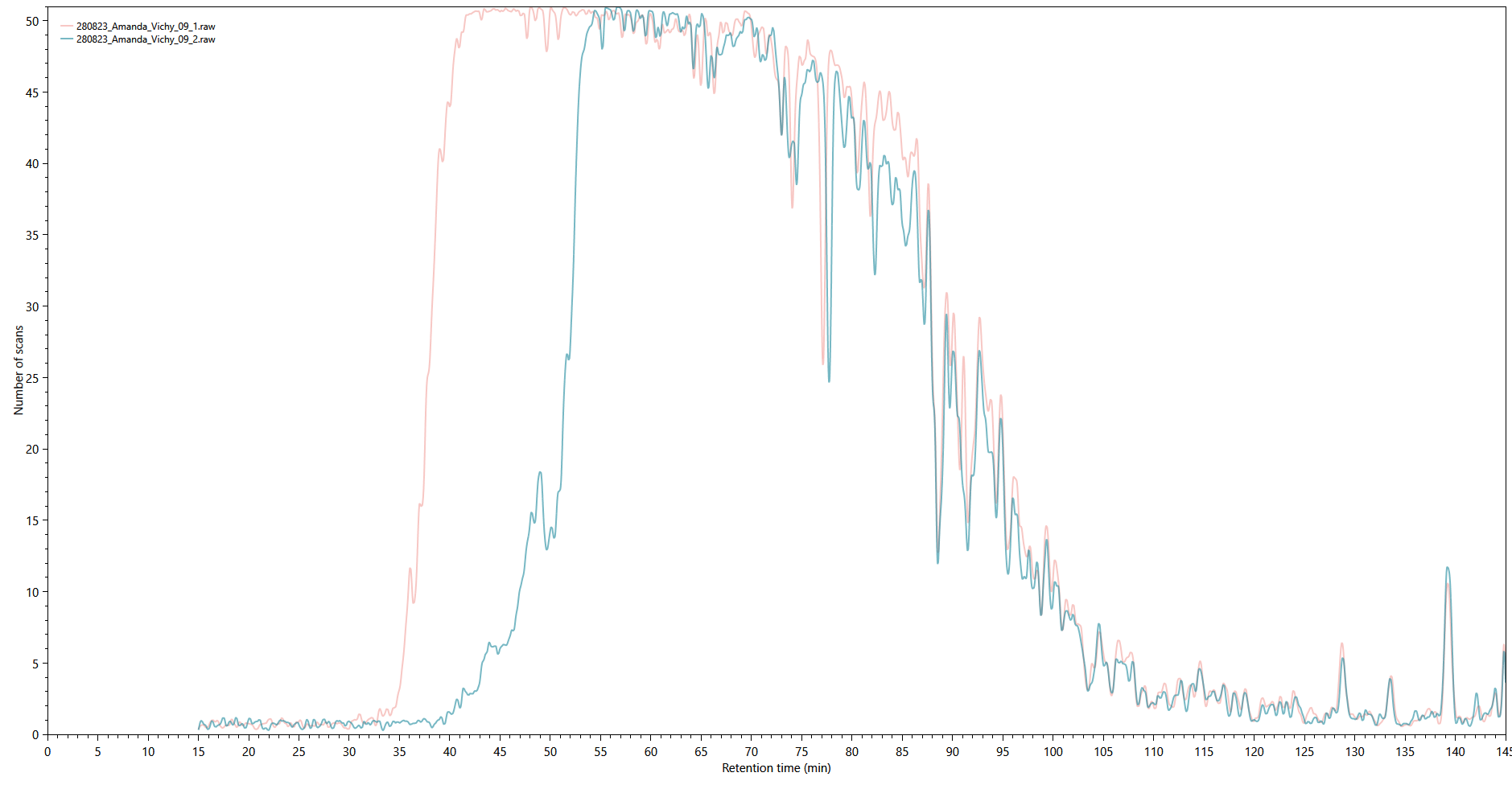

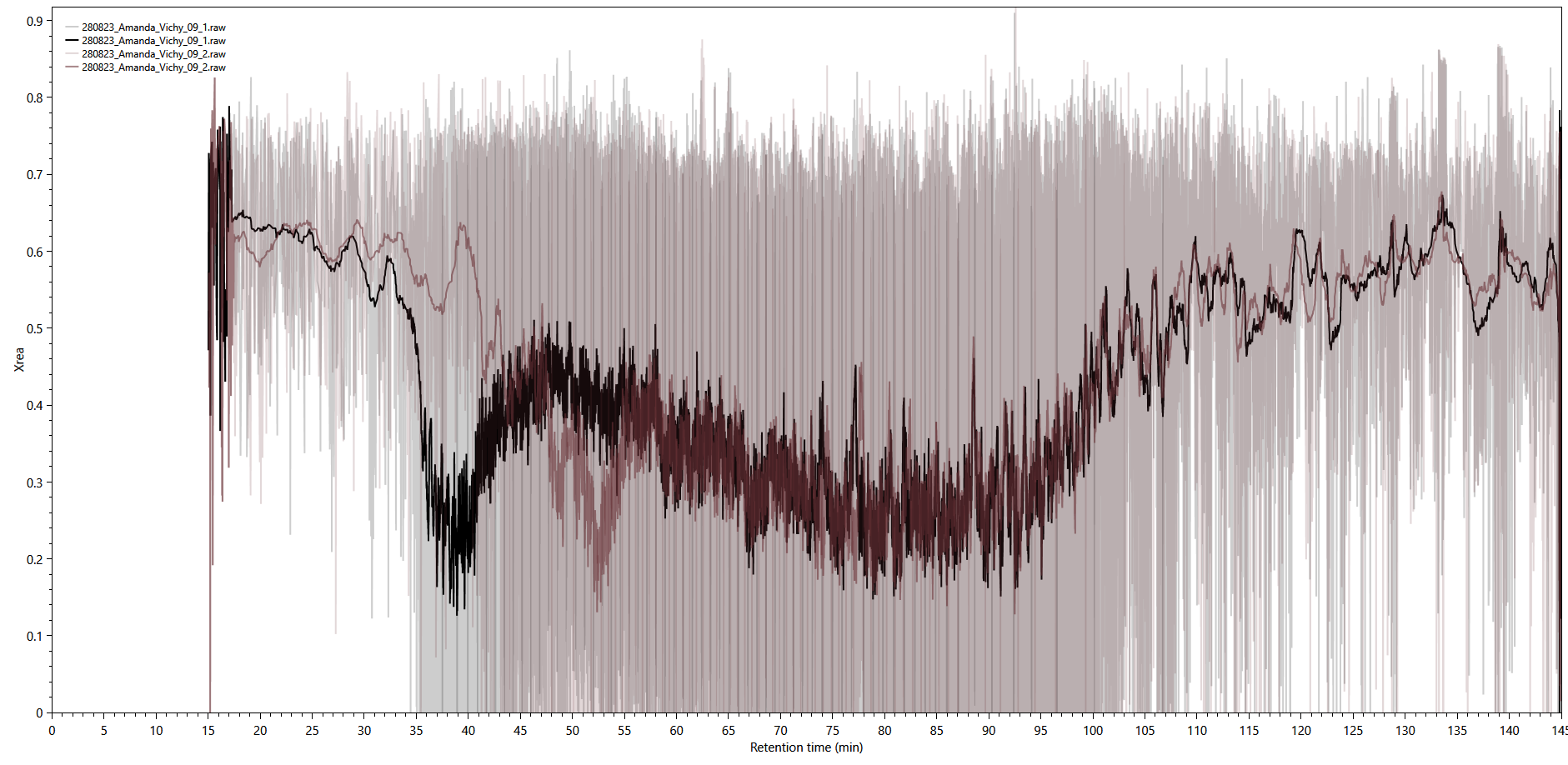
**

- **15**

**
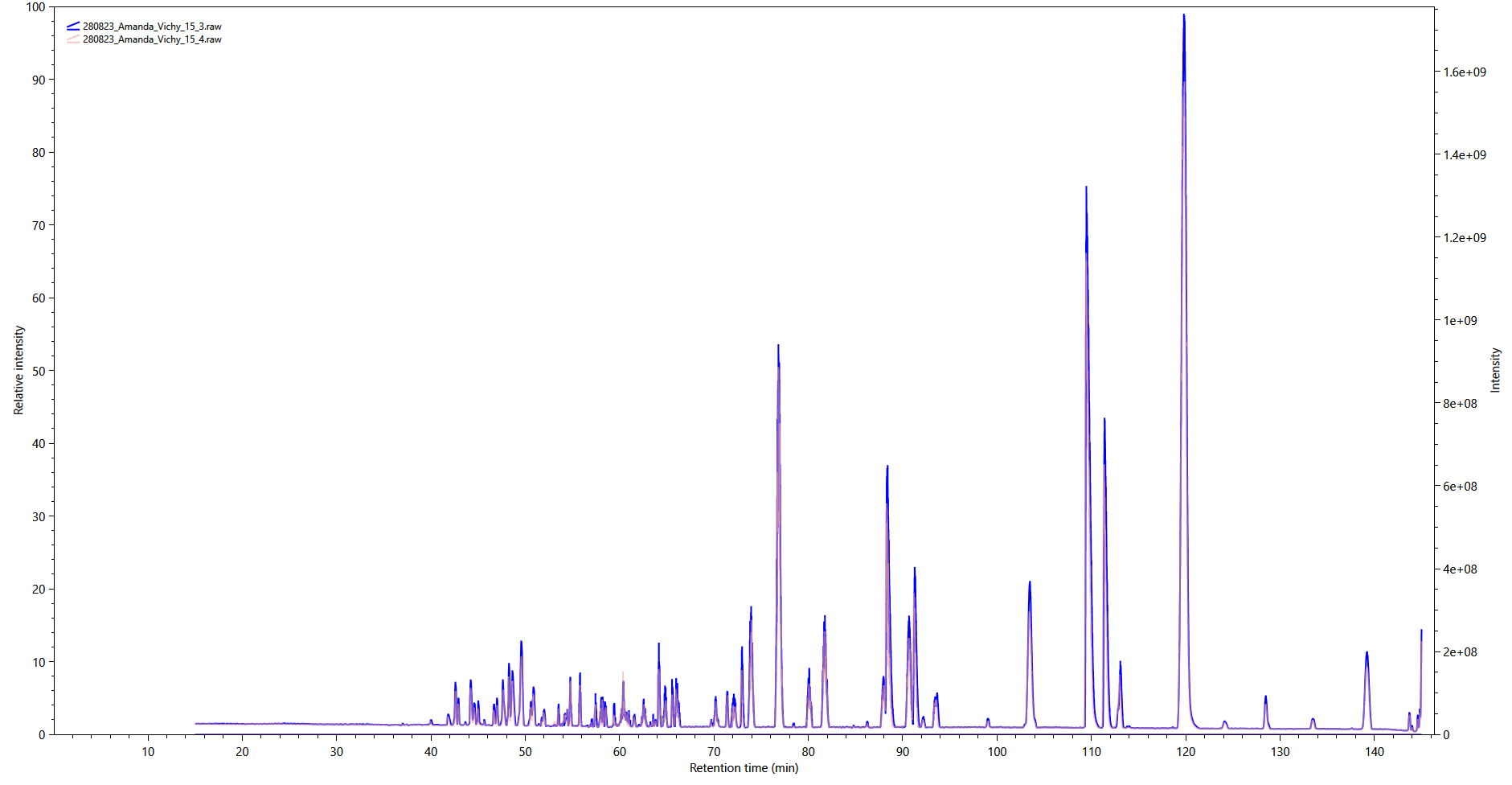

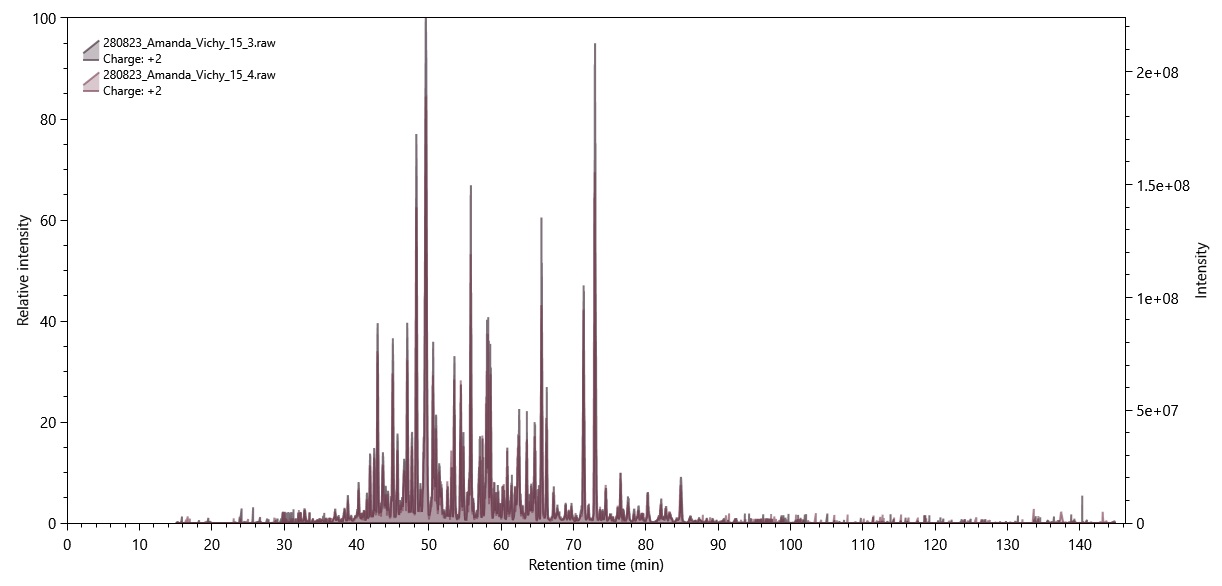

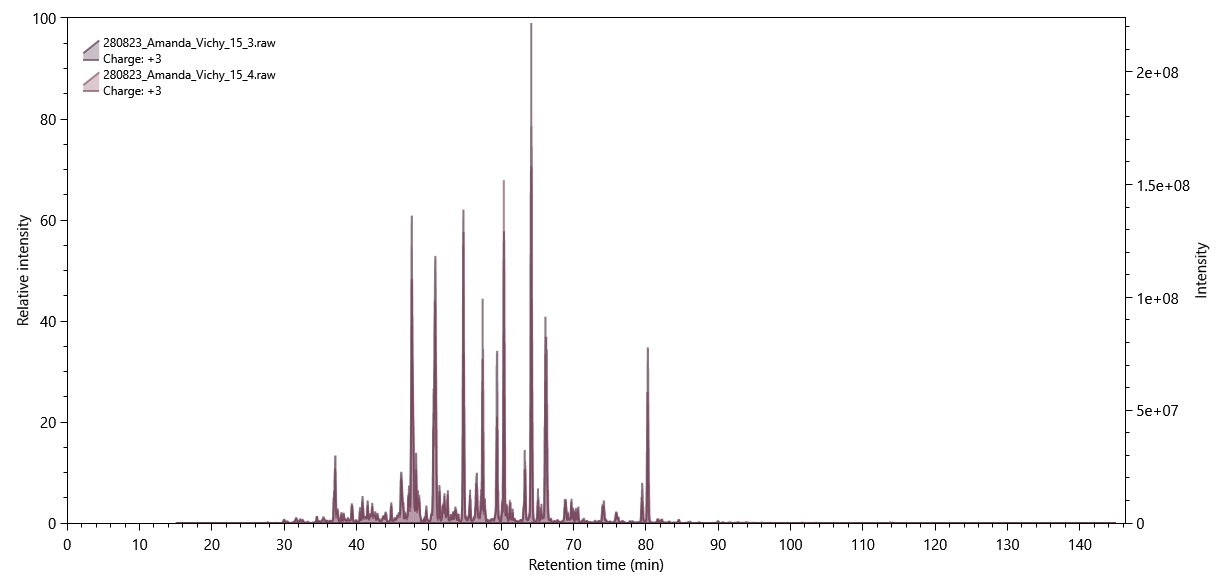

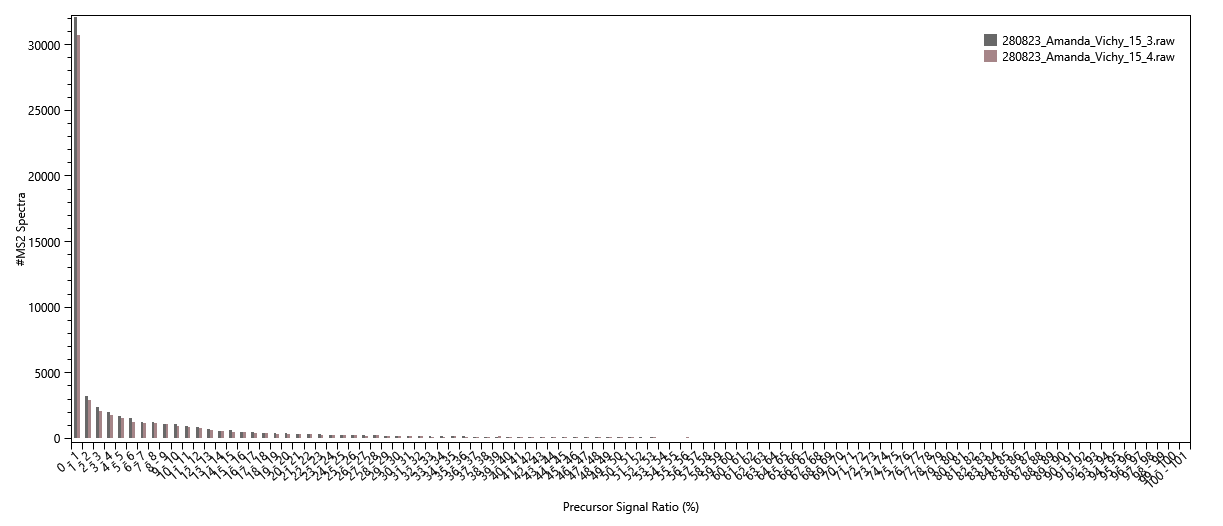

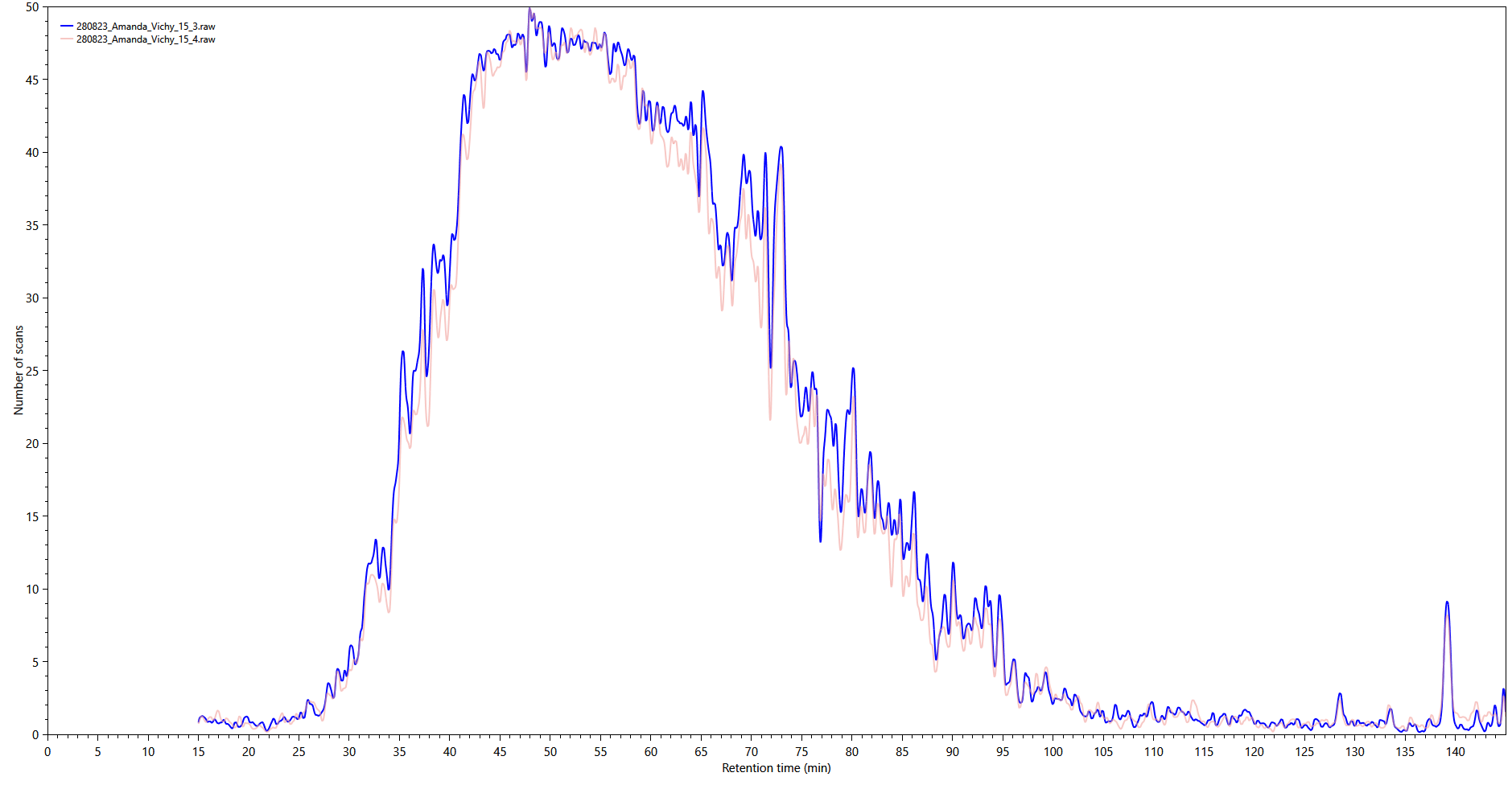

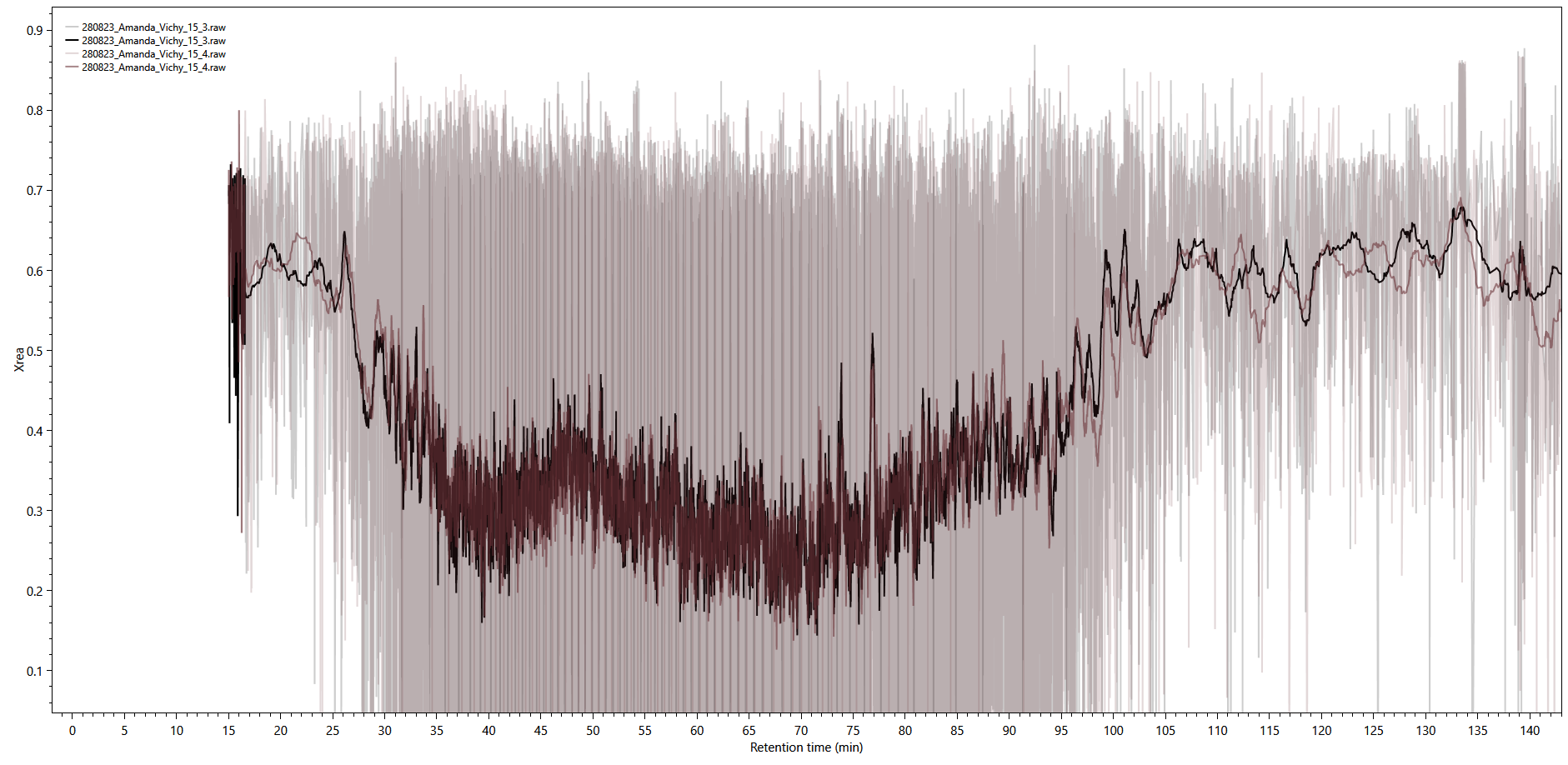
**

- **17**

**
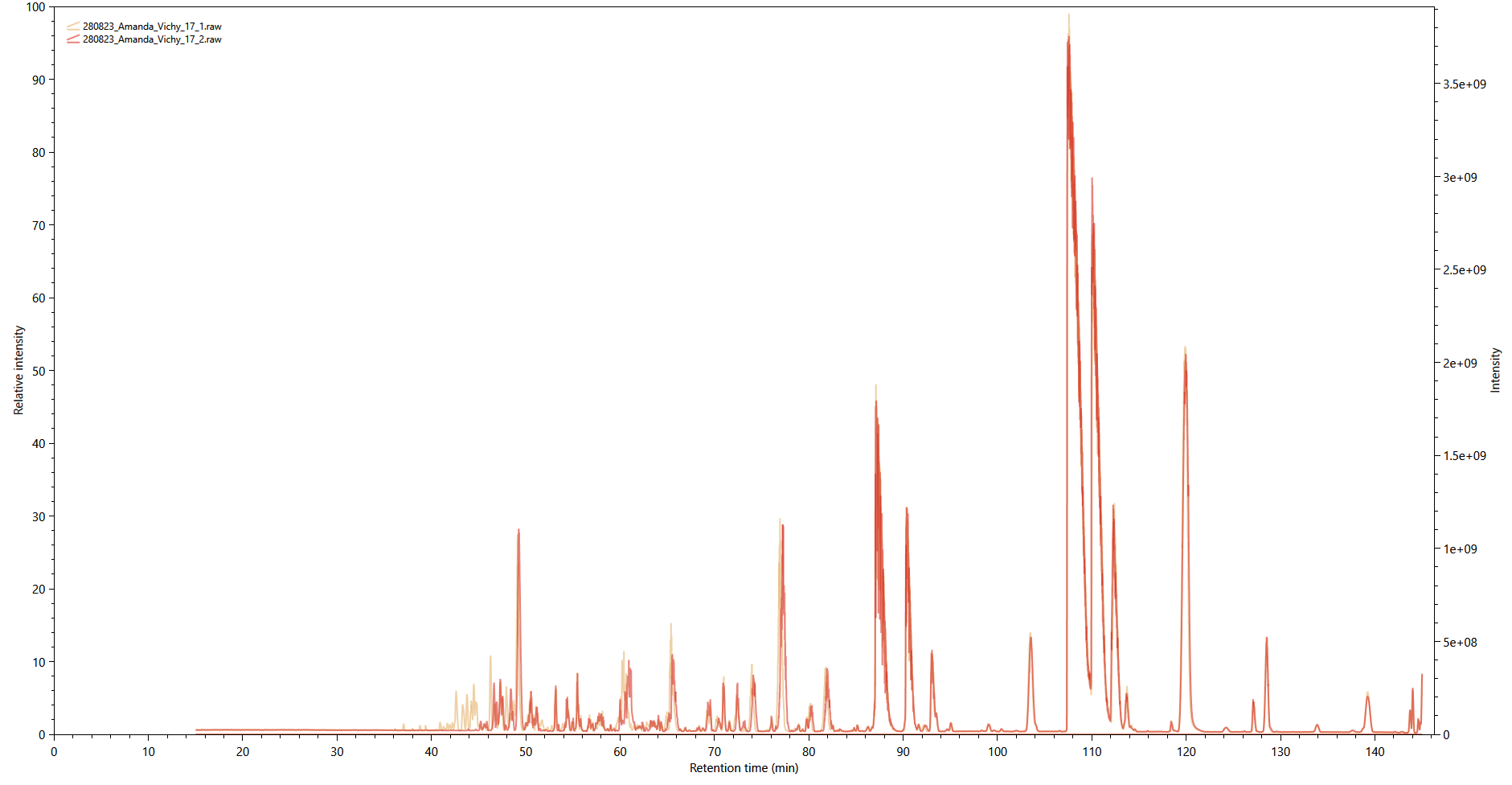

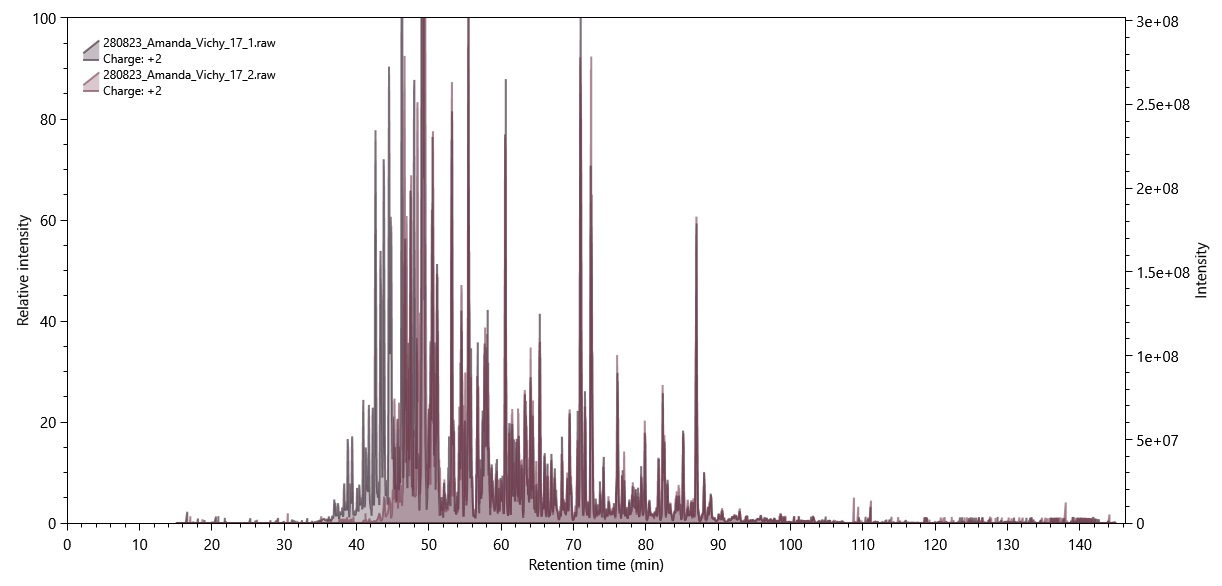

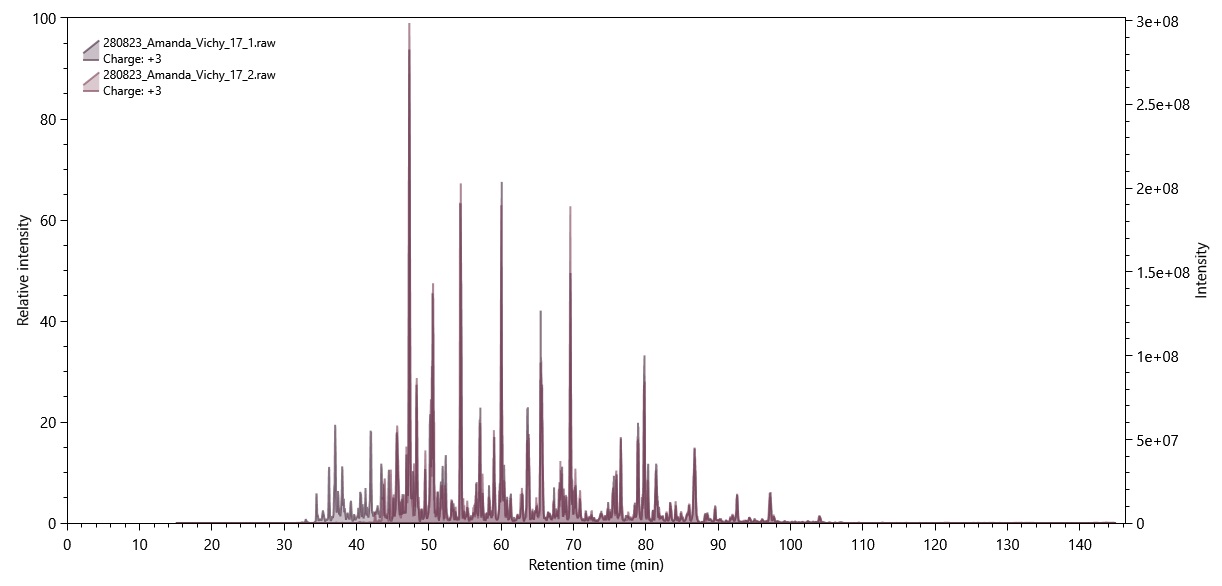

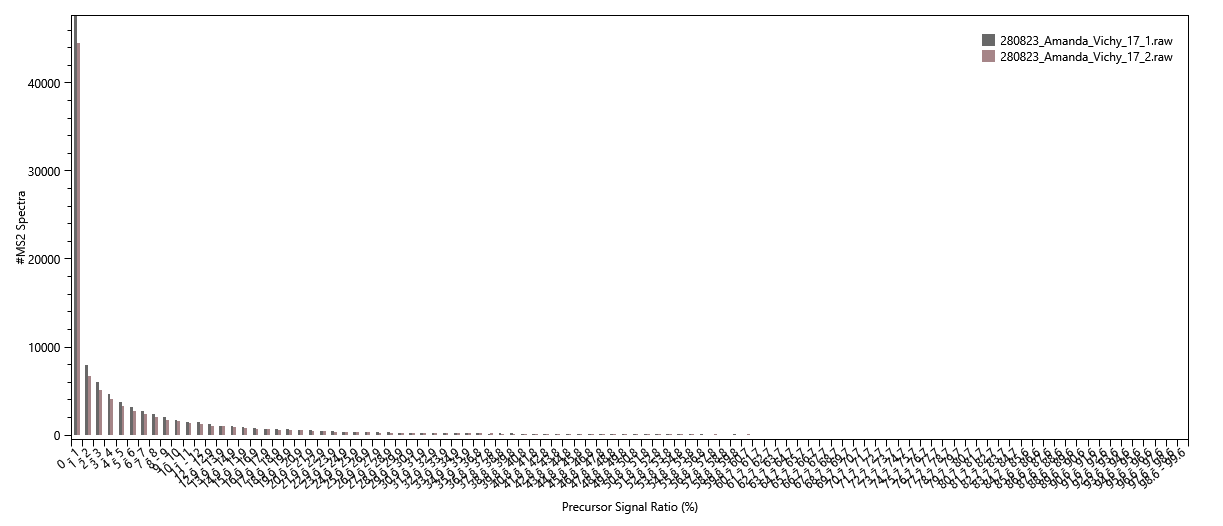

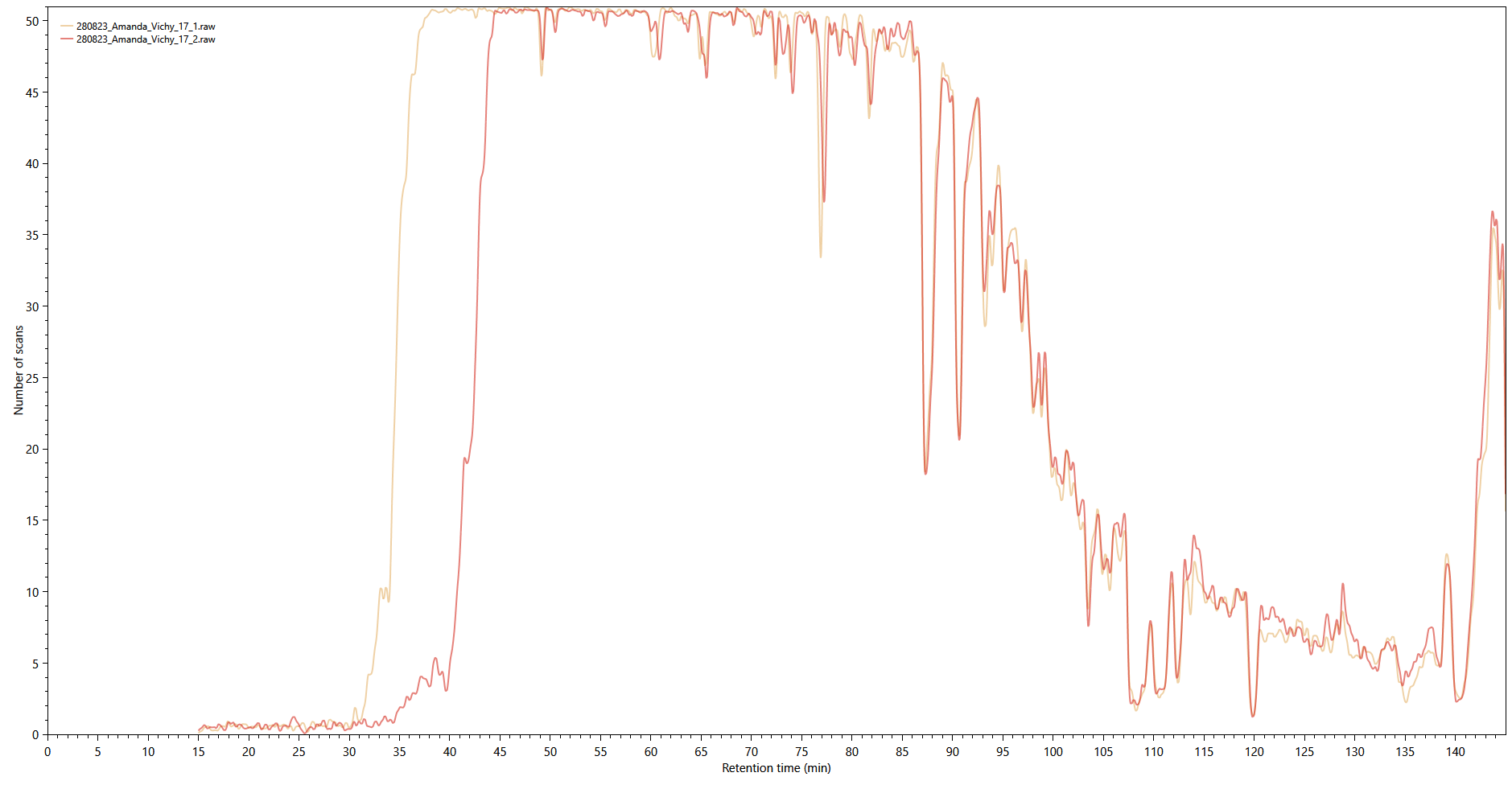

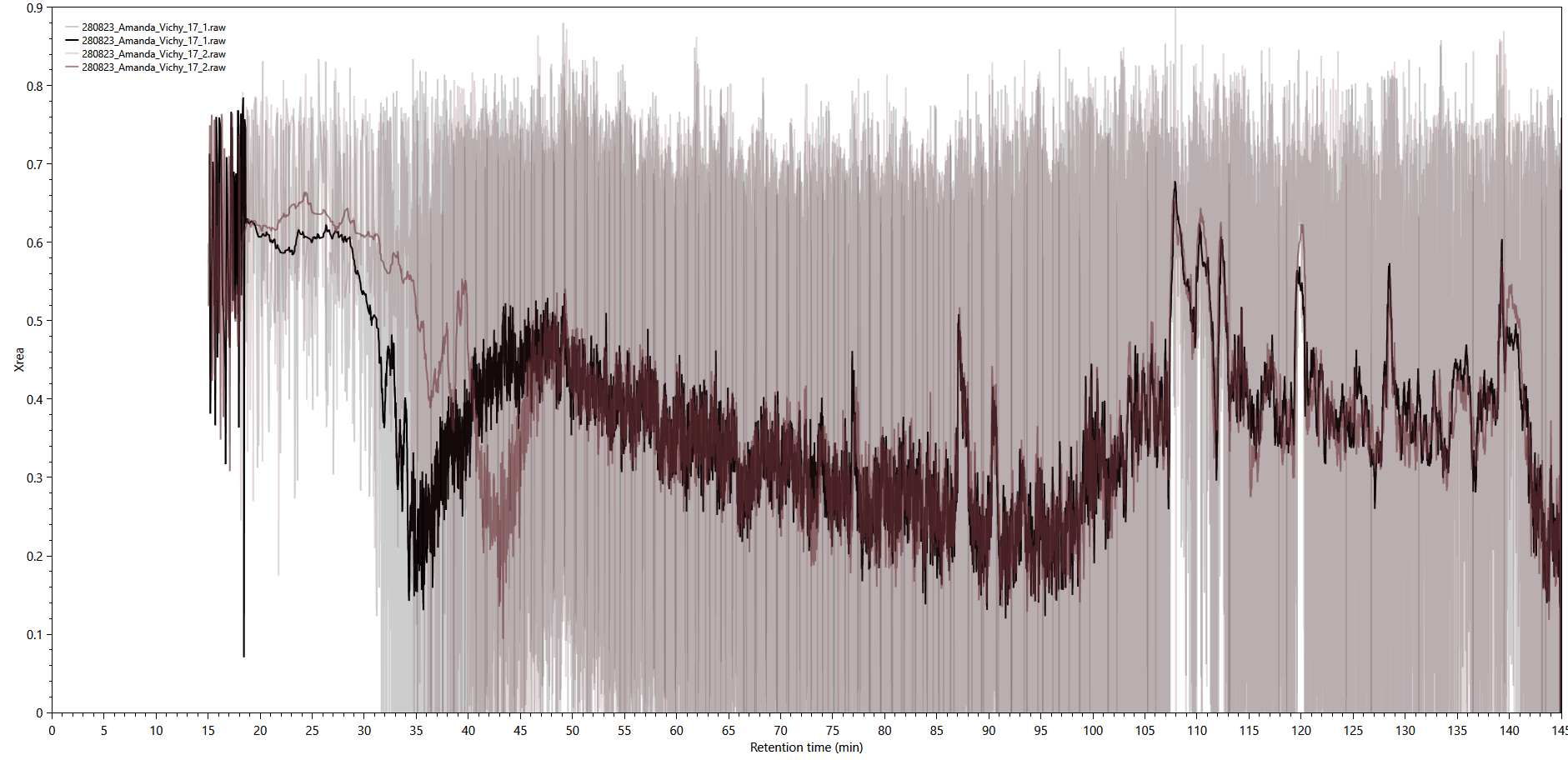
**

- **19
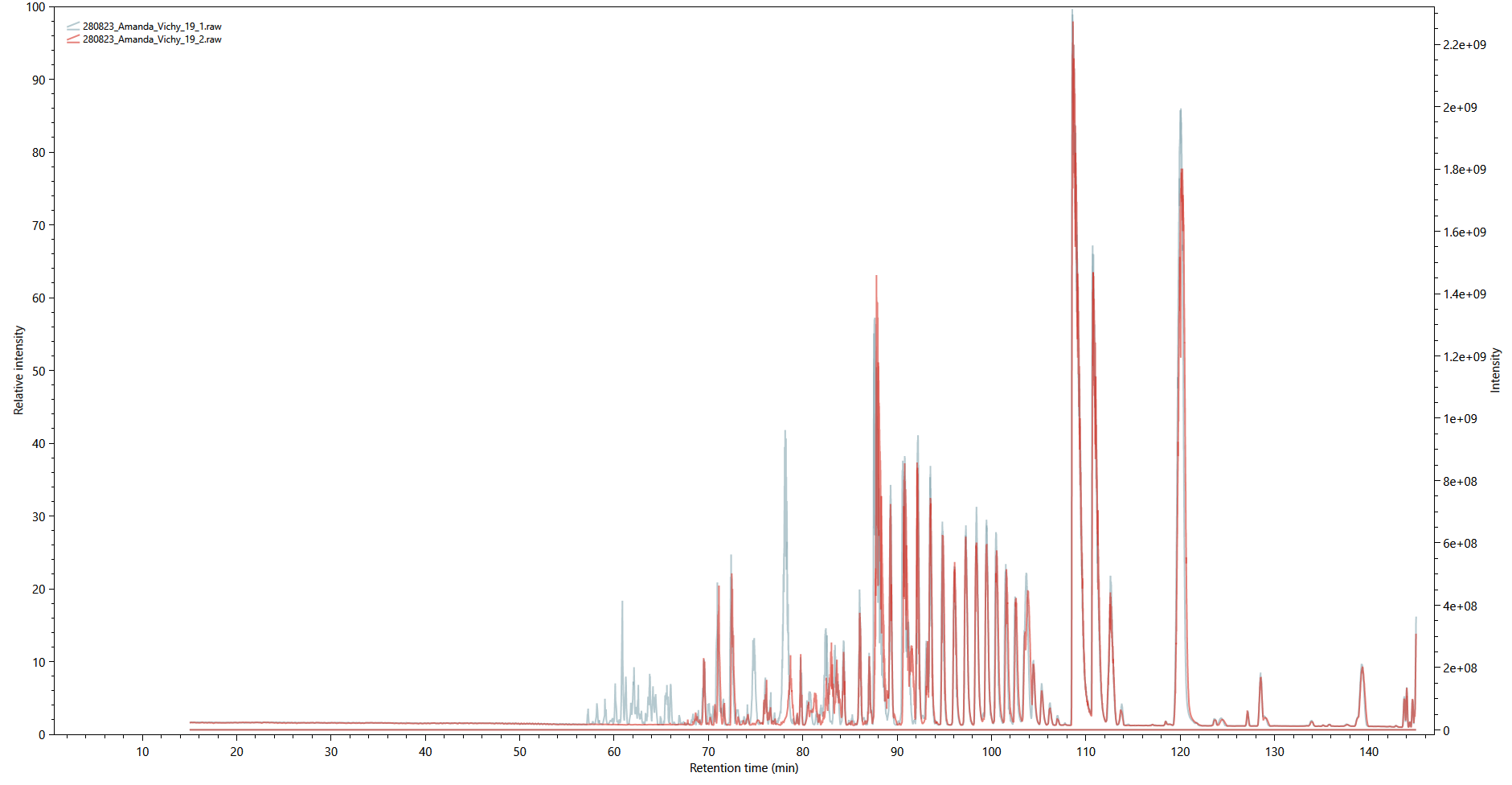

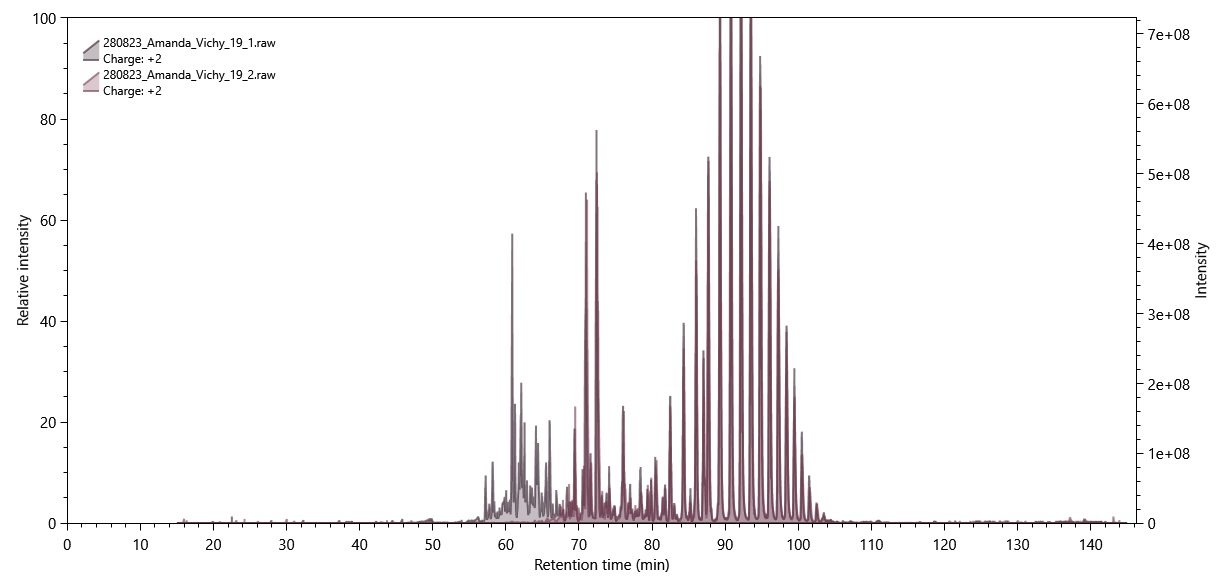

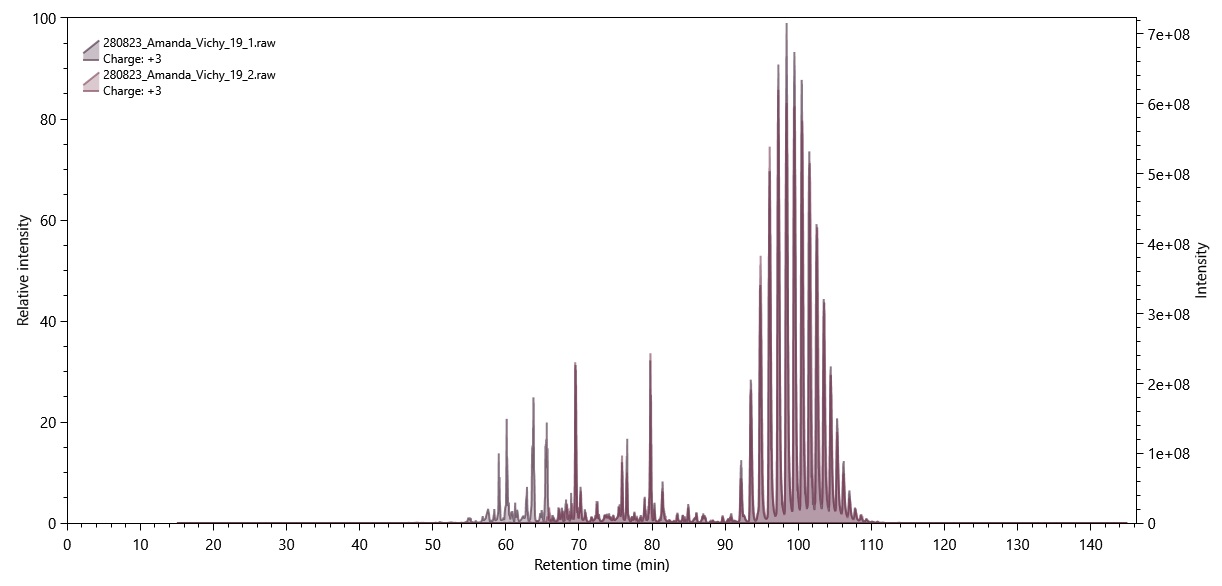

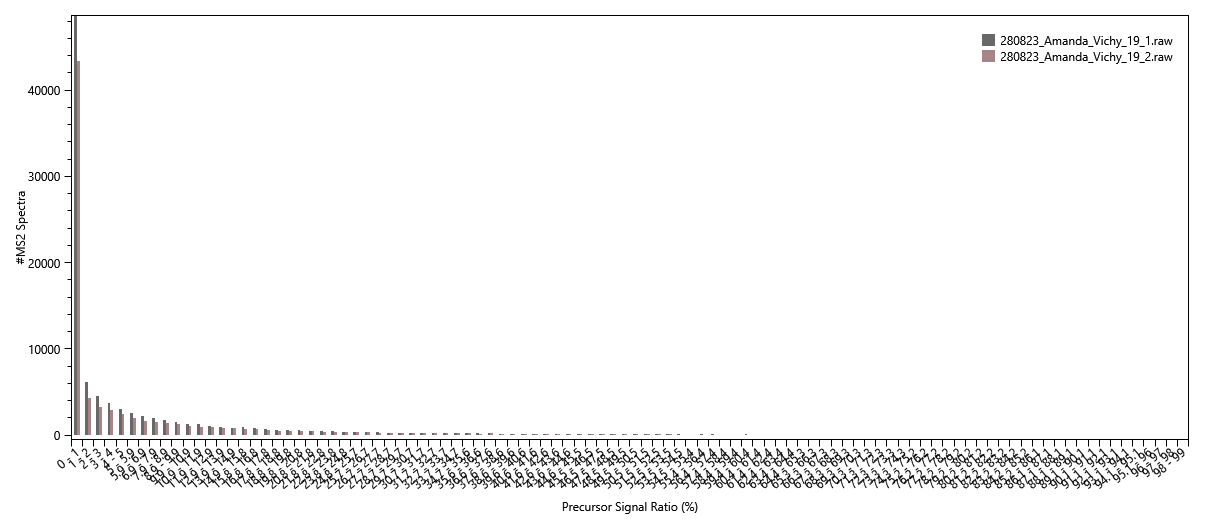

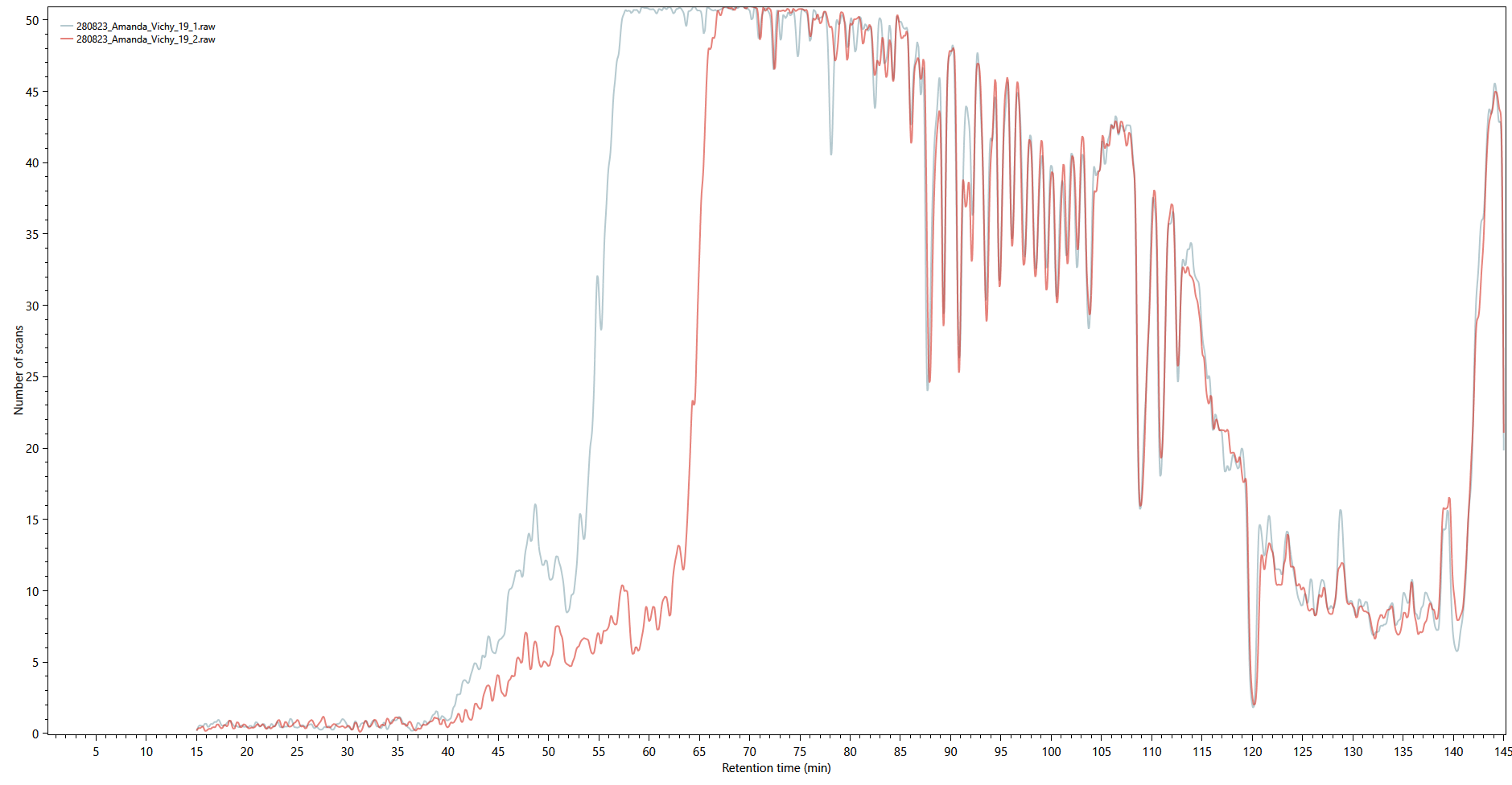

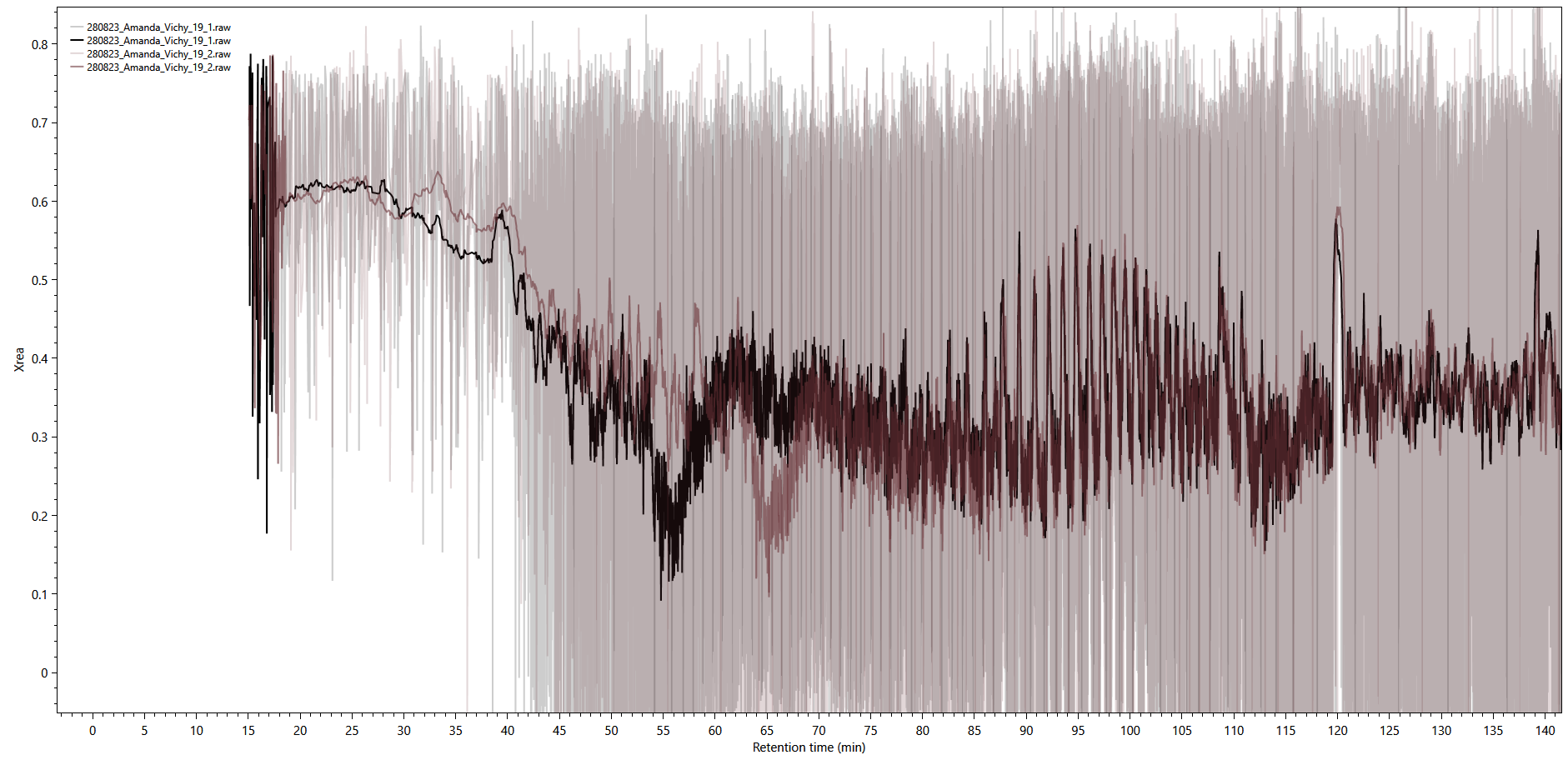
**
- **21
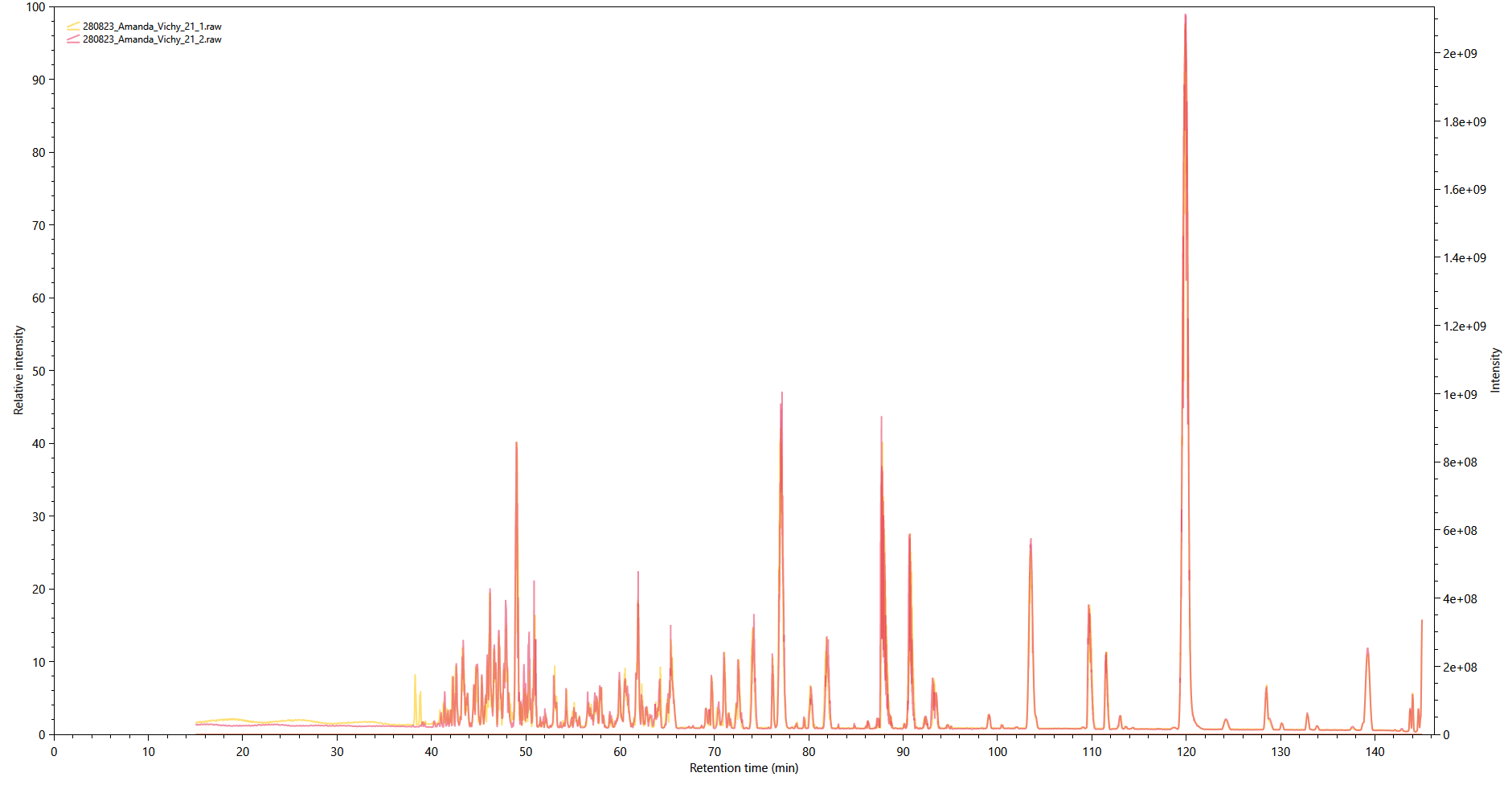

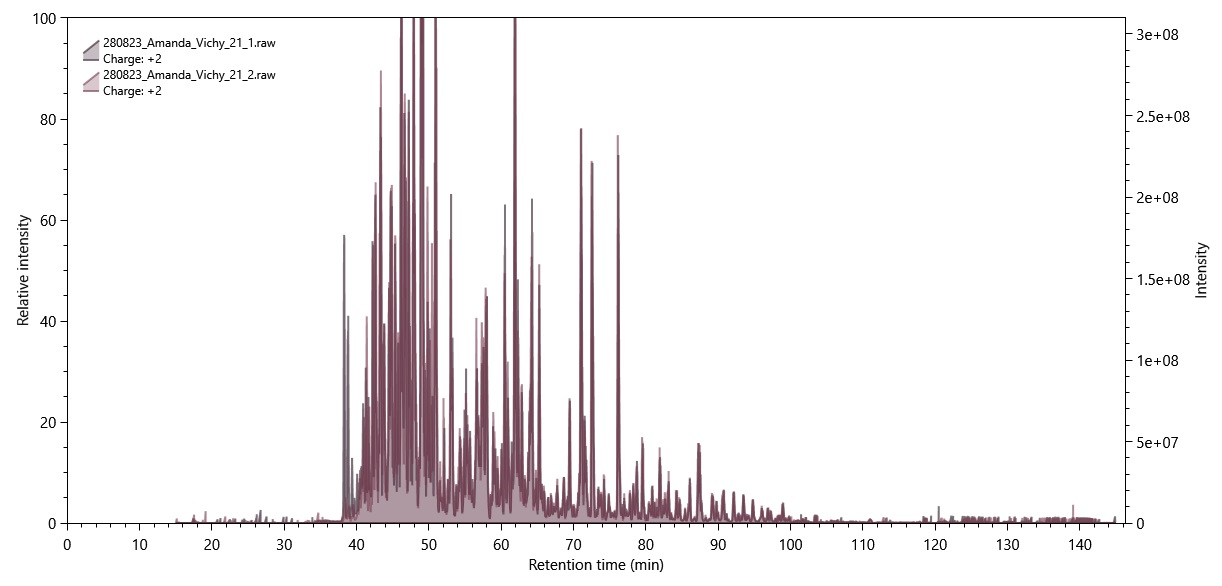

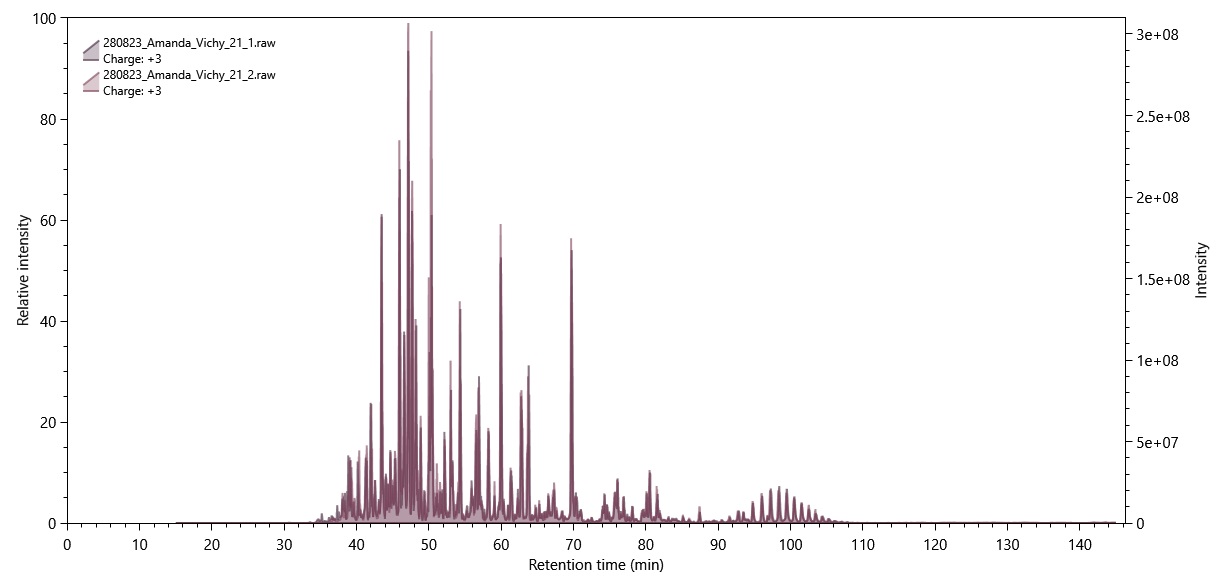

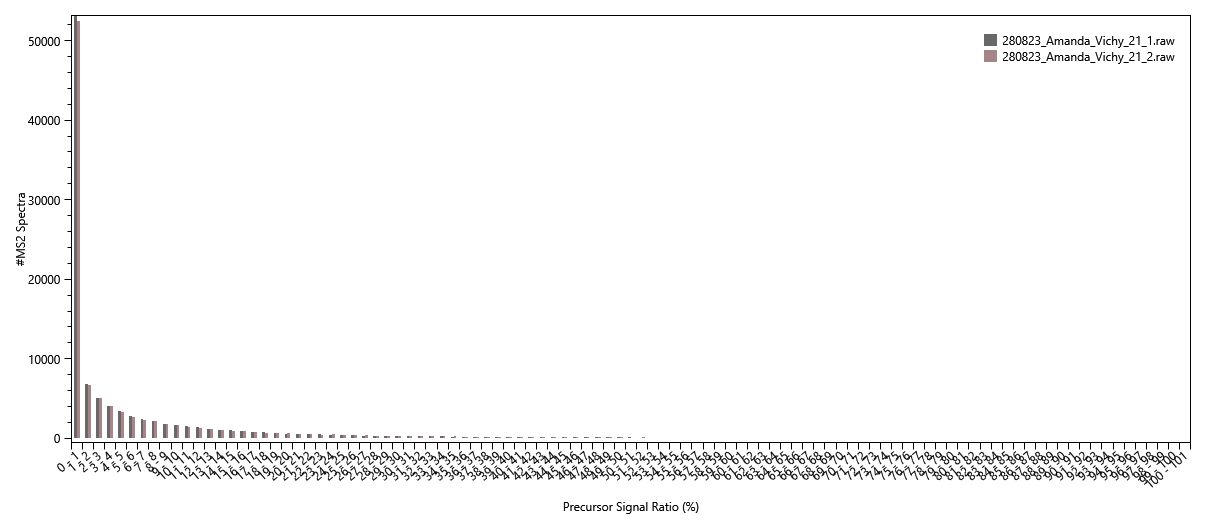

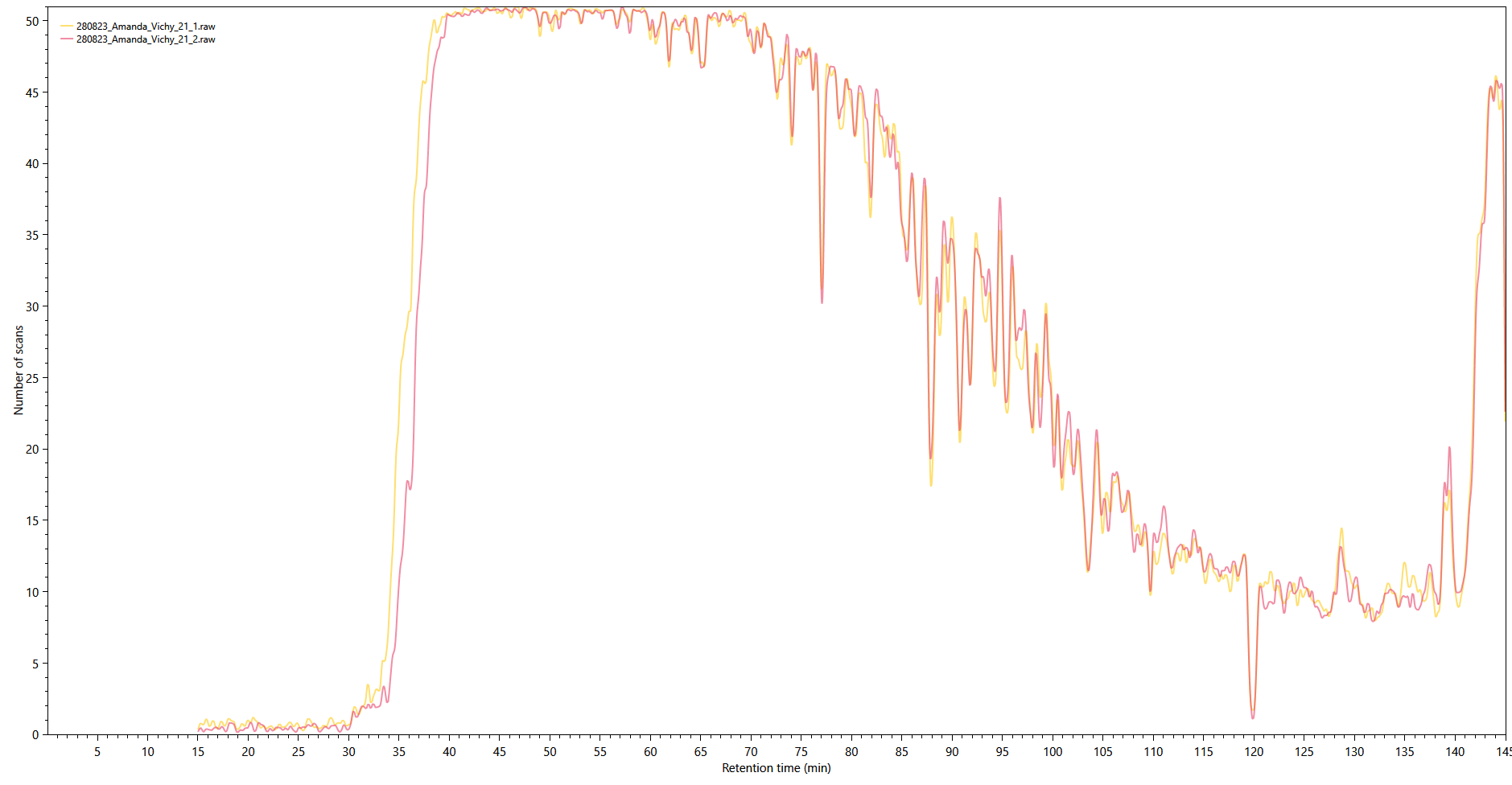

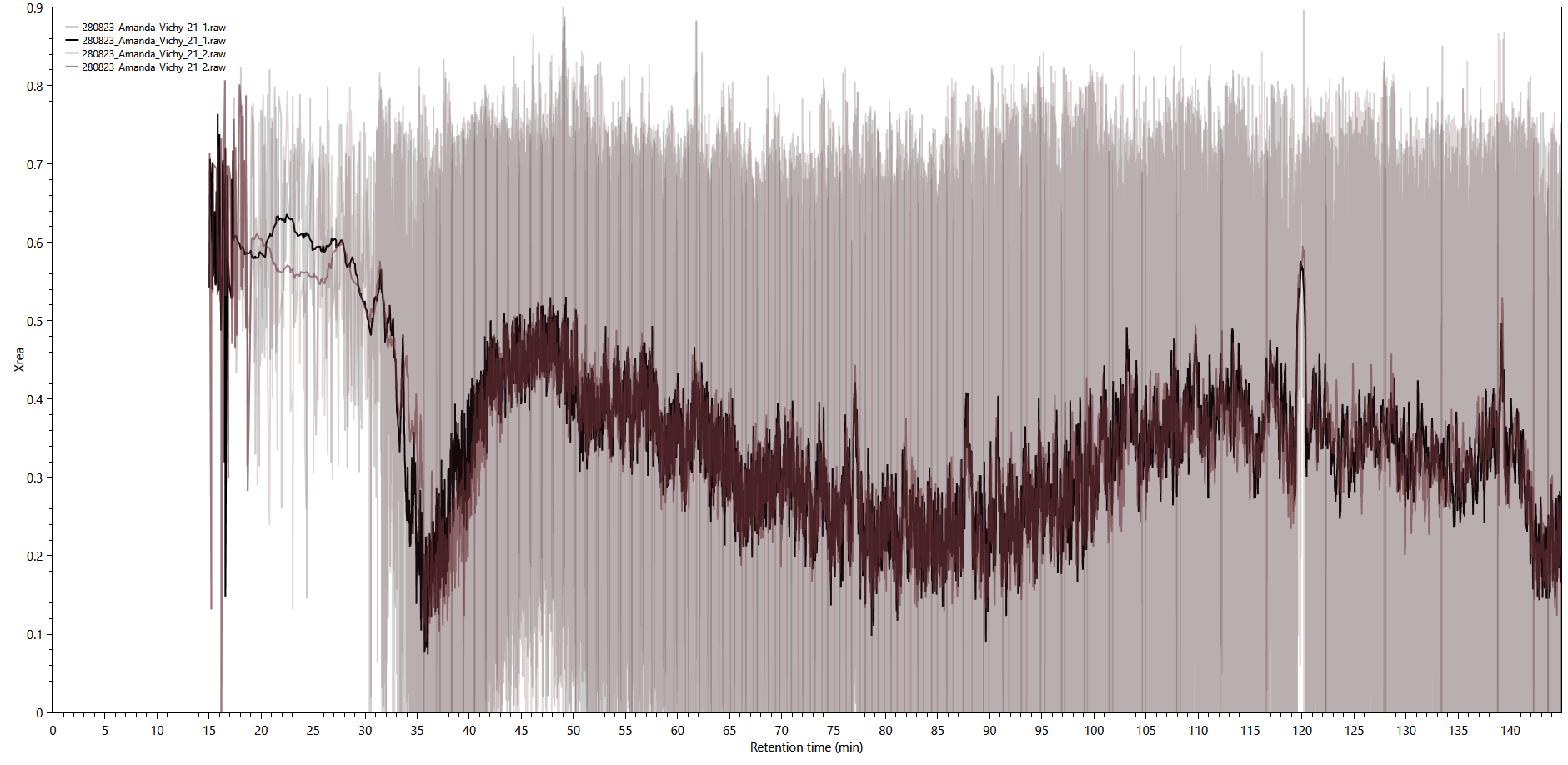
**
- **23**


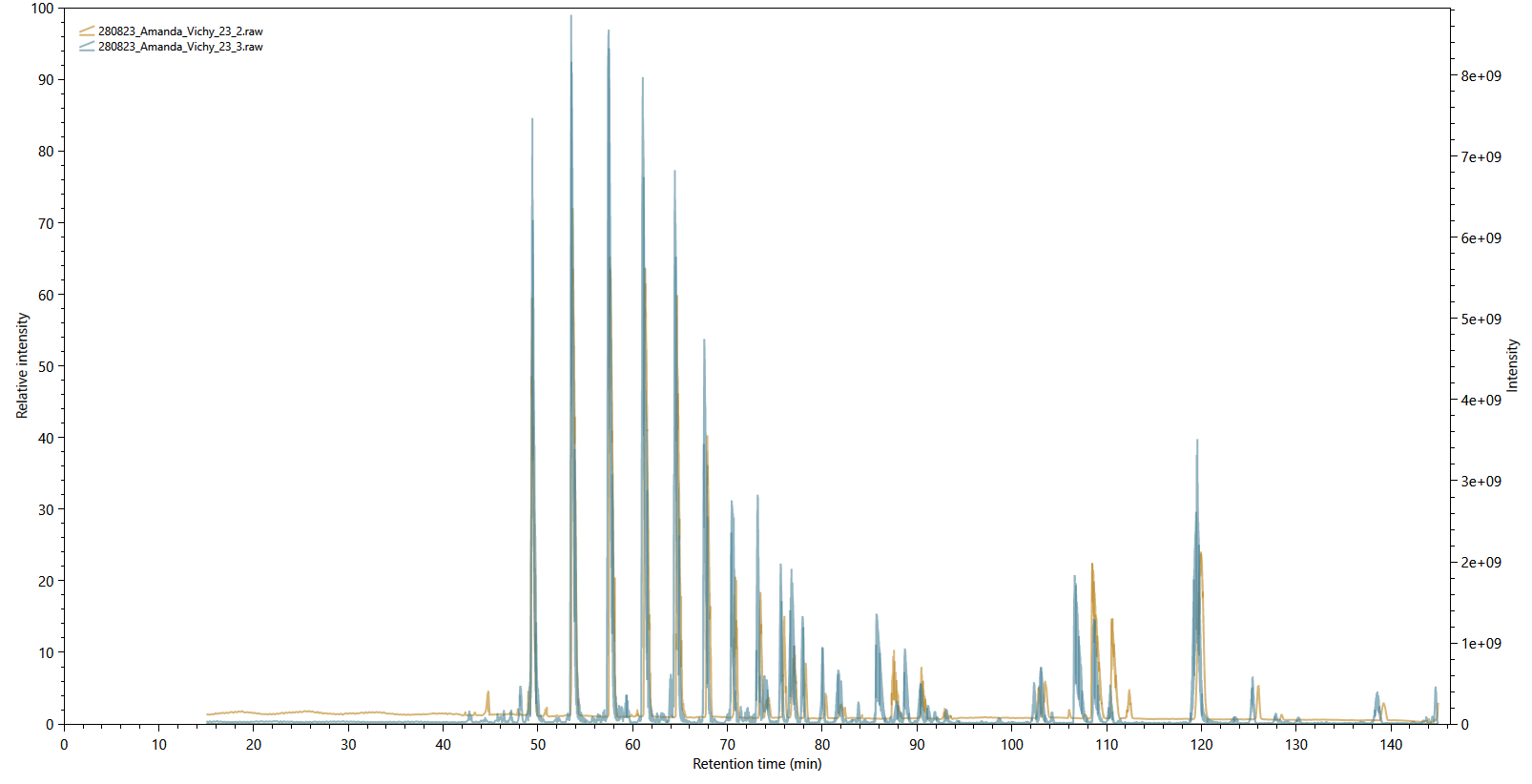


- **29**
- **31**
- **33**
